# Fossil marine vertebrates (Chondrichthyes, Actinopterygii, Reptilia) from the Upper Cretaceous of Akkermanovka (Orenburg Oblast, Southern Urals, Russia)

**DOI:** 10.1016/j.cretres.2023.105779

**Published:** 2024-03

**Authors:** Patrick L. Jambura, Sergey V. Solonin, Samuel L.A. Cooper, Eduard V. Mychko, Maxim S. Arkhangelsky, Julia Türtscher, Manuel Amadori, Sebastian Stumpf, Alexey V. Vodorezov, Jürgen Kriwet

**Affiliations:** a Department of Palaeontology, University of Vienna, Josef-Holaubek-Platz 2, 1090 Vienna, Austria; b Vienna Doctoral School of Ecology and Evolution (VDSEE), University of Vienna, Djerassiplatz 1, 1030 Vienna, Austria; c Department of Geography, Ecology and Natural Management, Ryazan State University named for S. Yesenin, 390000 Ryazan, Russia; d Museum am Löwentor, Staatliches Museum für Naturkunde Stuttgart, 70191 Stuttgart, Germany; e Department of Paleontology, Hohenheim University, 70599 Stuttgart, Germany; f Shirshov Institute of Oceanology, Russian Academy of Sciences, 117997 Moscow, Russia; g Museum of the World Ocean, 236006 Kaliningrad, Russia; h Institute of Living Systems, Immanuel Kant Baltic Federal University, 236016 Kaliningrad, Russia; i Borissiak Paleontological Institute, Russian Academy of Sciences, 117997 Moscow, Russia; j Department of General Geology and Minerals, Saratov State University, 410012 Saratov, Russia; k Department of Oil and Gas, Saratov State Technical University, 410054 Saratov, Russia

**Keywords:** Elasmobranchii, Teleostei, Lepisosteidae, Mosasauridae, Polycotylidae, Santonian–?early Campanian

## Abstract

Upper Cretaceous coastal marine deposits are widespread in the Southern Urals with a number of marine vertebrates previously reported from this region. However, previous studies on the vertebrate faunas in this region often lack detailed taxonomic descriptions and illustrations, rendering comparisons to other faunal assemblages difficult. A new diverse vertebrate assemblage comprising cartilaginous and bony fishes, as well as marine reptiles, is described here from the Orenburg region near Akkermanovka (Southern Urals, Russia). Thirty five taxa are identified, including three holocephalans (*Elasmodus* sp., *Ischyodus yanschini*, Chimaeroid indet.), two hybodontiform sharks (*Meristodonoides* sp., cf. *Polyacrodus* sp.), 17 neoselachians (*Paraorthacodus* cf. *andersoni, Paraorthacodus* sp., *Synechodus* sp., *Cederstroemia nilsi, Acrolamna acuminata, Archaeolamna* ex gr. *kopingensis, Cretalamna sarcoportheta, Cretoxyrhina mantelli, Eostriatolamia segedini, E. venusta, Hispidaspis horridus, H*. cf. *gigas, Pseudocorax laevis, Pseudoscapanorhynchus compressidens, Scapanorhynchus rhaphiodon, Squalicorax kaupi, Ptychodus rugosus*), a holostean (Lepisosteidae indet.), nine teleosts (*Protosphyraena* sp., Saurodontidae indet., cf. *Pachyrhizodus* sp., Pachyrhizodontidae indet., *Enchodus petrosus, E. ferox, E*. cf. *gladiolus, E*. spp., Alepisauroidei indet.), two plesiosaurs (Polycotylidae indet., Plesiosauria indet.), and one mosasaurid (Tylosaurinae indet.). Based on the faunal assemblage, a Santonian–?early Campanian age is proposed. Lamniform sharks are the best represented group in terms of taxic diversity and relative abundance, probably reflecting the peak in diversity this group experienced following the Cenomanian radiation in the Late Cretaceous. The faunal assemblage of Akkermanovka exhibits significant taxonomic overlaps with assemblages reported from Asia and North America, but not from Southern Hemisphere continents, indicating east–west dispersal of several marine taxa during the Late Cretaceous.

## 1 Introduction

During the Late Cretaceous, the southeast European regions of Russia were covered by a vast shallow sea representing the northern periphery of the Tethys. It reached its maximum transgression during the early Campanian and had connections with the West Siberian cold sea through a series of straits, which contributed to an exchange of warm and cold water masses between both seas (Baraboshkin et al., 2003). The Upper Cretaceous deposits in the Orenburg Oblast are remnants of these palaeostraits with the vertebrate remains reported from this region providing important insights into this dynamic and unique palaeoenvironment.

Historically, several new species of marine reptiles (i.e., plesiosaurs and mosasaurids) were described from the Orenburg Region (Bogolyubov, 1910, 1911, 1912a,b). Due to the fragmentary nature of the material, considerable doubt exists about the validity of these species, and many are now considered to be *nomina dubia* and are left in open nomenclature (Storrs et al., 2000). Recently, new vertebrate-rich deposits were discovered in the Izhberda quarry (close to the city Orsk), yielding the most complete Late Cretaceous plesiosaur skeleton from the Southern Urals to date (Efimov et al., 2016); it was assigned to the new species *Polycotylus sopozkoi* Efimov, Meleshin and Nikiforov, 2016. Also in these vast coastal marine deposits, rare finds of terrestrial vertebrates, i.e., remains of dinosaurs (Dinosauria indet., Iguanodontia indet.) and pterosaurs (Azhdarchidae indet.) are reported (Averianov et al., 2021a,b, 2022; Skutschas et al., 2022). A preliminary account of the Cretaceous elasmobranchs from this locality was provided in the form of a conference abstract by Los (2021), however this author did not provide any descriptions or illustrations of the listed species.

The study of Cretaceous deposits near Akkermanovka in the Orenburg Oblast began relatively recently, and only preliminary reports on the regional fossil chondrichthyan fauna are available (Popov et al., 2019; Lopyrev et al., 2020). In these reports, the presence of actinopterygian fishes is only vaguely mentioned without taxonomic assignments other than Echnodontidae indet. Hitherto, there is no further data on the occurrence of Cretaceous bony fishes from the Orenburg region, nor from the Southern Urals as a whole.

Russian Upper Cretaceous marine deposits are widespread in the Southern Urals and numerous reports of marine vertebrates, especially marine reptiles (Bogolyubov, 1910, 1911, 1912a,b; Efimov et al., 2016) and cartilaginous fishes (Glickman and Zhelezko, 1979, 1990) exist from this region. However, these reports usually lack detailed taxonomic descriptions and illustrations are often of poor quality, if present at all. Here we present the first comprehensive evaluation of the marine vertebrates (Chondrichthyes, Actinopterygii, Reptilia) of Akkermanovka (Orenburg Oblast), provide detailed descriptions and illustrations of the recovered taxa, and discuss the biostratigraphical and palaeobiogeographical implications of this diverse assemblage.

## 2 Geological setting

**Fig. 1:**
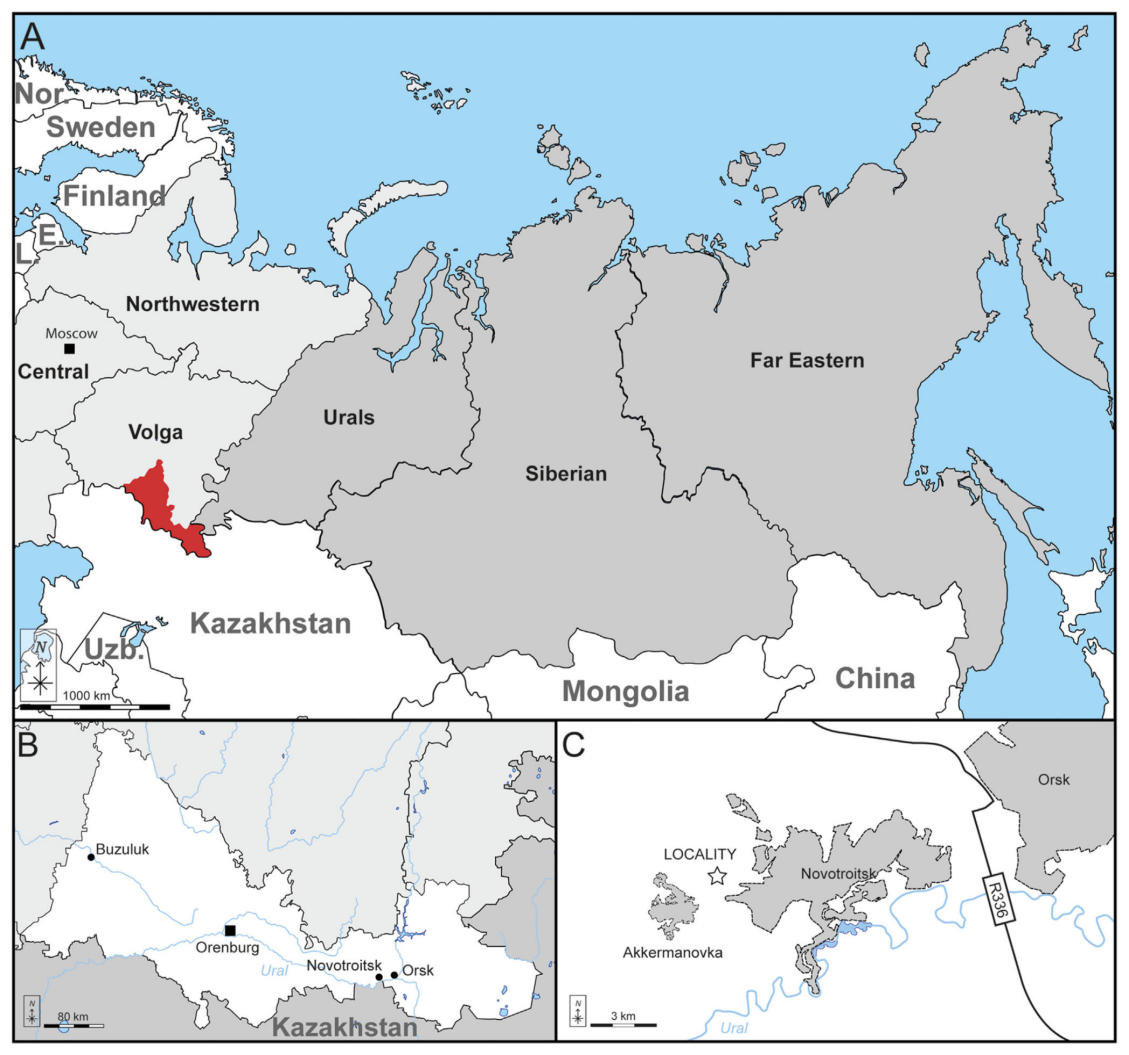
Geographic maps of Russia. (A) Map of Russia showing the location of the Orenburg Oblast (in red). European Russia is coloured in light grey; Asian Russia is coloured in dark grey. (B) Map of the Orenburg Oblast. (C) Map of the region around Akkermanovka showing the location of the study area.

The Akkermanovka locality is located in the south of the Orenburg Oblast (Southern Urals, Russia), about 2 km northeast of Akkermanovka and 15 km southwest of the city of Orsk (51.20516 N 58.24972 E; Fig. 1). Mesozoic vertebrate records, especially reptiles, have been known from Upper Cretaceous marine deposits in the Orenburg Oblast since the early 20th century (Bogolyubov, 1910, 1911, 1912a,b). These horizons have been dated to the early Campanian based on chondrichthyan remains (Efimov et al., 2016; Averianov et al., 2021b).

The Akkermanovka locality comprises two large adjacent quarries, referred to as the central quarry (“Tsentralny”), and the northern quarry. The central quarry is well known for its Carboniferous (upper Visean) deposits, which are considered an important Lagerstätte for Carboniferous invertebrates (Mychko et al., 2019). Within the Carboniferous limestones of the quarry, deep incisions and caverns secondarily filled with Cretaceous (and probably also Palaeogene) sediments are preserved (Mychko et al., 2019). Unfortunately, no reliable biostratigraphic or lithographic markers have yet been reported from these palaeodepressions (“palaeocaves”), thereby rendering their precise chronostratic ages as ambiguous (Mychko et al., 2019).

**Fig. 2:**
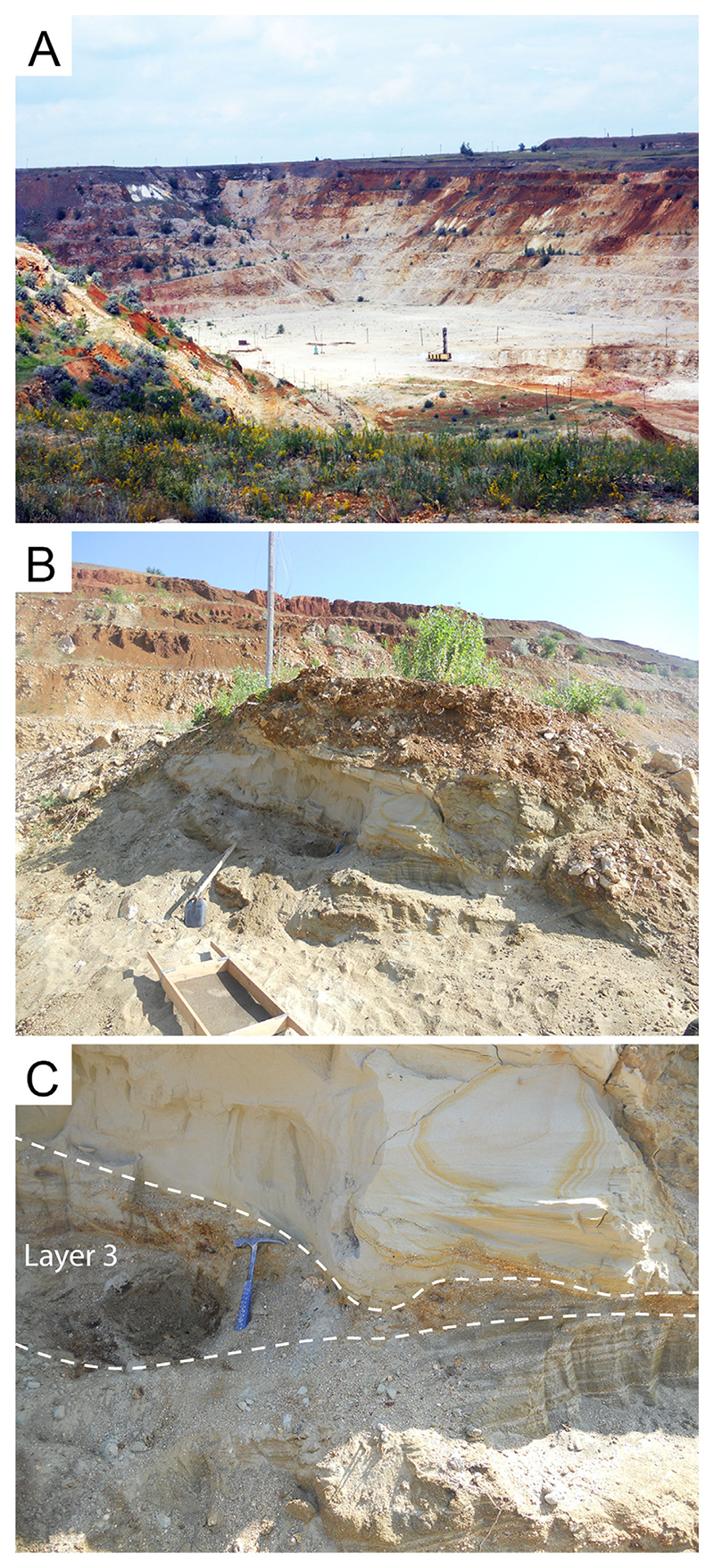
Pictures of the site and outcrop of Akkermanovka. (A) Central (“Tsentralny”) quarry near the city Akkermanovka. (B) Outcrop in the central quarry. (C) Section of the outcrop showing the fossil bearing layer (layer 3).

The new material described here was recovered from one of these “palaeocaves” in the central quarry (Fig. 2). The locality represents an area of several tens of square meters with two Upper Cretaceous outcrops of loose terrigenous sediments at the edge of the road comprising a large (up to 5 m thick) primary outcrop and a less prominent (about 2 m) secondary outcrop. The upper layer which possibly connected these two horizons has been destroyed by weathering and therefore it is unknown if these two palaeodepressions were infilled during a single event or at separate intervals.

The larger outcrop contains layers which are rich in sands and clays, as well as layers consisting of reddish to greenish clays. Fossils are extremely rare in the sands and clays but occasionally include the ammonite *Schloenbachia varians* (Sowerby, 1817), indicating a Cenomanian age (Late Cretaceous) for this layer (Kennedy, 2013). The second smaller outcrop (Figs. 2B-C) exposes a small section consisting, from bottom to top, of the following layers:

Layer 1 – lower sand layer. Fine white quartz sand (<0.1 mm) with greenish-gray fine grains with inclusions of white mica; gravel-sized grains of polycrystalline; light green, multi-sized glauconites; numerous grains of transparent uncoated and unsorted quartz; and rare lithics of siliceous rocks. The fossil remains are represented by lenticular pellets and rare fragments of reddish-coloured bones and fish scales.

Layer 2 – gray-green sand layer. White sand and gravel grains of polycrystalline quartz (2–3mm) with rarer fine grains of modified dark to light green glauconites; fine white (sometimes yellowish and pinkish) sand; and traces of white mica. Small reddish fragments of bones and fish scales are found in this layer.

Layer 3 – gravelite layer. Quartz-dominated sand with an admixture of yellowish and light green grains of modified glauconite with white and yellow coarse uncoated quartz grains, rolled with large grains of quartz or feldspar, and, rarely, angular fragments of siliceous rocks. Clasts are poorly sorted with some yellowish quartz grains measuring up to 1 cm in diameter. Marine vertebrate remains, predominately those of cartilaginous and bony fishes, are present in high abundance in this layer; comprising isolated reddish-coloured teeth, scales, and bone fragments.

Layer 4 – upper sand layer. Fine white quartz sand, with light green and yellowish grains, probably modified glauconite; quartz sand; fine modified grains of light green glauconite and, rarely, white fine mica.

All of the material described herein was collected from layer 3 (Fig. 2C).

## 3 Materials and methods

The fossil vertebrate assemblage reported herein was collected during the 2018 field season by one of us (E.V.M.; Mychko et al., 2019). Material was obtained by bulk sampling fossil-bearing sediments from bed 3 of the Akkermanovka locality, which were then screen washed using sieves with a mesh diameter of 4 mm. In total, about five tons of sediment was processed, yielding more than 1000 vertebrate remains. Of these, 470 specimens were sufficiently well preserved to be identified and are included in this study. All fossil material is stored in the collections of the Museum of the World Ocean (MMO) in Kaliningrad, Russian Federation.

Photographs of the specimens were taken with a Nikon D5200 DSLR with a mounted 18–105 mm lens. Close-up images were taken with an Eakins 37MP HDMI USB microscope camera. The photographs were edited in Adobe Photoshop CS6 (version 13.0, Adobe Systems, San Jossé, USA) for colour balance and contrast as part of the production of the figures presented herein.

## 4 Systematic palaeontology

*Remarks*. Systematics and tooth terminology mainly follow Cappetta (1987, 2012) for Euselachii, Stahl (1999) for Holocephali, Nelson et al. (2016) for Actinopterygii, O'Keefe (2001) for Sauropterygia, and Madzia and Cau (2017) for Mosasauridae. The use of open nomenclature qualifiers follows the standards proposed by Matthews (1973), Bengtson (1988), and Sigovini et al. (2016).

The Russian palaeoichthyologist Leonid S. Glickman was a renowned researcher of his field and published most of his work in Russian, thus his name was usually written in Cyrillic. Several English transliterations of his name exist throughout the literature, e.g., “Glikman”, “Glickman”, “Glückman”, or “Glyckman”, but Glickman preferred the transliteration “Glickman” as seen in his few works published in English (i.e., Averianov and Glickman, 1996; Glickman and Averianov, 1998). However, for taxonomy, he used the name “Glückman” and added this spelling to newly erected taxa names or when he was mentioning taxa that he erected earlier (see Glickman, 1964; Glickman and Zhelezko, 1979; Glickman and Averianov, 1998). In order to be consistent throughout this manuscript, the transliteration “Glückman” is used for taxonomic purposes, whereas “Glickman” is used to refer to his work, as suggested by Popov (2016).

Class **Chondrichthyes** Huxley, 1880

Subclass **Holocephali** Bonaparte, 1831

Order **Chimaeriformes** Obruchev, 1953

Family **Callorhynchidae** Garman, 1901

Genus ***Elasmodus*** Egerton, 1843

*Type species. Elasmodus hunteri* Egerton, 1843 from the lower and middle Eocene of England.

***Elasmodus*** sp.

Figs. 3A-C

*Material*. One fragmentary right mandibular tooth plate (MMO № 12260/463).

*Description*. The mandibular tooth plate is only partly preserved and is broken at its distal and labial margin. It is robust, with its anterior part being strongly bent mesially towards the symphysis, forming a long and narrow beak-like projection. The symphysis bears a large laminated tritor along the whole symphyseal edge, whereas the middle tritor on the occlusal surface of the mandibular tooth plate consists of tubular dentine. The distance between the symphysis and the middle tritor is about equal to the rostrocaudal diameter of the middle tritor. Due to its fragmentary nature, no other tritorial areas (i.e., middle outer tritors, posterior outer tritors) are preserved.

*Remarks*. The dentition in Chimaeriformes consists of a pair of lower tooth plates (mandibular plates) and two pairs of upper tooth plates: an anterior pair (vomerine plates) and a posterior pair (palatine plates). Hypermineralized tissue is present and concentrated on tritorial areas (tritors). The arrangement of these tritors, as well as the shape of the tooth plates are important diagnostic characters to distinguish between genera and even species, although intraspecific variation can be observed (Stahl, 1999; Cicimurri and Ebersole, 2014).

The genus *Elasmodus* differs from other Cretaceous chimaeroids by the presence of a long lamellar tritor along the whole cutting edge of the symphyseal margin of the mandibular tooth plate, as well as lamellar tritors on the palatine- and vomerine plate (Stahl, 1999; Averianov, 2001). It was suggested that this dental specialization in *Elasmodus* was the result of an adaptation towards scavenging on vertebrate carcasses (Averianov, 2001). According to Popov (2013), this genus comprises 16–17 valid species ranging from the Middle Jurassic to the early Oligocene, seven of which are known from the Cretaceous: *Elasmodus avirostris* Averianov in Averianov et al., 1999 from the Maastrichtian of Kazakhstan; *E. greenoughi* Agassiz, 1843 from the ?Maastrichtian of Belgium; *E. planus* Leriche, 1929 from the Maastrichtian of the Netherlands; *E. kawai* Consoli, 2006 from the K/Pg boundary of Chatham Island, Oceania; *E. khosatskyi* Averianov in Averianov et al., 1999 from the Maastrichtian of Kazakhstan; *E. rossicus* Averianov in Averianov et al., 1999 from the upper Albian of Russia; *E. sinzovi* Averianov in Averianov and Glickman, 1994 from the upper Turonian–Santonian of Russia; and *E. zharyk* Averianov in Averianov et al., 1999 from the Campanian of Kazakhstan. Due to incompleteness of the available material, it is not possible here to confidently identify the elements beyond the genus level, and is thus left in open nomenclature.

Genus ***Ischyodus*** Egerton, 1843

*Type species. Chimaera townsendi* Buckland, 1835 from the Tithonian (Upper Jurassic) of southern England.

***Ischyodus yanschini*** Averianov, 1991

Figs. 3D-I

*Material*. One fragmentary left mandibular plate (MMO № 12260/464), one fragmentary right palatine plate (MMO № 12260/465), and one almost complete left mandibular plate (MMO № 12260/466).

**Fig. 3:**
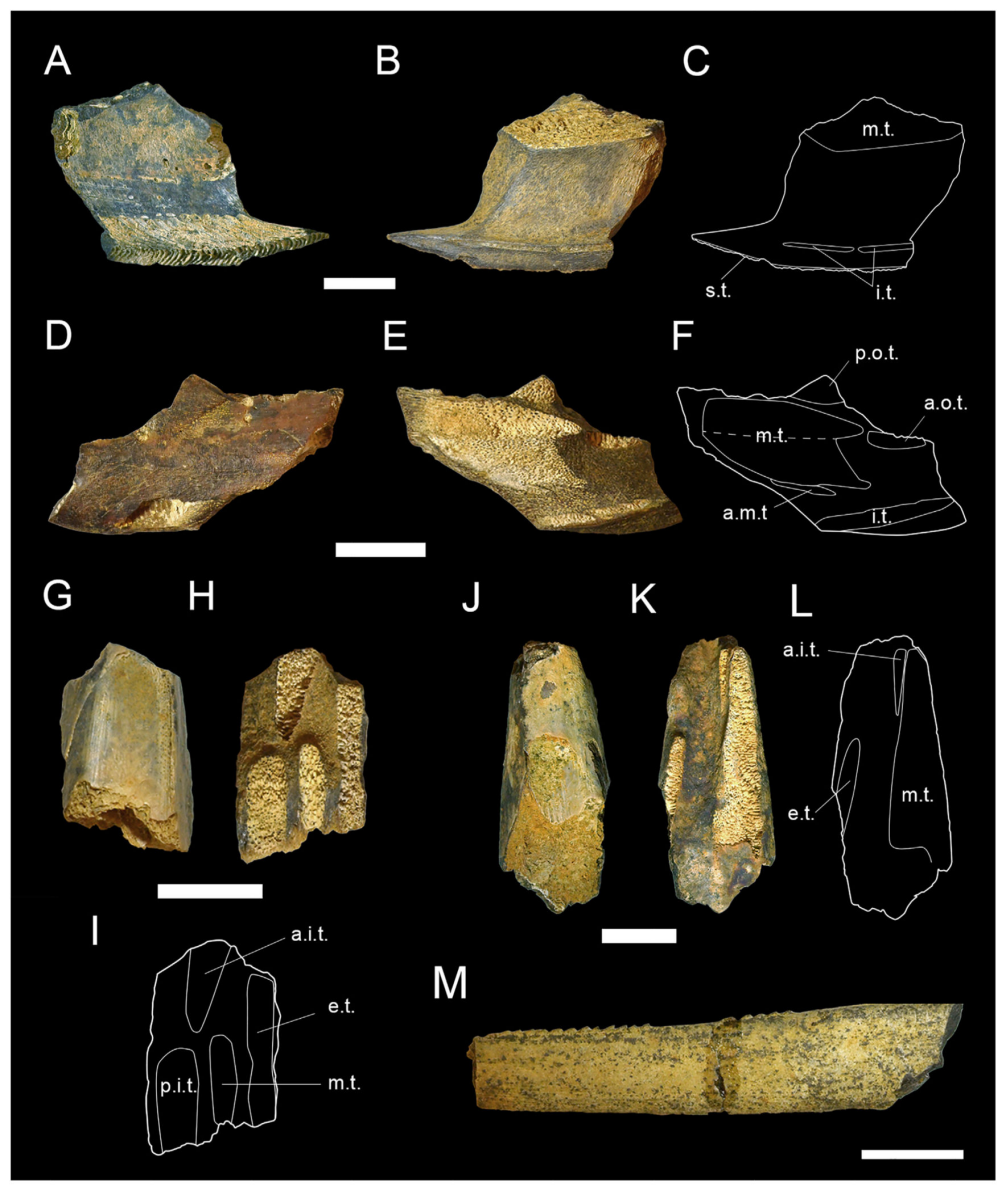
Chimaeriformes remains from the Akkermanovka locality in the Orenburg Oblast, Russia. (A-C) *Elasmodus* sp.: MMO № 12260/463, right mandibular tooth plate, (A) aboral view, (B) occlusal view, (C) interpretative drawing in occlusal view. (D-L) *Ischyodus yanschini*: (D-F) MMO № 12260/466, left mandibular tooth plate, (D) aboral view, (E) occlusal view, (F) interpretative drawing in occlusal view; (G-I) MMO № 12260/465, fragmentary left palatine plate, (G) aboral view, (H) occlusal view, (I) interpretative drawing in occlusal view; (J-M) Chimaeroidei indet.: (J-L) MMO № 12260/462, fragmentary right palatine tooth plate, (J) aboral view, (K) occlusal view, (L) interpretative drawing in occlusal view; (M) MMO № 12260/467, incomplete dorsal fin spine. *Abbreviations*: a.i.t. = anterior interior tritor; a.m.t. = accessory middle tritor; a.o.t. = anterior outer tritor; e.t. = external tritor; i.t. = internal tritor; m.t. = middle tritor; p.i.t. = posterior internal tritor; p.o.t. = posterior outer tritor; s.t. = symphyseal tritor. Scale bars = 10 mm.

*Description*. The better-preserved mandibular tooth plate (MMO № 12260/466) is lacking most of the labial margin and part of the distal and lingual margin. The anterior-most part of the mandibular plate is bent mesially, forming a short and stout beak-like projection. There are four main tritors on the occlusal surface of the tooth plate (Figs. 3E-F): two tritors along the labial margin (outer tritors), one inner tritor close to the symphyseal margin, and one large middle tritor with a small accessory middle tritor. The middle tritor is the most pronounced tritor pad, occupying most of the inner surface. It has a bifurcated anterior margin and is divided into two halves. Along the labial margin, the posterior outer tritor is situated on the distal prominence and mostly missing but was presumably large, whereas the anterior outer tritor is small and situated on the medial prominence, anterior to the middle tritor.

The second mandibular plate (MMO № 12260/464) is very fragmentary with only the anterior-most part of the plate preserved. The same four main tritorial pads as in MMO № 12260/466, including the bifurcated middle tritor, are present, although fragmentary. Additionally, a laminated tritor is situated on the anterior-most part of the symphyseal margin.

The preservation of the palatine tooth plate MMO № 12260/465 is very fragmentary, with most of the posterior end and part of the anterior end missing. Four tritorial areas are visible: two inner tritors (anterior and posterior inner tritor), one middle tritor, and one outer tritor (Figs. 3G-I).

*Remarks*. The genus *Ischyodus* has the longest fossil record among all callorhynchids, spanning from the Middle Jurassic to the Miocene (Stahl, 1999; Villalobos-Segura et al., 2023). Currently, 10 valid nominal species of this genus are known from Cretaceous strata: *Ischyodus bifurcatus* Case, 1978a from the Maastrichtian of New Jersey, USA; *I. gubkini* Nessov in Nessov et al. 1986 from the Albian of Russia; *I. incisus* Newton, 1878 from the Cenomanian of England; *I. latus* Newton, 1878 from the Albian of England; *I. lonzeensis* Leriche, 1929 from the Santonian of Belgium; *I. minor* Rogovich, 1861 from the Cenomanian of Ukraine; *I. planus* Newton, 1878 from the Albian of England; *I. rayhaasi* Hoganson and Erickson, 2005 from the Maastrichtian of North Dakota, USA; *I. thurmanni* Pictet and Campiche, 1858 from the Albian of England; and *I. yanschini* Averianov, 1991 from the Campanian of Kazakhstan. *Ischyodus* palatine plates differ from *Edaphodon* in having four tritorial pads instead of three (Cicimurri and Ebersole, 2014; Ebersole et al., 2022). Mandibular plates of *Ischyodus* are labiolingually thinner and the anterior beak is generally shorter than in *Edaphodon* and *Elasmodus*. Another characteristic is the bifid middle tritor, which is the result of fusion between the internal posterior and median tritors (Case, 1978a; Ebersole et al., 2022). This feature was originally regarded to be diagnostic for the species *I. bifurcatus* (Case, 1978a), but it is also present in several Jurassic taxa (Popov and Machalski, 2014), as well as in the Late Cretaceous species *I. rayhassi* and *I. yanschini*. Mandibular tooth plates of *I. rayhassi* can be distinguished from *I. bifurcatus* by having a more elongated lower branch of the middle tritor, which extends to the anterior outer tritor, whereas in *I. bifurcatus*, both branches are of similar length (Ebersole et al., 2022). The mandibular plates of *I. yanschini* can be distinguished from *I. bifurcatus* by having a narrower median tritor and a shorter posterior outer tritor (Averianov, 1991).

Despite the fragmentary nature of the above-described material, the arrangement of the tritorial pads is preserved in both the mandibular and palatine tooth plates, corresponding well to the type material of *I. yanschini*, which was described from the Campanian of western Kazakhstan (Averianov, 1991). To the best of our knowledge, this is the first record of this species outside of its type locality.

**Chimaeroidei** indet.

Figs. 3J-M

*Material*. One fragmentary left palatine plate (MMO № 12260/462) and one incomplete dorsal fin spine (MMO № 12260/467).

*Description*. The dorsal fin spine is fragmentary, missing the basalmost part and its apex. It is elongated, widest at the base and tapers towards the apex. In lateral view, it is slightly curved posteriorly and exhibits a faintly convex anterior face and a concave posterior face. The surface of the fin spine is smooth, but exhibits several fine parallel vertical ridges. The anterior face exhibits a keel along the midsection of the preserved spine fragment. The posterior face bears a vertical furrow medially and bears a row of denticles on the left edge of the furrow. The denticles are stout, triangular in shape, and have a basally curved apex. The basal part of the fin spine fragment is lacking any denticles. The right edge of the vertical furrow is heavily damaged without any preserved denticles.

The palatine plate is very robust and elongate. The posterior end is broken and part of the labial margin is missing. The palatine plate bears three tritor pads, one inner tritor, one median tritor, and one outer tritor. The inner tritor is the most prominent of the three and is in close proximity to the median tritor, almost fusing with it. The outer tritor is well separated from the other two.

*Remarks*. In Chondrichthyes, the cartilaginous endoskeleton is only preserved under exceptional conditions and thus the fossil record of chimaeroids mainly comprises tooth plates. Fin spines, frontal claspers, egg cases, or holomorphic specimens are rare. Cretaceous chimaeroid species were mainly erected based on isolated tooth plates, which have been used to decipher their taxonomy and phylogenetic interrelationships (e.g., Averianov, 2001; Popov, 2008; Cicimurri and Ebersole, 2015). In contrast, fin spines are rare and usually preserved isolated, not in association with tooth plates and they are not diagnostic for species (Case and Herman, 1973). Therefore, we cannot assign the dorsal fin spine to any of the two chimaeroid taxa reported here from the Akkermanovka locality, or to any other chimaeroid species.

The palatine plate bears three tritorial pads and can thus be distinguished from *Ischyodus*, which exhibits four (Cicimurri and Ebersole, 2014; Ebersole et al., 2022). *Elasmodus* is known to exhibit three tritorial pads together with a fourth, lamellar lateral tritor (Averianov, 2001; Ebersole et al., 2022). Due to the fragmentary preservation, it cannot be ruled out that such a tritor was originally present in MMO № 12260/462. However, due to the small number of comparative material from the Akkermanovka locality and the poor preservation of MMO № 12260/462, we leave it conservatively in open nomenclature.

Subclass **Elasmobranchii** Bonaparte, 1838

Cohort **Euselachii** Hay, 1902

Order **Hybodontiformes** Patterson, 1966

Family **Hybodontidae** Owen, 1846

Genus ***Meristodonoides*** Underwood and Cumbaa, 2010

*Type species. Hybodus rajkovichi* Case, 2001 from the Cenomanian (Upper Cretaceous) of Minnesota, USA.

***Meristodonoides*** sp.

Figs 4A-I

*Material*. One complete and four incomplete teeth (MMO № 12260/333 and MMO № 12260/339-342).

*Description*. The teeth are relatively large, up to 10 mm wide and 8 mm high, labio-lingually compressed and close to symmetrical in labio-lingual view, displaying a high main cusp that is slightly inclined lingually and distally. The main cusp is subcircular in cross-section and splays out towards the base of the crown. It is flanked on each side by up to three pairs of small lateral cusplets, except for one tooth, which lacks lateral cusplets completely. The cutting edge is moderately well-developed, slightly labially displaced and continuous, extending from the main cusp across all lateral cusplets. The tooth crown ornamentation consists of moderately well-developed vertical folds covering the lower parts of the labial and lingual crown faces, although they may reach up to the apex of the main cusp as well. The folds on the labial crown face become stronger towards the base of the crown. The lingual face may exhibit a horizontal ridge that extends just above the crown-root junction.

The root, which is preserved only in one tooth, is low and perforated by numerous small foramina. It slightly protrudes below the crown labially and lingually. The base of the root is flat and displays a sub-rectangular outline, forming a shallow depression that extends along the labial edge.

*Remarks*. The stratigraphic range of the genus *Meristodonoides* is recognized as spanning from the Tithonian to the Maastrichtian (Underwood, 2020; Stumpf et al., 2021a, 2022). The genus was established by Underwood and Cumbaa (2010) based on isolated teeth from the Cretaceous of North America to include four species that were originally referred to *Hybodus* Agassiz, 1837, which is generally accepted to form a polyphyletic assemblage of unrelated species characterized by very similar dental morphologies (e.g., Maisey, 1987; Rees and Underwood, 2008; Maisch and Matzke, 2016; Stumpf et al., 2021b). The *Hybodus* species that were included in *Meristodonoides* by Underwood and Cumbaa (2010) are *H. rajkovichi* Case, 2001 from the Cenomanian of Minnesota, USA; *H. butleri* Thurmond, 1971 from the Aptian or Albian of Texas, USA; *H. montanensis* Case, 1978b from the Campanian of Montana and Wyoming, USA; and *H. novojerseyensis* Case and Cappetta, 2004 from the Maastrichtian of New Jersey, USA. More recently, a new species of *Meristodonoides, M. multiplicatus* Cicimurri, Ciampaglio and Runyon, 2014 was proposed based on isolated teeth from the Santonian–Campanian of Mississippi. Cretaceous records reminiscent of *Meristodonoides* from outside of North America are restricted to one tooth from the Albian of France described as *Hybodus* sp. (Biddle, 1993) and a few teeth from the Coniacian of Ireland described as *Meristodon* sp. (Underwood and Ward, 2008) and *Meristodonoides* sp. (Guinot et al., 2013). However, the generic affinities of these teeth are here considered dubious due to their incomplete and fragmentary nature. Underwood and Cumbaa (2010) illustrated several teeth of *M. rajkovichi* with well-preserved roots and indicated a previously unrecognized intraspecific variation regarding crown ornamentation and number of cusplets. Due to the low number and poor preservation of the above-described teeth from Akkermanovka, it is difficult to unambiguously assign them to one of the known species. We therefore leave them tentatively in open nomenclature. The teeth from the Akkermanovka locality are the first record for this genus from Russia.

Family *incertae sedis*

cf. ***Polyacrodus*** sp.

Figs. 4J-N

*Material*. Five partially preserved teeth (MMO № 12260/334-338).

*Description*. The teeth are represented by incomplete crowns only. One tooth is tentatively identified as originating from an anterior tooth file, while the others are recognized as coming from tooth files of lateral positions.

The anterior tooth is slightly arched in labio-lingual view and gently curved in occlusal aspect, displaying a low but rather massive main cusp that is flanked on each side by two pairs of small lateral cusplets. Labially, the crown forms a weakly developed protuberance at the base of the main cusp. There is a moderately well-developed occlusal crest that appears to have been continuous across the cusp and cusplets. The crown is ornamented with moderate to strongly developed folds that descend from the main cusp and lateral cusplets down to the crown base. The folds may occasionally bifurcate basally.

The lateral teeth are represented by crowns that are narrow labio-lingually and wide mesio-distally, exhibiting a low profile in labio-lingual aspect. The main cusp is low and rather wide at its base. It is flanked on each side by up to five pairs of small lateral cusplets, gradually decreasing in height as they pass further away from the main cusp. Labially, there is a weak to moderately well-developed protuberance at the base of the principal cusp. The occlusal crest that extends along the entire length of the crown is weak to moderately well-developed.

Although sharing the same general morphology, the lateral teeth can be further separated into two morphotypes based on minor differences in tooth crown ornamentation. The first morphotype includes teeth that are ornamented with rather short, moderately well-developed vertical folds that descend down from the main cusp and lateral cusplets. Lingually, the crown displays a series of very short vertical folds extending along the crown shoulder.

The second morphotype, which is represented by a single specimen only, exhibits an ornamentation that consists of densely arranged vertical folds that descend not only from the cusp and cusplets but also from the occlusal crest down to the base of the crown. In some cases, the vertical folds bifurcate basally towards the crown shoulder.

*Remarks*. The above-described teeth from the Akkermanovka locality resemble those of *Acrodus illingworthi* Dixon, 1850 from the Cenomanian of England, which represents a poorly defined species that has repeatedly been placed in the genus *Polyacrodus* Jaekel, 1889 (e.g., Dorka, 2003; Ward, 2010; Cappetta, 2012; Guinot et al., 2013). Fossil teeth of similar morphology have also been described from the Turonian of the Czech Republic as *Polyacrodus polydictios* (Reuss, 1846) by Wiese et al. (2004) and from the Campanian of Sweden as *Polyacrodus siversoni* by Rees (1999). However, controversial issues concerning the taxonomic content and validity of *Polyacrodus* still prevail (Rees and Underwood, 2002, 2008; Rees, 2008; Cappetta, 2012), pending further study. For this reason, and given its limited and fragmentary nature, the Akkermanovka material is here tentatively referred to as cf. *Polyacrodus* sp.

Subcohort **Neoselachii** Compagno, 1977

Order **Synechodontiformes** Duffin and Ward, 1993

Family **Palaeospinacidae** Regan, 1906

Genus ***Synechodus*** Woodward, 1888

*Type species. Hybodus dubrisiensis* Mackie, 1863 from the Cenomanian (Upper Cretaceous) of Dover, England.

***Synechodus*** sp.

Figs. 5A-F

*Material*. Five complete teeth (MMO № 12260/327-331).

*Description*. The teeth are up to 9 mm high and 11 mm wide. The crown bears a large main cusp that is flanked by high lateral shoulders. Both shoulders are oblique to the main cusp and bear three to four lateral cusplets each, which gradually decrease in height from the main cusp towards the marginal edges. Lateral cusplets are well separated from each other by concave notches, which terminate far from the root-crown boundary. The main cusp is strongly lingually inclined and points distally towards the jaw commissure. The labial and lingual face of the crown and the cusplets are convex. The main cusp exhibits a medial notch at the base of the labial face. The labial face of the crown is ornamented with weak flexuous folds extending from the base of the crown to approximately ½ of the main cusp. Medially, the labial face of the main cusp is smooth and does not exhibit any ornamentation. There are also weak folds at the base of the shoulders, which turn into pronounced folds on the lateral cusplets, reaching their apex. Some of the specimens have a completely smooth labial surface as a result of wear (MMO № 12260/327 and 328). The lingual side of the main cusp bears clear flexuous striae reaching half the height of the main cusp and extending up to the apex of the lateral cusplets. The crown overhangs the root labially by a prominent bulge that almost reaches the basal face of the root. The root is pseudopolyaulacorhize. The root is lingually directed with several distinct nutritive grooves. Several laminae are located at the base of the labial side of the root.

*Remarks*. Teeth of *Synechodus* can be distinguished from the morphologically similar teeth of *Paraorthacodus* by their low root, a labial bulge overhanging the root, and their rather small lateral cusplets, which are broadly united basally [separated from one another and the main cusp in *Paraorthacodus*; see Duffin and Ward (1993)]. Isolated teeth assigned to this genus cover a wide stratigraphic range, from the Early Permian to the Danian, Palaeocene (Ivanov, 2005; Cappetta, 2012). Following Siversson et al. (2016) and Cappetta et al. (2021), 27 nominal species can be regarded as valid, 13 of which occurred in the Late Cretaceous: *Synechodus bronnii* (Reuss, 1846) from the Cenomanian of the Czech Republic; *S. dereki* Cappetta, Morrison and Adnet, 2019 from the Campanian of the Hornby Island, Canada; *S. dispar* (Reuss, 1846) from the Cenomanian of the Czech Republic; *S. dubrisiensis* (Mackie, 1863) from the Cenomanian of England; *S. filipi* Siversson, Cook, Cederström and Ryan, 2016 from the lower Campanian of Sweden; *S. kessleri* (Rogovich, 1861) from the Cenomanian of Ukraine; *S. lerichei* Herman, 1977 from the Campanian and Maastrichtian of Belgium; *S. nitidus* Woodward, 1911 from the Cenomanian of England; *S. polyptychus* (Reuss, 1846) from the Cenomanian of the Czech Republic; *S. subulatus* (Rogovich, 1861) from the Cenomanian of Ukraine; *S. tuberculatus* (Rogovich, 1861) from the ?Cenomanian of Ukraine; *S. turneri* Case, 1987 from the upper Campanian of Wyoming, USA; and *S. validus* Chapman, 1918 from the Upper Cretaceous of New Zealand. It should be noted though that many of these taxa described in the 19th and 20th century have been poorly illustrated and the type material has been lost since then, making detailed comparisons difficult. Furthermore, it appears that at least some of the morphotypes described as different nominal species might be the product of heterodonty. For example, Guinot et al. (2013) pointed out that *S. dubriesiensis, S. nitidus*, and *S. tenuis* Woodward, 1889 are always found associated in published faunal assemblages and inferred that teeth from the Cenomanian with smooth crown faces that were assigned to *S. nitidus* and *S. tenuis* might in fact represent adult female teeth of *S. dubriesiensis*. Although a few articulated dentitions are known, it has been impossible to date to prove the existence of ontogenetic or sexual dimorphism in *Synechodus*. The teeth described here from Akkermanovka cannot be unambiguously assigned to one of the known morphotypes. It should be noted though, that they closely resemble teeth figured in Cappetta (2012; fig. 306), which were assigned to *Synechodus* sp. And are from the upper Santonian of Kazakhstan. However, due to the small sample size and the preservation of the teeth from the Akkermanovka locality, it is difficult to compare the teeth of both localities properly.

Family **Paraorthacodontidae** Klug, 2009

Genus ***Paraorthacodus*** Glückman, 1957

*Type species*: *Sphenodus recurvus* Trautschold, 1877 from the Cenomanian (Upper Cretaceous) of the Volga area, Russia.

***Paraorthacodus*** cf. ***andersoni*** (Case, 1978b)

Figs. 5G-N

*Material*. Twenty-four mostly complete teeth (MMO № 12260/154-177): seven anterior teeth (MMO № 12260/154, MMO № 12260/160, MMO № 12260/163, MMO № 12260/166, MMO № 12260/168, MMO № 12260/169, MMO № 12260/171), 16 lateral teeth (MMO № 12260/155-159, MMO № 12260/161-162, MMO № 12260/164-165, MMO № 12260/167, MMO № 12260/170, MMO № 12260/172-176), and one pathological tooth (MMO № 12260/177).

*Description*. The teeth have a maximum height of up to 13 mm and a width of up to 15 mm. In anterior teeth, the main cusp is flanked by up to two lateral cusplets, while lateral teeth have up to three lateral cusplets. Main cusp and lateral cusplets are upright and slender, with a slightly expanded base. The lateral cusplets are well separated from the main cusp, only joined to it by a thin strip of enameloid. The pair of lateral cusplets next to the main cusp are twice as high as the more laterally situated cusplets in anterior teeth. This ratio is less pronounced in lateral teeth and sometimes the cusplets next to the main cusp have even the same height as the more lateral ones. The main cusp is erect In anterior teeth, while it is distally inclined towards the commissure in lateral teeth. The main cusp is lingually inclined. The labial face of the main cusp is slightly convex, whereas its lingual face is strongly convex. The labial face of the crown does not overhang the crown-root junction. Vertical ridges or striae originate from the base of the crown. On the lingual side of the main cusp, they are thin and reach ⅓ of the height of the cusp, while in profile view, they become more distinct and reach ⅕ of the crown height. Striae are also present on the labial face, terminating around ⅕ to ½ of the way up to the apex, and on the lateral cusplets, where they can extend up to the apex of the cusplet. The root is pseudopolyaulacorhize. In basal view, the root is elliptical with a concave labial margin and a convex lingual margin. Several foramina are arranged in a row on the root surface. In the median part, the root usually shows an alternation of five laminae and grooves (up to eight).

*Remarks*. Ambiguity is shrouding the systematic position of this genus, with some authors regarding it to belong to the order Hexanchiformes (Cappetta, 2012; Sokolskyi and Guinot, 2021), while others see its affiliation closer to an ancient neoselachian stem group, the Synechodontiformes (Duffin and Ward, 1993; Klug et al., 2009; Adolfssen and Ward, 2014). As for now, the only comprehensive phylogenetic analysis that has been conducted on this genus supports its affiliation to the order Synechodontiformes (Klug, 2010) and we, therefore, tentatively follow this hypothesis. The genus is known from the Early Jurassic to the Palaeogene (Klug et al., 2009) and five taxa from the Cretaceous are currently considered to be valid: *Paraorthacodus andersoni* (Case, 1978b) from the Campanian of Montana, USA; *P. antarcticus* Klug, Kriwet, Lirio and Nuñez, 2008 from the lower Campanian of James Ross Island, Antarctica; *P. conicus* (Davis, 1890) from the lower Campanian of Skåne, Sweden; *P. recurvus* (Trautschold, 1877) from the Cenomanian of the Volga region, Russia; and *P. rossi* Cappetta, Morrison and Adnet, 2019 from the Campanian of Hornby Island, Canada. Although smaller in size, the morphology of the here presented teeth corresponds well with those of *P. andersoni* described from the Upper Cretaceous of Sweden by Siverson (1992a) and Adolfssen and Ward (2014). It should be noted that the teeth described from Sweden show differences in morphology and ornamentation to the holotype specimen illustrated in Case (1978b). Siverson (1992a), however, did also illustrate specimens from the type locality which do conform with the morphology and ornamentation seen in the Swedish material and the material from Akkermanovka described here. Nonetheless, we agree with Adolfssen and Ward (2014) that a thorough review of this species is needed in order to resolve if the observed differences is merely the product of intraspecific variation or representative of two species.

***Paraorthacodus*** sp.

Figs. 4O-P

*Material*. Six heavily abraded teeth (MMO № 12260/178-183).

*Description*. The teeth are up to 18 mm high and 18 mm wide. The main cusp is stout and rather short and is flanked by three pairs of rather low lateral cusplets. A smooth cutting edge extends across the main cusp and sometimes also across the lateral cusplets. The main cusp is lingually inclined and straight in anterior teeth, whereas it is distally inclined in lateral teeth. The lingual face of the main cusp is strongly convex, while the labial face of the cusp is only slightly convex. Thin striae (from straight to curved) are present laterally at the base of the lingual face of the main cusp (and sometimes lateral cusplets), while the mesial part remains smooth. The striae observed in the cusplets reach up to ½ of the height of the cusplet. The labial crown face is smooth. A prominent neck is present on the lingual face (up to ⅕ of the height of the main cusp) of the main cusp (and sometimes cusplets). The neck is rounded and sometimes triangular in the mesial part. The root is pseudopolyaulacorhize. The root surface has several foramina lingually which are arranged in a row, where the mesially situated foramina represent elongated grooves. In basal view the root is rectangular or with a convex lingual margin.

*Remarks*. The teeth assigned here to *Paraorthacodus* sp. are morphologically similar to the teeth we identified as *Paraorthacodus* cf. *andersoni* but differ in being stouter, bearing lower lateral cusplets, and the lack of striations on the labial face of the main crown. The combination of characters described above is also not known for any of the other Cretaceous *Paraorthacodus* species. Skeletal material from the Jurassic shows that *Paraorthacodus* exhibited a gradual monognathic heterodonty (Duffin, 1993; Klug et al., 2009), but this does not explain the differences observed in the two tooth morphotypes from the Akkermanovka locality. Gynandric heterodonty (i.e., sexual dimorphism in teeth) is a widespread phenomenon in elasmobranch fishes (Kajiura and Tricas, 1996; Underwood et al., 2015; Berio et al., 2020). However, the presence of such a tooth dimorphism in the genus *Paraorthacodus* needs to be confirmed yet. Due to the small number of teeth and their poor preservation, we conservatively left this second morphotype of *Paraorthacodus* from the Akkermanovka locality tentatively in open nomenclature.

Superorder **Galeomorphi** Compagno, 1973

Order **Orectolobiformes** Applegate, 1972

Family **Orectolobidae** Jordan and Fowler, 1903

Genus ***Cederstroemia*** Siverson, 1995

*Type species. Cederstroemia triangulata* Siverson, 1995 from the Campanian (Upper Cretaceous) of Blaine County, Montana, USA.

***Cederstroemia nilsi*** Siverson, 1995

Figs. 6A-M

*Material*. Eleven mostly complete teeth (MMO № 12260/64-74).

*Description*. The teeth are relatively small in size with a maximum height of up to 6 mm and a width of up to 11 mm. Anterior teeth are symmetrical with a bulky cusp, while lateral teeth are slightly asymmetrical with a distally inclined cusp. The crown of the tooth is directed lingually. It consists of a triangular main cusp that is flanked by elongated mesial and distal shoulders. The mesial and distal shoulders are up to one-third the height of the main cusp and are slightly perpendicular or oblique to the main cusp. The cutting edge extends across the lateral shoulders and the main cusp. Both the labial and lingual faces of the crown are convex to varying degrees, with the lingual face always being more convex than the labial face. Both crown faces are smooth without any ornamentation. The apron is rectangular in shape and elongated basally reaching or overhanging the baseline of the root in labial view. There is a prominent bulge at the base of the lingual side of the main cusp. The root is hemiaulacorhize in most teeth, but can be partially holaulacorhize. In basal view, the root has a sub-rectangular outline. The basal surface is flat to faintly concave and a large foramen is centrally located, with several smaller foramina arranged in a row or scattered randomly. A row of small foramina is situated on the lingual face along the root-crown boundary. Additionally, several randomly distributed smaller accessory foramina are present and a large foramen may be open on the lingual protrusion. The labial root face is relatively low in each specimen and bears a series of labial foramina that are organized in a row. In some teeth, several additional randomly situated foramina can be present.

*Remarks. Cederstroemia* was originally included in another orectolobiform shark genus, *Cretorectolobus*, but can be distinguished from it by its lack of cuspidate shoulders and the lower and bulkier main cusp. Teeth of both genera are morphologically similar to those of *Squatina* (Squatiniformes) but differ from it by having higher and more oblique lateral shoulders, much lower cusps, and a strongly oblique root profile (Siverson, 1996; Ebersole et al., 2022). Additionally, the root can be holaulacorhize, which is never the case in *Squatina*. Five nominal species have been described for this genus and are currently regarded to be valid: *Cederstroemia havreensis* (Herman, 1977) from the Campanian of Belgium; *C. nilsi* Siverson, 1995 from the Campanian of Sweden; *C. siverssoni* Guinot, Underwood, Cappetta and Ward, 2013 from the middle Turonian of France; *C. triangulata* Siverson, 1995 from the Campanian of Montana, USA; and *C. ziaensis* Bourdon, Wright, Lucas, Spielmann and Pence, 2011 from the Santonian of New Mexico, USA.

The teeth described here closely resemble the type material of *C. havreensis* from the upper Campanian of Belgium and *C. nilsi* from the lower Campanian of Sweden. Siverson (1995) described that teeth of *C. havreensis* were larger and wider than in *C. nilsi*, and had a labio-lingually very elongated central foramen (“intermediate between the hemiaulacorhizous and holaulacorhizous stage”), whereas they were always circular in *C. nilsi*. Teeth from the Akkermanovka locality usually have a circular central foramen, but some teeth tend to exhibit a holaulacorhize root. This variation of the root vascularization has also been described for *C. triangulata* and thus seems to be a poor character to distinguish species. Besides slight differences in size, *C. havreensis* mainly differs from *C. nilsi* in exhibiting a labial, rarely also lingual, ornamentation on the tooth crown, whereas *C. nilsi* is lacking any ornamentation on the crown (Guinot et al., 2013). The subtle differences between both species led Guinot et al. (2013) to suggest that both species might be conspecific. Teeth of *Cederstroemia* from the Akkermanovka locality lack any ornamentation and are thus more similar to *C. nilsi*. Therefore, we tentatively assign the material from the Akkermanovka locality to *C. nilsi* until further research supports the conspecificity of both species.

Order **Lamniformes** Berg, 1937

Family **Archaeolamnidae** Underwood and Cumbaa, 2010

Genus ***Archaeolamna*** Siverson, 1992b

*Type species. Odontaspis kopingensis* Davis, 1890 from the “Köpinge sandstone” (Campanian, Upper Cretaceous) of Kőping, Sweden.

***Archaeolamna*** ex gr. ***kopingensis*** (Davis, 1890)

Figs. 7A-H

*Material*. Fifty-five teeth (MMO № 12260/9-63): ten anterior teeth (MMO № 12260/10, MMO № 12260/19, MMO № 12260/27-29, MMO № 12260/41, MMO № 12260/43, MMO № 12260/49, MMO № 12260/51, MMO № 12260/53), twelve antero-lateral teeth (MMO № 12260/12, MMO № 12260/13, MMO № 12260/22-23, MMO № 12260/26, MMO № 12260/40, MMO № 12260/44, MMO № 12260/46, MMO № 12260/58, MMO № 12260/60, MMO № 12260/62-63), and 33 lateral teeth (MMO № 12260/9, MMO № 12260/11, MMO № 12260/14-18, MMO № 12260/20, MMO № 12260/21, MMO № 12260/24-25, MMO № 12260/30-39, MMO № 12260/42, MMO № 12260/45, MMO № 12260/47-48, MMO № 12260/50, MMO № 12260/51-52, MMO № 12260/55-57, MMO № 12260/59, MMO № 12260/61).

*Description*. The teeth reach a height of up to 23 mm and a width of up to 18 mm. The main cusp is broad based and triangular. It is almost straight in anterior teeth and distally inclined towards the commissure in lateral teeth. The lingual face of the main cusp is strongly convex while the labial face is weakly convex or flat. The labial side of the main cusp overhangs the root in some teeth. Some teeth show a medial depression at the base of the labial face. The teeth have one pair of subtriangular, well-defined divergent lateral cusplets that are separated from the main cusp by a notch. The cutting edges extend across the main cusp and lateral cusplets. Both the labial and lingual face of the main cusp and cusplets are smooth. Sometimes the main cusp has folds at the base of the labial side, this feature is especially well-defined in the posterior teeth. A prominent neck characterizes the base of the lingual face of the crown (both the main cusp and the lateral cusplets). The root is robust, bilobate and bears a pronounced lingual protuberance, which usually bears one or more nutritive foramina. The root lobes are well developed, divergent, and basally rounded. The interlobe area is deep and U-shaped.

In general, lateral teeth are mesio-distally wider and have a lower main cusp and a shallower interlobe area compared to anterior teeth.

*Remarks*. The alpha taxonomy of the genus *Archaeolamna* is currently regarded ambiguous. Several *Archaeolamna* species and subspecies have been described to date: *Archaeolamna aduncata* (Zhelezko, 1990); *A. aduncata suberecta* (Zhelezko, 1990) *A. arcuata orica* (Zhelezko, 1990); *A. haigi* Siverson, 1996; *A. kopingensis* (Davis, 1890); *A. kopingensis judithensis* Siverson, 1992b; *A. kopingensis kopingensis* Siverson, 1992b; and *A. striata* (Rogovich, 1861). The three taxa erected by Zhelezko (1990) were all derived from Santonian deposits in Kazakhstan, but their validity was questioned by Ebersole et al. (2022) who stated that they all fall within the *A. kopingensis* morphology. Traditionally, most records of *Archaeolamna* are referred to *A. kopingensis*, which now is considered to have had a cosmopolitan distribution and a stratigraphic ranged from the Albian to the Maastrichtian (113–66 mya; see Guinot et al., 2013 and references therein). It must be noted though that a variety of different tooth morphologies have been assigned to this species that apparently exhibit stratigraphical and spatial variations and it thus was suggested that *A. kopingensis* represents a group of closely related taxa rather than a single species (Underwood and Cumbaa, 2010). The size and morphology of the specimens from the Akkermanovka locality fall within this *A. kopingensis* complex and deviate from other species (i.e., *A. haigi* and *A. striata*) by their overall robust morphology. Given the taxonomic uncertainties surrounding this alleged species complex, we provisionally refer to the specimens described here as *Archaeolamna* ex gr. *kopingensis*.

Family **Otodontidae** Glückman, 1964

Genus ***Cretalamna*** Glückman, 1958

*Type species. Otodus appendiculatus* Agassiz, 1843 from the Turonian (Upper Cretaceous) of Lewes, England.

***Cretalamna sarcoportheta*** Siversson, Lindgren, Newbrey, Cederström and Cook, 2015

Figs. 6I-P

*Material*. Twenty mostly complete lateral teeth (MMO № 12260/75-94).

*Description*. The teeth reach a height of approximately 15 mm and a width of up to 18 mm. The main cusp is sub-triangular in shape with a broad base and is straight in profile view. It is slightly inclined towards the commissure in antero-lateral teeth but is strongly curved distally in teeth situated more posteriorly. A pair of lateral cusplets is flanking the main cusp. They are large, more or less divergent, and are also sub-triangular in shape. In some of the teeth, one of the two cusplets (usually the distal cusplet) have a bicuspid outline with a shallow notch splitting the apex. The lateral cusplets are separated from the main cusp by a notch. The cutting edge is smooth and continues between the main cusp and the lateral cusplets. The labial side of the main cusp is flat, whereas the lingual side is convex. The labial and lingual side of the lateral cusplets are both convex. In some teeth, the labial side of the crown overhangs the root. The roots are large, wide, and the lobes are well separated by a shallow interlobe area. The roots are asymmetrical, with the mesial lobe being more expanded compared to the distal lobe. The lobes of most teeth are angular, whereas in some teeth the distal lobe is more rounded, while the mesial lobe is pointed. The root is labio-lingually flattened with a more or less developed lingual protuberance bearing a small foramen. On the labial side, the root has a row of mesio-distally distributed foramina close to the root-crown boundary.

*Remarks*. Previously, most *Cretalamna* teeth were assigned to the type species *C. appendiculata*, which has resulted in reports of this species ranging from the Albian to the Ypresian (50 Ma). Siversson et al. (2015) reviewed this taxon and came to the conclusion that *C. appendiculata* was a species complex, which he subsequently subdivided into several species: *Cretalamna appendiculata* (Agassiz, 1843) *sensu stricto* from the Turonian of England; *C. borealis* (Priem, 1897) from the Campanian of Sweden; *C. catoxodon* Siversson, Lindgren, Newbrey, Cederström and Cook, 2015 from the Cenomanian of Western Australia; *C. deschutteri* Siversson, Lindgren, Newbrey, Cederström and Cook, 2015 from the Turonian of France; *C. ewelli* Siversson, Lindgren, Newbrey, Cederström and Cook, 2015 from the Coniacian of Kansas, USA; *C. gertericorum* Siversson, Lindgren, Newbrey, Cederström and Cook, 2015 from the Turonian of France; *C. hattini* Siversson, Lindgren, Newbrey, Cederström and Cook, 2015 from the Santonian–Campanian of Kansas, USA; and *C. sarcoportheta* Siversson, Lindgren, Newbrey, Cederström and Cook, 2015 from the Campanian of Sweden. Since this taxonomic revision, one additional species has been erected from the Santonian–Campanian of Alabama, USA: *C. bryanti* Ebersole and Ehret, 2018.

The specimens described here are closely resembling *C. sarcoportheta* and can be distinguished from the coeval *C. borealis* by its flat labial face of the main cusp (convex in *C. borealis*), the wide crown, the expanded mesial root lobe, and the less divergent lateral cusplets. *Cretalamna sarcoportheta* has been identified from Campanian deposits of Belgium, France, Sweden, and possibly Texas, USA (Siversson et al., 2015). Siversson et al. (2015) also pointed out the close resemblance between *C. appendiculata*, which they regard to be restricted to the Turonian of England, and the Campanian *C. sarcoportheta* and grouped them together within the “*C. appendiculata* group”. *Cretalamna appendiculata* has been described from Campanian deposits in Russia (Averianov and Popov, 2014; Grigoriev et al., 2015) but, following the taxonomy proposed by Siversson et al. (2015), might in fact represent *C. sarcoportheta* instead. Ebersole et al. (2022) reported the occurrence of *Cretalamna* cf. *borealis* and *C*. cf. *sarcoportheta* in Campanian deposits of Russia (Saratov Oblast) based on poorly preserved material. The teeth from the Akkermanovka locality are here assigned to *C. sarcoportheta*, supporting its presence in Russia and suggesting a circumglobal distribution for this species.

Family **Anacoracidae** Casier, 1947

Genus ***Squalicorax*** Whitley, 1939

*Type species. Corax pristodontus* Agassiz, 1835 from the Maastrichtian (Upper Cretaceous) of Maastricht, the Netherlands.

***Squalicorax kaupi*** (Agassiz, 1843)

Figs. 8A-L

*Material*. One hundred and nine mostly complete teeth (MMO № 12260/218-326).

*Description*. The teeth are small to moderately sized, with a maximum crown height and width of up to 15 mm. Anterior teeth are slightly higher than wide, whereas more lateral and posterior teeth are wider than high. The crown is straight and points towards the rear, with the degree of inclination increasing from anterior to posterior tooth files. The mesial cutting edge of the crown is moderately to strongly convex. The distal cutting edge is either straight and erect, or slightly convex. It forms an oblique to acute angle with the distal heel and a notch separating both can be present. The distal heel is straight to convex. The cutting edges are coarsely serrated, with evenly distributed serrations, albeit the serrations can be partly reduced or lost due to wear. The labial face of the crown is flat, whereas the lingual face is convex. Both crown faces are smooth and lack any ornamentation. A well-marked neck separates the crown from the root lingually. The root is lingually high, labio-lingually flattened and bears two lobes that are separated by an U-shaped interlobe area. The root lobes are rather short, divergent, and have rounded extremities. The lingual protuberance is hardly noticeable and lacks a nutritive grove.

*Remarks*. The genus *Squalicorax* is a geographically and geologically widespread genus that can be found in Albian to upper Maastrichtian deposits worldwide (Cappetta, 2012). Traditionally, *Squalicorax* was regarded to represent an evolutionary line (*S. falcatus* - *S. kaupi* - *S. pristodontus*), within which teeth become larger, the cusp becomes more erect, the distal heel gradually becomes smaller, and the notch separating it from the distal blade of the main cusp disappears (Cappetta, 1987, 2012). In the last few decades, however, it became apparent that these species did not simply replace each other but had overlapping stratigraphic ranges and the diversity of the genus was much more complex, suggesting a cladogenetic evolution instead of a simple evolutionary transition from one species into another, i.e., anagenesis (Shimada and Cicimurri, 2006; Siverson et al., 2007). As of yet, more than 50 nominal species have been described, albeit many of them are poorly described and inadequately illustrated (Siverson et al., 2007; Cappetta et al., 2014). The size and morphology of the teeth described from the Akkermanovka locality correspond well with *Squalicorax kaupi*. They differ from the Late Cretaceous taxa *S. bassanii* (Gemmellaro, 1920) and *S. yangaensis* (Dartevelle and Casier, 1943) in having a more broadly convex mesial cutting edge that lacks a concavity or distinctive notch near the crown base. Glickman (1980) described the upper Santonian species *S. obruchevi* from the Tyk-Butak River region of Aktjubinsk, western Kazakhstan, and the Campanian species *S. praeyangaensis* from Alymtau Mountain, southern Kazakhstan. Unfortunately, the descriptions do not allow to distinguish them from *S. kaupi*, in fact, it is mentioned that both species look very similar to other *Squalicorax* species. The illustrations provided are line drawings that do not allow detection of any meaningful diagnostic characters. In the absence of the type material, we regard these species as *nomina dubia*.

Another species that closely resembles *Squalicorax kaupi* is *S. lindstromi* (Davis, 1890) from the Campanian of Sweden. Schubert et al. (2017) distinguished between both species by the presence of a marked notch separating the distal heel from the distal cutting edge (notch marked in *S. kaupi*, while in *S. lindstromi*, the heel merges with the cutting edge). In the original description of Davis (1890) however, a more or less deep indent between the distal heel and the distal cutting edge is described and illustrated. Moreover, neither the description, nor the illustration indicate any differences between *S. lindstromi* and *S. kaupi*. In the absence of clear species-specific characters, they must be regarded as synonyms in accordance with the “Principle of Typification” of the International Code of Zoological Nomenclature (ICZN 1999, art. 61). Therefore, we follow previous studies in regarding *S. lindstromi* a junior synonym of *S. kaupi* (Siverson, 1992b; Ebersole et al., 2022).

Family **Pseudocoracidae** Cappetta, 2012

Genus ***Pseudocorax*** Priem, 1897

*Type species. Corax affinis* Münster in Agassiz, 1843 from the Maastrichtian (Upper Cretaceous) of Maastricht, the Netherlands.

***Pseudocorax laevis*** (Leriche, 1906)

Figs. 9A-B

*Material*. One complete lateral tooth (MMO № 12260/184).

*Description*. Specimen MMO № 12260/184 is 7 mm high and wide. The main cusp is triangular and distally inclined. It is straight, with only the apex of the crown being slightly bent lingually. The mesial edge of the main cusp is slightly convex, whereas the distal edge is convex towards the apex and concave near the base. The main cusp is flanked by two oblique lateral heels. The distal heel is rounded and separated from the distal cutting edge by a distinct notch, whereas the mesial heel is low and merges with the main cusp. The cutting edge is smooth and continuous across the crown. The lingual face of the crown is convex, whereas the labial face is flat. Both the lingual and labial side of the crown are smooth without ornamentation. The crown overhangs the root labially and is separated from it by a neck lingually. The root lobes are divergent and asymmetrical, separated by a shallow and U-shaped interlobe area. A nutritive groove is present on the lingual protuberance of the root. The labial side of the root is flat and several small foramina are arranged below the crown.

*Remarks*. The genus *Pseudocorax* has been reported from Upper Cretaceous localities worldwide, spanning from the Cenomanian to the Maastrichtian (Jambura et al., 2021). Although controversies are surrounding the validity of some of the species (see Jambura et al., 2021), six species are currently considered valid: *Pseudocorax affinis* (Münster in Agassiz, 1843) from the Maastrichtian of Maastricht, the Netherlands; *P. duchaussoisi* Guinot, Underwood, Cappetta and Ward, 2013 from the Turonian of Justine-Herbigny, France; *P. granti* Cappetta and Case, 1975a from the Campanian of Texas, USA; *P. heteromorphus* (Reuss, 1845) from the Turonian of Bohemia, Czech Republic; *P. kindlimanni* Jambura, Stumpf and Kriwet, 2021 from the Cenomanian of Hakel, Lebanon; and *P. laevis* (Leriche, 1906) from the Campanian of the Paris basin and northern France (no type locality specified). Morphology and size of specimen MMO № 12260/184 corresponds well with *P. laevis* and can be distinguished from other species by its smooth cutting edge (serrated in *P. affinis*), less developed lateral heels (very prominent in *P. kindlimanni*) and its rather robust morphology (more gracile in *P. duchaussoisi, P. granti*, and *P. heteromorphus*). It was previously suggested that the diagnostic characters used to distinguish between *P. granti* and *P. laevis* were weakly founded and that both species could be regarded as conspecific (Hamm and Shimada, 2007). Quantitative methods like geometric morphometrics and traditional morphometrics have recently been applied to explore intra- and interspecific variations in shark teeth and to test species validity (Marramà and Kriwet, 2017; Türtscher et al., 2021, 2022). A similar approach could shed light on the validity of *P. granti*, which is tentatively considered a separate species here.

Family **Cretoxyrhinidae** Glückman, 1958

Genus ***Cretoxyrhina*** Glückman, 1958

*Type species. Oxyrhina mantelli* Agassiz, 1835 from the lower Cenomanian to lower Coniacian (Upper Cretaceous) of the Lewes area, East Sussex, England.

***Cretoxyrhina mantelli*** (Agassiz, 1835)

Figs. 9C-G

*Material*. One upper lateral tooth (MMO № 12260/95) and one lower anterior (?) tooth (MMO № 12260/96).

*Description*. Specimen MMO № 12260/95 measures 23 mm in width and 22 mm in height. The tooth is slightly broken, with the apex and part of the distal cutting edge being missing. Specimen MMO № 12260/96 is well preserved and measures 13 mm in width and 23 mm in height. The main cusp of both teeth is triangular and distally inclined. In profile view, the main cusps are straight. The main cusp of MMO № 12260/95 is flanked by two oblique heels. The mesial heel smoothly descends into the main cusp, whereas the distal heel is more detached but is not separated from the main cusp by a notch. The cutting edge is smooth and continuous across the main cusp and the mesial and distal heels. The lingual face of the crown is convex in both teeth, whereas the labial face is only slightly convex, almost flat. Both lingual and labial sides of the crown are smooth. The labial crown face slightly overhangs the root. On the lingual side, a well-developed neck separates the crown from the root. The root is slightly asymmetric in MMO № 12260/95, with a shorter but broader distal lobe and a narrower but much longer mesial lobe. In MMO № 12260/96, this asymmetry is much more pronounced, with the mesial lobe being more than three times the size of the distal root lobe. In both specimens, the root lobes are well separated from each other and form a gently arched concavity mesially. Their distal extremities are rounded. The lingual protuberance of the root lacks a nutritive groove but bears a distinct foramen.

*Remarks*. Specimen MMO № 12260/96 exhibits a strong asymmetry that has only been rarely depicted in the literature. Siverson and Lindgren (2005) illustrated a similar tooth of *C. mantelli* from the Turonian of Montana, USA and referred to it as a third lower anterior tooth (a3). Conversely, in the reconstruction of the upper and lower dentition of *C. mantelli* by Welton and Farish (1993) (which was based on disarticulated teeth), teeth with a similar root morphology as MMO № 12260/96 were assigned to the first lateral tooth file (L1) of the upper jaw. It is worth mentioning that teeth with a similar root morphology can be found in the a3 of the extant shortfin mako *Isurus oxyrinchus* Rafinesque, 1810, favoring the interpretation of Siverson and Lindgren (2005). In contrast to the aforementioned authors, whose interpretations were based on isolated teeth, Shimada (1997) did reconstruct the dentition of *C. mantelli* based on partial and nearly complete associated tooth sets from the Niobrara Chalk of Kansas and identified teeth with a similar morphology as symphyseal teeth. However, symphyseal teeth are significantly smaller than MMO № 12260/96, even in large specimens (see Shimada, 1997). Due to this ambiguity, we tentatively follow Siverson and Lindgren (2005) in referring to this tooth as the third lower anterior tooth.

Currently, four species of the genus *Cretoxyrhina* are considered to be valid: *Cretoxyrhina agassizensis* (Underwood and Cumbaa, 2010) from the Cenomanian of Saskatchewan, Canada; *C. denticulata* (Glückman, 1957) from the Cenomanian of the Volga region, Russia; *C. mantelli* (Agassiz, 1835) from the Cenomanian to Coniacian of England; and *C. vraconensis* (Zhelezko, 2000a) from the upper Albian of Mangyshlak, Kazakhstan. The size and morphology of the teeth presented here correspond well with those of *C. mantelli* and can be distinguished from the other three species by the lack of cusplets (cusplets are present in *C. denticulata* and *C. vraconensis*) and the rather robust morphology of the teeth (very gracile in *C. agassizensis*).

Genus ***Acrolamna*** Zhelezko, 1990

*Type species. Acrolamna acuminata* (Agassiz, 1843) *dilatata* Zhelezko, 1990 from the lower Campanian (Upper Cretaceous) of Terekty, western Kazakhstan.

***Acrolamna acuminata*** (Agassiz, 1843)

Figs. 9H-M

*Material*. Eight mostly complete teeth (MMO № 12260/1-8): two anterior teeth (MMO № 12260/1-2), three antero-lateral teeth (MMO № 12260/3-5), and three lateral teeth (MMO № 12260/6-8).

*Description*. Medium sized teeth with anterior teeth up to 15 mm high and 10 mm wide. The crown of the teeth is triangular and very labio-lingually flattened. The main cusp is straight to slightly inclined lingually. In anterior teeth, the crown is symmetrical and acute, whereas it widens and is bent distally towards the jaw commissure in antero-lateral and lateral teeth. The main cusp of the crown is flanked by two oblique lateral heels which are in continuity with the edges of the cusp. The lateral heels can be indistinct (in lateral teeth) to more pronounced (in anterior and antero-lateral teeth), separated by the main cusp only by a shallow concavity. The cutting edge is smooth and extends over the main cusp and both lateral heels. The labial face of the crown is flat, whereas the lingual face is convex. Both faces lack any ornamentations and are smooth. The labial face of the crown slightly overhangs the root. The root is symmetrical in anterior teeth and becomes increasingly asymmetrical with an expanded mesial root lobe in antero-lateral and lateral teeth. It has two divergent lobes with rounded extremities. They are separated by a distinct concavity, which is deep and U-shaped in anterior teeth and becomes shallower in lateral teeth. A lingual protuberance is faintly developed and bears a single foramen but no nutritive groove.

*Remarks*. Two species of *Acrolamna* are currently regarded as valid: *Acrolamna crassicornis* Zhelezko, 1990 from the Coniacian–Santonian from Kazakhstan and *A. acuminata* (Agassiz, 1835) from the Albian of England and the Upper Cretaceous of Germany. In addition, Zhelezko (1990) described a series of species from the upper Albian to Turonian of Kazakhstan and Russia, which he assigned to *Acrolamna* (*A. casei, A. saratovi, A. sulukapjenica* and *A. kolbajensis*). He proposed an evolutionary series from species with lateral cusplets towards species lacking lateral cusplets. However, these species have not yet been described taxonomically and their validity is questionable (Cappetta, 2012). The size and morphology of the specimens from the Akkermanovka locality correspond well to teeth previously illustrated and described as *A. acuminata* (Zhelezko, 1990) and *A*. aff. *acuminata* (Cappetta, 2012; p. 233, Fig. 215A-K).

Family **Mitsukurinidae** Jordan, 1898

Genus ***Scapanorhynchus*** Woodward, 1889

*Type species. Rhinognathus lewisii* Davis, 1887 from the upper Santonian (Upper Cretaceous) of Sahel Alma, Lebanon.

***Scapanorhynchus rhaphiodon*** (Geinitz, 1839)

Figs. 10A-I

*Material*. Twenty-two teeth (MMO № 12260/198-217): six anterior teeth (MMO № 12260/199, MMO № 12260/204, MMO № 12260/207, MMO № 12260/211-212, MMO № 12260/217), 13 antero-lateral teeth (MMO № 12260/198, MMO № 12260/200-203, MMO № 12260/206, MMO № 12260/209-210, MMO № 12260/214-216), and three lateral teeth (MMO № 12260/205, MMO № 12260/208, MMO № 12260/213).

*Description*. The teeth are of moderate to large size with anterior teeth reaching a height of up to 23 mm. The main cusp in anterior teeth is very narrow and strongly sigmoidal in profile view. In lateral teeth the main cusp is slightly more stout, triangular, and faintly inclined distally towards the jaw commissure. In profile view, the cusp is strongly bent lingually in lateral teeth. One pair of small lateral cusplets may be present in anterior teeth, whereas up to two pairs of cusplets are present in lateral teeth. The first pair is well developed while the second pair is poorly developed. Cutting edges are sharp and continuous on the main cusp. The cutting edges of the cusplets are separated from those of the main cusp by a distinct notch. The labial face of the main cusp is flat to slightly convex, while the lingual face is strongly convex and ornamented with distinct striae. The striae at the lingual face of the crown proceed parallel to each other near the base and become flexuous towards the apex. They do not reach the apex of the main cusp but are covering around ¾ of the crown. The labial face is completely smooth. The root is high, mesio-distally compressed and bears divergent and elongated lobes. The root lobes are separated by a deep and U-shaped interlobe area. A nutritive groove is present on the lingual protuberance of the root.

*Remarks*. The genus *Scapanorhynchus* is known from deposits ranging from the Aptian/Albian to the Maastrichtian (Cappetta, 1987, 2012). A plethora of species have been described and assigned to this genus. However, the descriptions of many presumed species are based on poorly preserved specimens and/or are often incomplete or ambiguous (Siverson, 1992b; Biddle, 1993; Bourdon et al., 2011). The material from the Akkermanovka locality corresponds well to teeth described as *S. rhaphiodon* (Geinitz, 1839) and can be separated from other Late Cretaceous taxa by (1) presence of small lateral cusplets in anterior teeth (*S. texanus* (Roemer, 1849) and *S. rapax* (Quaas, 1902) without cusplets; usually no cusplets in anterior teeth of *S. temiricus* Zhelezko, 1987; *S. perssoni* (Siverson, 1992b) with well developed, awl-like cusplets); (2) main cusp S-shaped (not the case in *S. temiricus*); (3) striae on the lingual face of the crown reaching ¾ of the crown (^2^/_3_ in *S. temiricus*) and are weakly developed compared to *S. texanus*. The Santonian species *S. lewisii* (Davis, 1887) is known from articulated skeletons (see Cappetta, 1980) and its teeth closely resemble those of *S. rhaphiodon*. Teeth of *S. lewisii*, however, are smaller in size and have only few but well-marked striae on the lingual face (8–12 in *S. lewisii*, 12–20 in the Akkermanovka specimens).

*Scapanorhynchus rhaphiodon* is the most cited species (Cappetta, 1987; Pollerspöck and Straube, 2022) but its description is based on fragmentary material (isolated anterior cusps) of the Chalk of Lewes, Sussex, England. Woodward (1912) and Herman (1977) figured better preserved material and assigned it to *S. rhaphiodon*, which usually serves as a comparative basis for this species (Siverson, 1992b). Still, some authors are not convinced and regard the diagnostic characters on which *S. rhaphiodon* is based to separate it from the closely resembling species *S. texanus* as weakly founded (Welton and Farish, 1993; Hamm and Shimada, 2002). Bourdon et al. (2011) even went a step further and indicated that *S. rhaphiodon* might be regarded as a *nomen dubium*. In the light of all the ambiguity surrounding *S. rhaphiodon* and the genus *Scapanorhynchus* in general, we urge for a thorough revision of this taxon which was not within the scope of this work. Until then, based on the features described above, we tentatively refer to the Akkermanovka specimens as *S. rhaphiodon*.

Family **Odontaspididae** Müller and Henle, 1839

Genus ***Hispidaspis*** Sokolov, 1978

*Type species. Scapanorhynchus gigas* (Woodward, 1889) from the lower Cenomanian (Upper Cretaceous) of Cambridgeshire, England.

***Hispidaspis horridus*** Sokolov, 1978

Figs. 10J-R

*Material*. Three complete anterior teeth (MMO № 12260/150, MMO № 12260/151, MMO № 12260/153).

*Description*. The largest anterior tooth (MMO № 12260/150) reaches a height of approximately 60 mm. The main cusp is lingually inclined and has a slightly sigmoidal profile. The labial face is slightly convex, whereas the lingual side is strongly convex. The cutting edge is well distinct and continuous, extending across the entire main cusp, and terminates close to the base where it is absent. In the anterior tooth, the base of the labial face of the crown is damaged and thus, neither its ornamentation nor lateral cusplets are preserved. The two smaller anterior teeth are better preserved and do exhibit the following features: specimen MMO № 12260/153 has one pair of small lateral cusplets with slightly lingually curved apexes. Next to the lateral cusplet on the mesial side, there are four small denticles on the labial face of the crown, whereas the distal lateral cusplet only has one adjacent small denticle or spike. Specimen MMO № 12260/151 has a pair of small hook-shaped lateral cusplets near to which there is one additional denticle or spike respectively. Both teeth exhibit a faint ornamentation on the base of the labial face. The labial and lingual side of the crown is smooth in all three teeth. A well-developed neck separates the crown from the root on the lingual side. A distinct protuberance is present on the lingual side of the root, which bears a nutritive groove. The root is only partially preserved in the anterior tooth, lacking most of one of the two root lobes. The preserved root lobe is well separated from the other one, long, and labio-lingually flattened at its distal end. In the antero-lateral teeth, both root lobes are well divergent from each other and a deep V-shaped interlobe area is present. On their distal ends, both lobes are labiolingually flattened. The mesial lobe is slightly longer than the distal lobe.

*Remarks*. The genus *Hispidaspis* is known from the Albian to Maastrichtian of several localities in Eurasia (Zhelezko, 1999; Zhelezko and Kozlov, 1999; Zhelezko, 2000b; Cappetta, 2012). Currently, four species from the Late Cretaceous are considered valid: *Hispidaspis bestobensis* Zhelezko and Koslov in Zhelezko, 2000b from the Turonian of Kazakhstan; *H. gigas* (Woodward, 1889) from the Cenomanian of England; *H. horridus* Sokolov, 1978 from the Turonian–Santonian of Kazakhstan and Russia; and *H. turkestanensis* Zhelezko, 2000b from the Maastrichtian of Uzbekistan. Teeth of *Hispidaspis* have smooth labial and lingual faces and lack striations found in the similar looking lamniform shark genus *Scapanorhynchus*. However, they have a characteristic ornamentation at the base of the labial and lingual side of the crown, especially on the labial face, that can become very salient and can even form spines and tubercles (Sokolov, 1978; Glickman, 1980). The complexity of this ornamentation is the main character to distinguish between the different species (Zhelezko, 2000b). *Hispidaspis horridus* exhibits a simpler ornamentation of the crown base compared to the type species *H. gigas* and *H. bestobensis*. Teeth of *H. horridus* show no longitudinal tubercles on the labial side of the crown, have one pair of hook-shaped lateral cusplets that are bent inward, next to which there may be one or two small denticles. The teeth of *H. turkestanensis* are broader than those in *H. horridus* and are characterized by an even simpler crown structure with no ornamentation at the base of the crowns, and lateral cusplets almost merged with the main cusp. The morphology and size of the teeth described here correspond well with that of *H. horridus* and fall within the known stratigraphic and geographic range of this species.

***Hispidaspis*** cf. ***gigas*** (Woodward, 1889)

Figs. 10S-W

*Material*. Two complete teeth: one anterior tooth (MMO № 12260/149) and one antero-lateral tooth (MMO № 12260/152).

*Description*. The teeth reach a height of up to 40 mm. The main cusp is lingually inclined and has a slightly sigmoidal profile. The labial side is faintly convex, almost flat, whereas the lingual side is strongly convex. The cutting edge is well distinct and extends almost over the entire main cusp except for its base where the cutting edge is absent. Adjacent to the main cusp is a pair of small and straight cusplets and two (on specimen MMO № 12260/149) or three (on specimen MMO № 12260/152) rows of small denticles forming a short (up to 4 mm) comb-like structure parallel to the cutting edge. A distinct ornamentation resembling longitudinal tubercles is present at the bases of the labial face, especially in specimen MMO № 12260/149. Both the labial and lingual sides of the crown are otherwise smooth. The crown is lingually separated from the root by a marked neck. The root exhibits a distinct lingual protrusion which bears a nutritive furrow. The root lobes are divergent and are connected by a shallow, U-shaped interlobe area. Both lobes are labio-lingually flattened distally. The mesial lobe is slightly longer than the distal lobe. The root has a distinct protrusion on which there is a nutritive groove.

*Remarks*. MMO № 12260/149 and MMO № 12260/152 are slightly smaller in size and exhibit a basally more ornamented labial face than the above-described *H. horridus*. Additionally, the shape of the lateral cusplets is straight (not hook-shaped) and the number of the denticles forming the comb-like structure is higher than in the teeth of *H. horridus*. All these features are characteristic of *H. gigas*. Although there were reports of this species from the Santonian of Belgium (Leriche, 1929; Cappetta, 2012) and Kazakhstan (“*Cretaspis* ex gr. *gigas*”; Zhelezko, 1987), the majority of records are restricted to the Cenomanian (Woodward, 1889; Sokolov, 1978). We therefore urge to revise younger reports of this species and to search for additional characters separating both species. Due to the small number of specimens, we tentatively refer to these specimens as *Hispidaspis* cf. *gigas* based on the morphology of the lateral cusplets and the labial ornamentation.

Family **Pseudoscapanorhynchidae** Herman, 1979

Genus ***Pseudoscapanorhynchus*** Herman, 1977

*Type species. Pseudoscapanorhynchus compressidens* Herman, 1977 from the lower Coniacian (Upper Cretaceous) of Maisiéres, Belgium.

***Pseudoscapanorhynchus compressiden*s** Herman, 1977

Figs. 11A-F

*Material*. Thirteen well preserved teeth (MMO № 12260/185-197): ten anterior teeth (MMO № 12260/185, MMO № 12260/187, MMO № 12260/189-195, MMO № 12260/197) and three lateral teeth (MMO № 12260/186, MMO № 12260/188, MMO № 12260/197).

*Description*. The teeth reach a maximum height of approximately 22 mm and a width of up to 6 mm but are usually not exceeding a height of 15 mm. The main cusp is narrow (mesio-distally compressed), lingually inclined, and in most teeth strongly sigmoidal in profile view. The main cusp is straight in anterior teeth and bent slightly towards the commissure in lateral teeth. The teeth have one pair of lateral cusplets. The cusplets are relatively high (up to 5 mm), slightly divergent and lingually inclined. They are situated close to the main cusp and are labially allocated in profile view. The base of the cusplets is situated lower than the base of the main cusp. The lingual side of the main cusp is strongly convex, whereas the labial side of it is only slightly convex. Both sides are smooth and are devoid of any ornamentation. The cutting edge is distinct and stretches throughout the entire main cusp, interrupted only by a deep notch basally that separates the main cusp from the cusplets. The cusplets also exhibit distinct cutting edges. In teeth with a sigmoidal shape, the cutting edge follows the sigmoid outline and is twisted in the apical part of the crown. The root is well developed and at least as high as the main cusp, in most specimens even higher. On its lingual side, the root bears a prominent protuberance which usually exhibits a nutritive furrow, although this feature can be reduced to a faintly developed foramen. The two root lobes are long and mesio-distally compressed, forming a deep V-shaped interlobe area. There are a number of small foramina on the lateral sides of the root next to the crown-root boundary.

*Remarks*. The size and morphology of the Akkermanovka specimens correspond well to the teeth described by Herman (1977) from the upper Cenomanian to Coniacian of Belgium and France. Since then this species has also been described from the Albian of France (as *Leptostyrax macrorhiza*; Biddle, 1993; see Guinot et al., 2013) and Ukraine (Sokolskyi and Guinot, 2021), the Cenomanian of England (Guinot et al., 2013), France (Vullo et al., 2007), and Germany (as *Protolamna acuta* Müller and Diedrich, 1991; see Guinot et al., 2013), the Turonian of France (Guinot et al., 2013), the TuronianeConiacian of Texas, USA (Cappetta and Case, 1999), and the Coniacian of Northern Ireland (Guinot et al., 2013). Sokolskyi and Guinot (2021) noted the generally poor preservation of teeth assigned to this species and suggested that it might in fact represent a complex of several closely related species. The material described here further expands the geographic and stratigraphic range of this taxon.

Family *incertae sedis*

Genus ***Eostriatolamia*** Glückman in Glickman and Zhelezko, 1979 *Type species. Lamna venusta* Leriche, 1906 from the Santonian (Upper Cretaceous) of Lonzée, Belgium.

***Eostriatolamia segedini*** Glückman in Glickman and Zhelezko, 1979

Figs. 11G-R

*Material*. Twenty complete teeth (MMO № 12260/97-116): four anterior teeth (MMO № 12260/97-100), five antero-lateral teeth (MMO № 12260/105-109), and eleven lateral teeth (MMO № 12260/101-104, MMO № 12260/110-116).

*Description*. Teeth are moderately large with the largest teeth reaching a height of up to 12 mm and a width of up to 7 mm. Anterior teeth have a narrow, triangular main cusp which is faintly inclined distally towards the jaw commissure. The width of the crown and the degree of the inclination of the main cusp increase in more distally situated tooth files. In profile view, the main cusp is straight or slightly bent lingually. Some of the teeth show a sigmoidal shape in profile view. Two pairs of cusplets are present adjacent to the main cusp. The medial pair of cusplets is tall and slender and two to three times higher than the distal pair. The cutting edge is sharp and continuous on the main cusp but separated from the cutting edges of the lateral cusplets by a distinct notch. The lingual face of the main cusp is strongly convex and exhibits faintly to well-marked longitudinal folds at the crown base that reach up to one third of the height of the main cusp. The labial face of the crown is slightly convex, and strong enameloid plications occur along the crown base of the teeth. These plications are either confined to the enameloid of the crown or can reach down to the labial face of the root. The labial side of the crown overhangs the root. The root is bilobate with elongated divergent lobes which have rounded extremities. The mesial lobe is longer and wider than the distal lobe. The interlobe area is usually U-shaped but can reach a V-shaped outline. Mesially, a rounded notch separates both root lobes. A pronounced lingual protuberance bears a deep nutritive groove.

*Remarks*. After its erection by Glückman (in Glickman and Zhelezko, 1979), the genus *Eostriatolamia* has been discussed to be a synonym of the genus *Carcharias* Rafinesque, 1810 or *Synodontaspis* White, 1931 (Cappetta, 1987). However, it was pointed out that the dentition of *Eostriatolamia* was primitive compared to the latter two, based on the lack of posterior teeth with a crushing-like morphology (Glickman and Dolganov, 1988; Glickman and Averianov, 1998). Following Glickman and Averianov (1998) and Ebersole et al. (2022), the following species are regarded to be valid and included within the genus *Eostriatolamia*: *Eostriatolamia holmdelensis* (Cappetta and Case, 1975b) from the upper Campanian of New Jersey, USA; *E. paucicorrugata* Underwood and Cumbaa, 2010 from the Cenomanian of Saskatchewan, Canada; *E. segedini* Glückman in Glickman and Zhelezko, 1979 from the Santonian of Kazakhstan; *E. subulata* (Agassiz, 1843) from the Cenomanian of Germany; and *E. venusta* (Leriche, 1906) from the Santonian of Lonzée, Belgium. Teeth from the Akkermanovka locality can be distinguished from *E. holmdelensis* and *E. subulata* by their short and faintly marked longitudinal folds on the lingual crown face (robust and elongated in *E. holmdelensis*/*E. subulata*) and from *E. paucicorrugata* by the presence of strong enameloid plications on the labial face (faint corrugations in *E. paucicorrugata*). Another species, *E. aktobensis* Zhelezko, 1987 was described from the lower Santonian of Mugodzhary, Kazakhstan. Glickman and Averianov (1998) tested the validity of different *Eostriatolamia* species by using quantitative methods (principal component analysis, PCA) and synonymized *E. aktobensis* with *E. segedini* as no quantitative morphological differences could be identified between both species. Indeed, the morphological description of *E. aktobensis* is rather generic and the provided illustrations of the type material is of poor quality, which is why we follow Glickman and Averianov (1998) in regarding this species as a synonym of *E. segedini*.

Based on previously illustrated material including those from the Rybushka Formation (Campanian, Russia), Ebersole et al. (2022) provided a detailed description of the species *E. segedini*. The specimens described here correspond well with those described by Glickman and Zhelezko (1979) and Ebersole et al. (2022), and are thus referred to this species. It should be noted that no anterior teeth with two cusplets were found in the Rybushka Formation and it was proposed that the number of cusplets was shifting from anterior teeth (one pair of cusplets) to lateral teeth (two pairs of cusplets). Glickman and Averianov (1998) stated that two pairs of lateral cusplets were a diagnostic feature for *E. segedeni*, but did not specify if this was true for all tooth positions or confined to distinct tooth positions (i.e., the lateral teeth). Anterior teeth of *E. segedini* found in the Akkermanovka locality did exhibit two pairs of lateral cusplets and we thus propose that the number of cusplets is not only a defining character in lateral teeth to distinguish between both species, but also in anterior teeth. Consequently, the anterior teeth bearing a single pair of lateral cusplets that were previously described by Ebersole et al. (2022) as *E. segedini* are here interpreted as anterior teeth of *E. venusta* instead, which was found in the same locality and is known to have anterior and lateral teeth with a single pair of lateral cusplets (see below).

*Nomenclatural remarks*. According to the International Code of Zoological Nomenclature (ICZN 1999, art. 13), publications intended to erect new taxa after 1930 must provide (or explicitly refer to) a diagnosis in words that includes the objective standard of reference (unambiguous characters) for the application of the name (see also “Principle of Typification”, art. 61). *Eostriatolamia segedini* was originally named by Zhelezko (1977) without providing (or referring to) any description that would unambiguously clarify the taxonomic identity of the new species. Failing to conform to art. 13 of the ICZN (1999), the specific name proposed by Zhelezko (1977) has to be regarded as *nomen nudum*. The proposal of Ebersole et al. (2022) to consider Zhelezko (1977) as the publication in which *E. segedini* was officially erected is in disagreement with the ICZN (1999) and, therefore, it is not accepted here. *Eostriatolamia segedini* was later “validated” by Zhelezko (1987), who, however, provided dubious and contradictory description and illustrations (see Ebersole et al., 2022, for a detailed discussion). Thus, the “name validation” by Zhelezko (1987) does not meet the requirements of the art. 13 and 61 of the ICZN (1999) either. Glickman and Zhelezko (1979) described *E. segedini*, as a new species and provided a proper description and illustration. For the above-mentioned reasons, Glickman and Zhelezko (1979) should thus be considered as the publication in which the genus *Eostriatolamia* and the species *E. segedini* were correctly erected in accordance with ICZN (1999).

***Eostriatolamia venusta*** (Leriche, 1906)

Figs. 12A-H

*Material*. Thirty-two teeth (MMO № 12260/117-148): six anterior teeth (MMO № 12260/118, MMO № 12260/124, MMO № 12260/135, MMO № 12260/137, MMO № 12260/139, MMO № 12260/141), nine antero-lateral teeth (MMO № 12260/123, MMO № 12260/125-126, MMO № 12260/128, MMO № 12260/130-133, MMO № 12260/142), and 17 lateral teeth (MMO № 12260/117, MMO № 12260/119-122, MMO № 12260/127, MMO № 12260/129, MMO № 12260/134, MMO № 12260/136, MMO № 12260/138, MMO № 12260/140, MMO № 12260/143-148).

*Description*. Teeth are moderate to large sized with a height of up to 17 mm and a width of up to 11 mm. Anterior teeth are upright, almost symmetrical, whereas antero-lateral and lateral teeth have slightly inclined to distally hooked main cusps. The main cusp is triangular, widening basally and is lingually inclined to slightly sigmoidal in profile view in anterior and antero-lateral teeth. Lateral teeth usually have straight cusps, but the apex can be bent lingually. One pair of cusplets are present next to the main cusp. The lateral cusplets are thin and slightly curved towards the main cusp in anterior teeth. In lateral teeth, the cusplets are wider, more triangular, and can vary from being divergent to being curved towards the main cusp. The cutting edge is distinct and extends across the main cusp but is separated from the cutting edges of the cusplets by a distinct notch. The lingual face of the main cusp is strongly convex, and very faint longitudinal folds occur at the crown base. The labial face is less convex, and strong enameloid plications occur along the crown base of the teeth. The labial side of the crown overhangs the root. The root is bilobate with elongated, divergent lobes and rounded distal ends. In lateral teeth, the mesial lobe is longer than the distal lobe. The interlobe area is U-shaped in anterior teeth and U to V-shaped in more lateral teeth. In some teeth, a rounded notch is separating both lobes mesially. The lingual side of the root exhibits a protuberance that bears a deep nutritive groove. Several foramina can be present on the labial side of the root.

*Remarks*. The teeth described here closely resemble those assigned to *E. segedini* (see above) but differ from them by only having a single pair of lateral cusplets (two pairs in *E. segedini*) and are thus identified as *E. venusta*. Ebersole et al. (2022) described both species from the Rybushka Formation (Campanian of Saratov) and used the number of cusplets (in lateral teeth) and the breadth of the main cusp as defining characters to differentiate between both species. As mentioned above, anterior teeth of *Eostriatolamia* recovered from the Rybushka Formation always exhibited a single pair of lateral cusplets and it was assumed that anterior teeth in both species had only one pair of lateral cusplets but could be distinguished from each other by their breadth (broader main cusps in *E. venusta*). The teeth of *Eostriatolamia* described here from the Akkermanovka locality have either one or two pairs of lateral cusplets (observable in both, anterior and lateral teeth). However, the tooth breadth appears to be highly variable in both morphotypes and thus, does not qualify as a diagnostic character. Therefore, the number of lateral cusplets is the only unambiguous feature to distinguish between both species. The number of cusplets was also recognized as a useful character to distinguish between the extant lamniform species *Odontaspis ferox* (Risso, 1810) (2–3 pairs) and *O. noronhai* (Maul, 1955) (1 pair) (Humphreys et al., 1989; Pollerspöck and Straube, 2020). However, certain fossil odontaspidids appear to exhibit a variable number of lateral cusplets, e.g., *Odontaspis winkleri* Leriche, 1905 (Maisch IV et al., 2020; Kovalchuk et al., 2023; Popov and Lopyrev, 2023) and even among extant *Odontaspis* species, there is at least one report of a divergent tooth pattern (Ng et al., 2022). Therefore, we cannot completely rule out the possibility that these two species are conspecific. However, the high number of both morphotypes in our sample and the fact that both types are known from numerous localities throughout Eurasia lead us to consider them as two separate but closely related species.

Order **Ptychodontiformes** Hamm, 2019

Family **Ptychodontidae** Jaekel, 1898

Genus ***Ptychodus*** Agassiz, 1834

*Type species Ptychodus schlotheimii* Agassiz, 1834 (*nomen oblitum*), senior synonym of *Ptychodus latissimus* Agassiz, 1835 (*nomen protectum*; see Giusberti et al., 2018) from the Turonian (Upper Cretaceous) of the Central Bohemian Region, Czech Republic.

***Ptychodus rugosus*** Dixon, 1850

Figs. 12I-K

*Material*. One complete tooth (MMO № 12260/332).

*Description*. Specimen MMO № 12260/332 has a rectangular dental crown with an elevated and rounded cusp. In occlusal view, the anterior protuberance is not well-developed and the posterior sulcus is deep. The left crown edge is almost straight, while the right one is curved and tilted posteriorly. Two to three transverse, thin ridges cross the crown apex without reaching the cusp base. A marked dental wear is limited to the cusp apex and the abrasion largely involves all occlusal ridges. Thin wrinkles cover the sides of the tooth cusp and the marginal area; on the latter, they have an almost concentrical pattern. In anterior view, the tooth cusp has a wide base and it is inclined to the left. The crown base is convex with the lateral margins tilted downwards. The root is partially damaged. In posterior view, the preserved root portion exhibits a shallow antero-posterior sulcus; the right and anterior part of the root are missing. In lateral view, the cusp is high with an anterior side inclined forward to reach the short anterior protuberance. The total height of the tooth is approximately 13 mm, while the transverse width is about 21 mm. The crown height is about 10 mm and the cusp height is about 7 mm.

*Remarks*. In contrast to *Ptychodus rugosus* Dixon, 1850, *P. anonymus* Williston, 1900 has ridges running across the entire cusp that reach the marginal area (Hamm, 2020). *Ptychodus altior* Agassiz, 1835, on the other hand, is characterized by a completely smooth occlusal surface on the lateral sides of the dental cusp (Amadori et al., 2019). MMO № 12260/332 exhibits the main species-specific characters of *P. rugosus* with ridges limited to the apex of the cusp and wrinkles on its lateral sides (see also Hamm, 2020) and can thus be distinguished from other cuspidate species (i.e., *P. altior* and *P. anonymus*). Although the specimen documented here is an isolated tooth, its position within the dentition can be inferred from the shape of its crown and cusp (e.g., Amadori et al., 2019, 2020). Based on the asymmetry of the crown, the tooth undoubtedly belongs to the lateral rows of the right hemiarch. The straight side was articulated with the mesial portion of the dentition, while the curved side was articulated with the distal portion. Therefore, the cusp is tilted on the distal side of the crown; this is consistent with the cusp morphologies characterizing associated or partially articulated tooth sets previously described (e.g., Hamm, 2020). The tooth morphology does not provide any information to recognize whether it belongs to the upper dental plate or the lower one.

In Europe, isolated and associated teeth of *Ptychodus rugosus* Dixon, 1850 have only been documented from upper Turonian–lower Campanian outcrops until now (e.g., Dibley, 1911; Woodward, 1912; Dalinkevičius, 1935; Herman, 1977; Guinot et al., 2013; Amadori et al., 2019). Furthermore, this species was reported from upper Cenomanian–lower Santonian strata of England (Longbottom and Patterson, 1987). In North America, the species is well known from the upper Santonian–lower Campanian (e.g., MacLeod, 1982; Welton and Farish, 1993; Hamm, 2020). An unambiguous Asian record of *P. rugosus* exists based on an isolated tooth from the upper Turonian–Coniacian of the Mangyshlak Mountains (Western Kazakhstan; Radwański and Marcinowski, 1996). Moreover, a tooth of *P. rugosus* has been mentioned from the Santonian–Campanian of Sakhalin Island (Pacific Ocean; see Yabe and Obata, 1930; Goto et al., 1996). Additional records of *P. rugosus* in Asia exist, but are here considered ambiguous; teeth from the Santonian of Himedo Park and Kugushima (Japan), which are currently assigned to *P. mammillaris* (Kitamura, 2019) show features similar to those typical for *P. rugosus*.

Class **Actinopterygii** Cope, 1887

Subclass **Ginglymodi** Cope, 1872

Order **Lepisosteiformes** Hay, 1929 (*sensu* Grande, 2010)

Family **Lepisosteidae** Cuvier, 1825 (*sensu* Grande, 2010)

**Lepisosteidae** indet.

Figs. 13A-H

*Material*. Thirteen isolated ganoid scales with varying degrees of abrasion (MMO № 12260/436-446, MMO № 12260/449, MMO № 12260/450) and two abraded dermal bone fragments (MMO № 12260/447, MMO № 12260/448).

*Remarks*. Ten heavily abraded, although strongly ornamented ganoid scales are identified here as belonging to an indeterminate lepisosteiform. Based on differing ornamentation patterns, three separate morphologies are recognized, although we are uncertain if this variation is due to inter- or intraspecific variation. Scales are tentatively assigned to Lepisosteidae as this is the only family of the ganoid-scaled Ginglymodi known to have survived after the Cenomanian–Turonian boundary and to persist up to the modern day (Grande, 2010; Brito et al., 2017). Generally, lepisosteiform scales are not suitable for reliably diagnosing individual gar species (Grande, 2010; Cooper et al., 2021) and several scale morphologies can occur within the same individual (Yang et al., 2013). Although it is very possible that the three morphologies identified in our sample are derived from a single lepisosteiform species, the absence of any associated material cannot prove or disprove this hypothesis, and hence we opt to describe each morphology separately (Lepisosteidae indet. morphology 1, Lepisosteidae indet. morphology 2, and Lepisosteidae indet. morphology 3).

**Lepisosteidae** indet. morphology 1

Figs. 13A-B

*Material*. Five incompletely preserved ganoid scales (MMO № 12260/436, MMO № 12260/439-441, MMO № 12260/445).

*Description*. Scales are rectangular to rhombic and dense in cross section where they are shown to be composed of a dense bony layer and a thinner external coat of ganoin. Ganoin only partially covers the external surface where it forms topographically aligned ridges, with voids between exposing the underlying bony layer. The ganoin ridges are elongate, narrow, and aligned parallel to the longitudinal axis. MMO № 12260/439 is the only example to preserve the large triangular dorsal peg, which is orientated 90° perpendicular to the longitudinal axis of the scale.

**Lepisosteidae** indet. morphology 2

Figs. 13C-E

*Material*. Four broken ganoid scales with differentiating ornamentation to morphology 1 (MMO № 12260/437, MMO № 12260/442-444).

*Description*. Ganoid scales differ to those of morphology 1 in that the ganoin layer covers the entirety of the bony base layer, are slightly denser, and are differentially ornamented with large dome-like protuberances on the external ganoin surface. The ganoin-covered protuberances on specimen MMO № 12260/443 are individually larger but more evenly spaced than those on MMO № 12260/442, seemingly indicative of intraspecific variation, likely due to differentiating placement along the body.

**Lepisosteidae** indet. morphology 3

Figs. 13F-H

*Material*. Two isolated scales (MMO № 12260/468, MMO № 12260/469).

*Description*. Superficially diamond to rhombic shaped, with an evenly smooth ganoine covering on the external surface, which unlike morphologies 1 and 2, is not topographically ornamented. Although some minor enameloid folding is observed on the distal margin of MMO № 12260/469 (Fig. 13H), hinting at some variation in scale architecture along the fish’s flank.

*Remarks*. An absence of marginal spines indicates that these lepisosteiform remains belong to the subfamily Lepisosteidae, and not Obaichthyidae; the latter subfamily is considered to have gone extinct by the end of the Cenomanian (Grande, 2010). All of the scales are abraded, suggesting they were either transported into the environment from elsewhere, or are reworked from older sediments. All extant lepisosteiforms, with occasional exception of the Alligator gar *Atractosteus spatula* (La Cepède, 1803), are restricted to terrestrial fresh and brackish water environments. However, rare gar fossils have been reported in fully marine deposits from the Upper Jurassic to Upper Cretaceous of Mexico (Alvarado-Ortega et al., 2014; Brito et al., 2017), and the Upper Cretaceous (Turonian) of Morocco (Cooper et al., 2021). It is possible that two or more scale morphologies originate from a single taxon, albeit from different regions of the squamation, although this would be impossible to detect in the absence of articulated material. Scales classified under morphology 2 are best comparable with the abdominal scales of the Eocene gar *Atractosteus atrox* Grande, 2010 from the Green River Formation of Wyoming and Colorado (USA), but scales alone are not considered diagnostic for species recognition (Grande, 2010). The Orenburg scales may therefore represent a unique species of Lepisosteidae, perhaps close to *Atractosteus atrox*, although more complete material will need to be identified before this hypothesis can be tested.

Subclass **Teleostei** Müller, 1845

Order **Pachycormiformes** Berg, 1937

Family **Pachycormidae** Woodward, 1895

Genus ***Protosphyraena*** Leidy, 1857

*Type species. Protosphyraena ferox* Leidy, 1857 from the Upper Cretaceous of the English Chalk Group, Sussex, England.

***Protosphyraena*** sp.

Figs. 14A-F

*Material*. Four isolated tooth crowns of varying completeness (MMO № 12260/428-431).

*Description*. Four imperfect tooth crowns with the largest (MMO № 12260/431) measuring up to 28 mm in height and 9 mm in width respectfully. The teeth are tall, labio-lingually compressed with a dagger-like outline, and marked by a prominent, non-serrated anterior and posterior carinae. The smooth lateral faces are broken proximally to expose elliptical cross sections with a dense dentine layer and narrow pulp cavity. The tooth bases are not differentiated (e.g., expanded) from the rest of the crown.

*Remarks. Protosphyraena* is a large streamlined pachycormid with an elongated rostrum (rostrodermethmoid), powerful pectoral fins, and a deeply forked caudal fin giving it a ‘swordfish’-like appearance (Lambers, 1992; Everhart, 2005a; Amalfitano et al., 2017). *Protosphyraena* first appears in the Albian stage of the Lower Cretaceous in Europe, but achieved a near global distribution in the Late Cretaceous (Cenomanian–Maastrichtian) with its remains reported from marine strata in the United Kingdom (Dixon,1850; Woodward,1895, 1912), Belgium (Friedman, 2012), Germany (Diedrich, 2001), Italy (Amalfitano et al., 2017), Spain (Vullo et al., 2009), Sweden (Bazzi et al., 2015), Russia (Solonin et al., 2021), North America (Cope, 1872; Applegate, 1970; Everhart, 2005a; Shimada et al., 2006); Saudi Arabia (Kear et al., 2009), Australia (McNamara et al., 1993), and possibly Nigeria (Vullo and Courville, 2014). Many species of *Protosphyraena* have been proposed, although most are incompletely known by fragmentary skulls and isolated pectoral fins (Lambers, 1992; Amalfitano et al., 2017). The dental morphology is virtually homologous across established species of *Protosphyraena*, meaning that isolated teeth are not diagnostically reliable for species identification. Consequently, we assign the new Orenburg pachycormid teeth as *Protosphyraena* sp. The teeth of *Protosphyraena* sp. are proportionally much larger than the other actinopterygians in the formation, indicating that it was a top macropredator among the actinopterygians in the Akkermanovka assemblage.

Order **Ichthyodectiformes** Bardack and Sprinkle, 1969

Family **Saurodontidae** Cope, 1870

**Saurodontidae** indet.

Figs. 14G-K

*Material*. Eleven isolated tooth crowns (MMO № 12260/397-407).

*Description*. These ichthyodectiform tooth crowns measure up to 18 mm and 5 mm in height and base width, respectively. The crowns are slender and straight with a distinct labio-lingual compression terminating into a medial or slightly posteriorly curved enameloid cap. Structural landmarks comprise a prominent mesial and distal carinae with distinct, slightly undulating lateral striae which extends the entire height of the crown. Teeth with a symmetrical apex have proportionately wider bases than their asymmetrical counterparts, therefore indicating a degree of morphological variation within the jaw.

*Remarks*. Ichthyodectiformes is a diverse clade of teleosts with a near global distribution across both marine and continental deposits from the Middle Jurassic to Late Cretaceous (Bardack, 1965; Bardack and Sprinkle, 1969; Schwimmer et al., 1997; Blanco-Piñó n and Alvarado-Ortega, 2007; Forey and Cavin, 2007; Cavin et al., 2010; Brito and Yabumoto, 2011; Vullo et al., 2012). Teeth recovered from Akkermanovka are morphologically compatible with *Saurodon* and *Gillicus* from the Upper Cretaceous of North America (Blanco-Piñón and Alvarado-Ortega, 2007; Vullo et al., 2012), suggesting that our material belongs in the subfamily Saurodontidae. However, the specimens from Akkermanovka are noticeably larger than those typically seen in saurodontids from North America, inferring they possibly correspond to particularly large individuals or unique species of saurodontid. Contrastingly, the teeth have a lesser height to width aspect ratio when compared with *Cladocyclus* (Forey and Cavin, 2007; Brito and Yabumoto, 2011) and *Xiphactinus* (Schwimmer et al., 1997; De Pasqua et al., 2020). These teeth represent the first known occurrence of the ichthyodectiform family Saurodontidae to be recovered in the Orenburg region of Russia. Dentary fragments of the saurodontid *Saurocephalus lanciformis* were additionally reported from the Saratov Region (Ebersole et al., 2022).

Order **Crossognathiformes** Taverne, 1989

Suborder **Pachyrhizodontoidei** Forey, 1977

Family **Pachyrhizodontidae** Cope, 1872

Genus ***Pachyrhizodus*** Dixon, 1850

*Type species. Pachyrhizodus basalis* Dixon, 1850 from the Cenomanian-Turonian Chalk Group of Lewes, Sussex, United Kingdom.

cf. ***Pachyrhizodus*** sp.

Figs. 14L-O

*Material*. Eight isolated tooth crowns (MMO № 12260/411, MMO № 12260/413-419).

*Description*. The tooth crowns are spherical to sub-spherical in cross section with a slight labio-lingual depression towards the apex. The teeth have a height to base width ratio of 9:5 (9 mm height × 5 mm width). A pair of weakly developed labial and lingual carinae extend the entirety of the crown height. Labial and lingual surfaces are concave and smooth creating a cone-like profile to the teeth. Worn apexes have exposed the dense enameloid layer in a few of the crowns (MMO № 12260/411 and MMO № 12260/418).

*Remarks*. The teeth described here are confidently identified as belonging to a pachyrhizodontid fish with close dental affinities to the widely distributed genus *Pachyrhizodus. Pachyrhizodus* is a speciose predatory fish from Upper Cretaceous deposits in Europe, the Western Interior Seaway (USA), as well as Central (Mexico), Southern America, and New Zealand (Dixon, 1850; Cope, 1875; Forey, 1977; Wiffen, 1983; Hakel and Stewart, 2003; Everhart, 2005a; Giersch et al., 2010; Otero and Suárez, 2022). Although several species have been described, their teeth display only minor variations, meaning that isolated teeth of this genus cannot be reliably assigned to a species. Cope (1875) differentiated the teeth of *P. caninus* (Kansas, USA) from the type species *P. basalis* (Sussex, England) by the presence of a “less swollen and more conical [tooth] in radial section”. By this comparison, the new teeth are closest to *P. basalis*, although the reliability of this character has since been disputed (Forey, 1977; Giersch et al., 2010). The European record of *Pachyrhizodus* comprises *P. basalis* from the English Chalk Group type locality in Lewes, Sussex (England; Dixon, 1850; Forey, 1977), articulated skeletons of *P. subulidens* (Owen, 1840) from the Cenomanian of northeastern Italy (Amalfitano et al., 2020), isolated teeth assigned to cf. *Pachyrhizodus* sp. from the Kristianstad Basin of Sweden (Bazzi et al., 2015), and remains of *Pachyrhizodus* sp. from the Maastrichtian of Belgium and the Netherlands (Friedman, 2012). A partial dentary assigned to *Pachyrhizodus* sp. was recently reported from the Campanian of the Saratov Region of Russia (Ebersole et al., 2022). The new occurrence of *Pachyrhizodus* from the Orenburg region further expands the palaeobiogeographic distribution of this classic taxon in Eastern Europe.

**Pachyrhizodontidae** indet.

Fig. 14P

*Material*. One isolated tooth with an unusual aspect ratio compared to those reported here as cf. *Pachyrhizodus* sp. (MMO № 12260/412).

*Description*. Single pachyrhizodontid tooth that is stout and wide with a low height to base width ratio of 6:5 (6 mm height × 5 mm width). The base of the tooth is greatly expanded, sub-spherical in cross section with a slight compression and a poorly developed pair of carinae which appear to extend the entirety of the crown height. The crown apex is blunt, likely due to feeding wear, creating a triangular transverse profile to the tooth.

*Remarks*. The low crown height to wide base width ratio of specimen MMO № 12260/412 segregates its morphology from cf. *Pachyrhizodus* sp. teeth, which have a contrasting aspect ratio of 9:5. Morphology of specimen MMO № 12260/412 is close enough to cf. *Pachyrhizodus* to assign the tooth to Pachyrhizodontidae, although the latter have tooth crowns which are taller and less expanded at their bases. Therefore, the variation in Pachyrhizodontidae teeth hints at a potential second pachyrhizodontid taxon in the assemblage.

Order **Aulopiformes** Rosen, 1973

Suborder **Alepisauroidei** Regan, 1911

**Alepisauroidei** indet.

Figs. 15A-E

*Material*. Eight well preserved tooth crowns (MMO № 12260/420-427).

*Description*. The crowns measure a maximum of 11 mm in height and 4 mm in width, respectively. The teeth are laterally compressed, fang-like, tall but narrow with a continuous anterior cutting edge forming a pronounced distal postapical barb. The posterior margin below the postapical barb lacks a cutting edge and is instead convex with ornamentation consisting of apico-basal directed striations, akin to those seen in *Enchodus*. The postapical barb is taller than it is wide and extends roughly ⅓ of the crown height from the apex. The teeth are compressed distally but become increasingly wider to form a sub-spherical cross section at their bases. They are mostly hollow in cross section, created from a wide pulp cavity and proportionately thin dentine and acrodine layers.

*Remarks*. Kriwet (2003) described the oldest known alepisauroid teeth from the Barremian-aged, lagoonal Blesa Formation of Iberia (Teruel Province, Eastern Spain), which are morphologically compatible with the new Russian material. According to Kriwet (2003), the general appearance (laterally compressed, fang-like, postapical barb, absence of distal cutting edge) and internal structure (widened at base and mostly hollow) differentiates teeth of alepisauroids from other aulopiform suborders (e.g., Enchodontoidei) and those of closely related neopterygian groups (e.g, Salmoniformes; Hastings et al., 2015). Extant alepisauroids (lancetfishes, lizardfishes, and kin) typically only develop fang-like teeth with postapical barbs on the palatines and ectopterygoids (Uyeno, 1967; Kriwet, 2003), suggesting that the teeth of the Akkermanovka alepisauriform occupied a similar position in the mouth. Compared with the palatal teeth of fossil alepisauroids, the teeth described here are best compatible with the Spanish Barremian material described by Kriwet (2003). Both the Spanish and Russian alepisauriform teeth are morphologically identical, with the exception of the postapical barb being comparatively larger in the Russian examples. Contrastingly, the teeth of the Albian *Apateodus* (Goody, 1969; Patterson, 1993) are massive with a posterior cutting edge but no postapical barb (Kriwet, 2003). Teeth of the Miocene form, *Polymerichthys* Uyeno, 1967, are straight and pointed anteriorly with a double-sided cutting edge and no postapical barb. The Palaeocene-Eocene *Trichiurides* Winkler, 1874 has teeth which are elongate with a prominent postapcial barb, although unlike the specimens described here, have a sigmoidal geometry, are strongly conical in cross section, and usually retain a minute posterior cutting edge (Arambourg, 1952; Casier, 1965; Rayner et al., 2014).

Teeth of the Cretaceous aulopiform *Cimolichthys* (family Cimolichthyidae) are similarly fang-like with widened bases but differ due to the presence of a posterior cutting edge and a comparatively dense enamel and acrodine layer in cross section (Goody, 1970; Kriwet, 2003). Some species of *Enchodus* (suborder Enchodontoidei) do possess barbed teeth on the palatines (Woodward, 1912; Goody, 1976; Silva and Gallo, 2011), although their intraspecific variation ranges from smooth to serrated/folded teeth with or without a posterior cutting edge (Kriwet, 2003). *Enchodus gladiolus* (Cope, 1872) and *E. shumardi* Leidy, 1856 both possess a postapical barb, but not a posterior cutting edge as seen in alepisauroids. Nevertheless, their bases are not expanded and both have narrow pulp cavities unlike alepisauroids (Kriwet, 2003). Furthermore, the internal tooth structure of all enchodontids is highly robust with dense enamel and an extremely narrow to almost closed pulp cavity (Loomis, 1900; Kriwet, 2003). Therefore, the presence of enlarged bases and mostly hollow cross sections of the Akkermanovka specimens exemplifies their exclusion from Enchodontidae.

Fossil alepisauroids are sporadically known from Upper Cretaceous deposits in Europe (Patterson, 1993; Kriwet, 2003) and Russia (Ebersole et al., 2022) with the new specimens from the Santonian–?lower Campanian of Akkermanovka complementing our knowledge of eastern European dispersal timings for alepisauroids in the Late Cretaceous.

Family **Enchodontidae** Woodward, 1901

Genus ***Enchodus*** Agassiz, 1835

*Type species. Esox lewesiensis* Mantell, 1822 from the Upper Cretaceous Chalk Group of Lewes, Sussex, England.

***Enchodus petrosus*** Cope, 1874

Figs. 15F-H

*Material*. 49 palatine and dermopalatine teeth in varying conditions of completeness (MMO № 12260/343-346, MMO № 12260/348-356, MMO № 12260/358-365, MMO № 12260/367-370, MMO № 12260/372-383, MMO № 12260/385, MMO № 12260/388-392, MMO № 12260/394-396, MMO № 12260/432-434).

*Description*. The isolated tooth crowns have a maximum height of 40 mm and an average height to width ratio of 4:1. The crowns are straight with a pointed apex in profile, have a narrow apical width and exhibit a distinctive anteromedial and posterolateral (opposed to posterior) carinae. Distally, the crown apex is symmetrically pointed with postapical barbs absent. The bases of the teeth are slightly expanded with a concave anterior margin forming a superficial reniform to D-shaped cross section. Unworn specimens display subtle striations at the crown base.

*Remarks*. The teeth are assigned to *E. petrosus* based on the following characters: (1) presence of carinae on both the anteromedial and posterolateral faces, (2) concave crown base with striations, (3) tall and slightly sigmoidal crown profile, (4) postapical barb absent. *Enchodus petrosus* is known from the Santonian through to the Maastrichtian in several localities from the USA, Canada, and Mexico (North America) (Goody, 1976; Thurmond and Jones, 1981; Case and Schwimmer, 1988; Everhart, 2005a; Shimada and Fielitz, 2006; Schein and Lewis, 2007; Parris et al., 2007; Cumbaa and Murray, 2008; Carbot-Chanona and Than-Marchese, 2013). Additionally, isolated teeth assigned to *Enchodus* cf. *petrosus* from the Campanian of the Saratov Region of Russia were reported by Ebersole et al. (2022). The new occurrence of *E. petrosus* in Orenburg further expands the species palaeogeographic distribution and reveals an Eastern European dispersal of this taxon during the Santonian–?early Campanian.

***Enchodus*** cf. ***gladiolus*** (Cope, 1872)

Fig. 15I-K

*Material*. Eight isolated tooth crowns (MMO № 12260/347, MMO № 12260/357; MMO № 12260/366, MMO № 12260/371, MMO № 12260/384, MMO № 12260/386, MMO № 12260/387, MMO № 12260/393).

*Description*. The palatine teeth are elongate and slightly sigmoidal, measuring a maximum crown height of 20 mm with smooth and vertical striations along the posterior margin. The teeth are noticeably shorter than those of other *Enchodus* species with a height to width average ratio of 2.5:1. Fine striations are often present along the lateral faces of the crown in unworn examples. The postapical barb is small and weakly developed, whilst the anterior cutting edge extends from the apex to the oval-shaped tooth base. The dentine is very dense in cross-section, resulting in a very narrow pulp-cavity.

*Remarks. Enchodus* cf. *gladiolus* palatine teeth are differentiated from their congeners by the presence of a postapical barb, posterior striations, and a profile that is strongly sigmoidal and comparatively shorter than in *E. petrosus* and *E. ferox* (Goody, 1976; Parris et al., 2007; Becker et al., 2010). The type material of *E. gladiolus* from the Niobrara Chalk (Kansas, USA) was originally described as ‘*Cimolichthys gladiolus*’ by Cope (1872) until it was reassigned to *Enchodus* by Goody (1976) and is the senior synonym of *E. dolichnus* Cope, 1875 (Becker et al., 2010).

*Enchodus gladiolus* has a stratigraphic range from (?)Cenomanian to Maastrichtian and is recorded from numerous localities in the USA, Canada, and Mexico (Cope, 1872; Goody, 1976; Cumbaa and Murray, 2008; Becker et al., 2010; Cumbaa et al., 2010; Gallardo et al., 2012; Jansen et al., 2012; Nagrodski et al., 2012; Ouroumova et al., 2016), with teeth of *E*. cf. *gladiolus* reported from the Campanian of Sweden (Bazzi et al., 2015). Two fragmentary teeth assigned to *E*. cf. *gladiolus* were found in Cenomanian deposits of Nigeria (Vullo and Courville, 2014), however, the material is too fragmentary to validate the occurrence of this species in Africa. Isolated teeth assigned to *E*. aff. *gladiolus* are recorded from the Upper Cretaceous of Peru (Gouiric-Cavalli et al., 2021) and the Maastrichtian of Northern Patagonia (Bogan and Agnolin, 2010), although the identification of the latter occurrence is debated (Gouiric-Cavalli et al., 2021). Teeth assigned to *E*. cf. *gladiolus* were recently reported from the Saratov Region of Russia (Ebersole et al., 2022), and the new occurrence of this species in the Santonian–?lower Campanian of Orenburg further expands the Russian palaeogeographic distribution of this taxon. The aforementioned authors of this study concluded that all the previous reports of *E. gladiolus* outside North America are tenuous and must be reviewed when more and better-preserved material becomes available (Gouiric-Cavalli et al., 2021). We therefore assign the new Orenburg material as *Enchodus* cf. *gladiolus*.

***Enchodus ferox*** (Leidy, 1855)

Fig. 15L

*Material*. One isolated dermopalatine with an incomplete crown (MMO No. 12260/409)

*Description*. A short and robust dermopalatine bone with the dermopalatine tooth situated close to the midpoint of the element, resulting in a slight anterior overhang of the dermopalatine. The tooth crown is laterally compressed with distinct anterior and posterior carinae that each extend close to the crown base. The base of the crown is weakly striated, although the striations are not as well defined as the dermopalatine teeth of *E. gladious* or *E. petrosus*. The distal end of the crown is incomplete.

*Remarks*. Placement of the tooth close to the midpoint of the dermopalatine bone is a unique character to differentiate *E. ferox* from other *Enchodus* species, notably *E. petrosus, E. gladiolus*, and *E. shumardi*, whereby the tooth is located at the anterior extremity of the dermopalatine. The presence of an anterior cutting edge is shared with *E. gladiolus, E. petrosus, E. dirus, E. shumardi, E. lybicus*, and the lizardfish (Ichthyotringoidei) *Apatodus striatus* (Woodward, 1901, 1912, fig. 9; Newbrey and Konishi, 2015), however the addition of a posterior cutting edge extending to the crown base differentiates *E. ferox* from these aforementioned taxa. *Enchodus ferox* is evidently a rare faunal component in the assemblage as only a single individual of this species is detected in our sample.

*Enchodus ferox* is poorly known outside of isolated dermopalatines and tooth crowns, with remains of this species reported from the Santonian–Campanian of the Western Interior Seaway (USA) (Leidy, 1855; Goody, 1976; Robb, 2004; Schein and Lewis, 2007), the Maastrichtian of North Dakota (Hoganson et al., 2019), Alabama (Schein et al., 2013), and the Maastrichtian of Northern Argentina (Bogan and Agnolin, 2010), whilst some authors consider *E. ferox* to be endemic to only the Maastrichtian (Parris et al., 2007). An articulated skull of *E. ferox* from the lower Maastrichtian of Alabama offers the only available evidence for the cranial configuration and morphology of this rare taxon. Specimen MMO No 12260/409 from the Orenburg Oblast represents the first evidence for the occurrence of *Enchodus ferox* in Russia.

***Enchodus*** spp. indet.

Figs. 15M-O

*Material*. An incomplete dermopalatine with broken tooth base (MMO № 12260/408), one partial premaxilla bone retaining dentition (MMO № 12260/435), and a broken indeterminate jaw fragment with base of broken tooth (MMO № 12260/470).

*Description*. The fragment of premaxilla (MMO № 12260/435) measures approximately 11 mm in length with four tooth crowns and three additional broken bases on the dentigerous surface. In cross-section, the bone is oval in the region below the teeth and pinches sharply towards the dorsal margin, which is broken. Narrow slit-shaped foramina for the ethmoid commissure are observed on the lateral face, supporting identification of this element as a premaxilla. Teeth are stout, slightly recurved towards the posterior, and sub-conical to oval in cross section, with no obvious ornamentation or postapical barbs.

Additionally, two partial dermopalatine teeth of uncertain species affinities occur in our sample, the most abraded of which (MMO № 12260/408) displays an expanded D-shaped tooth base and shows evidence of a shallow resorption facet from tooth shed. The tooth is proximal to the base with the cutting edges difficult to interpret due to surface abrasion. The dermopalatine bone (MMO № 12260/409) is fragmentary with only the damaged base of the associated dermopalatine tooth preserved. The ventral surface around the tooth base has a globular topography whilst the dorsal and posterior margins are broken. A series of longitudinal grooves on the ventral face behind the tooth are interpreted as scars left by previous generations of shed dermopalatine teeth. The tooth is laterally compressed, displaying an anterior cutting edge, although the posterior and occlusal surfaces are broken.

**Fig. 9:**
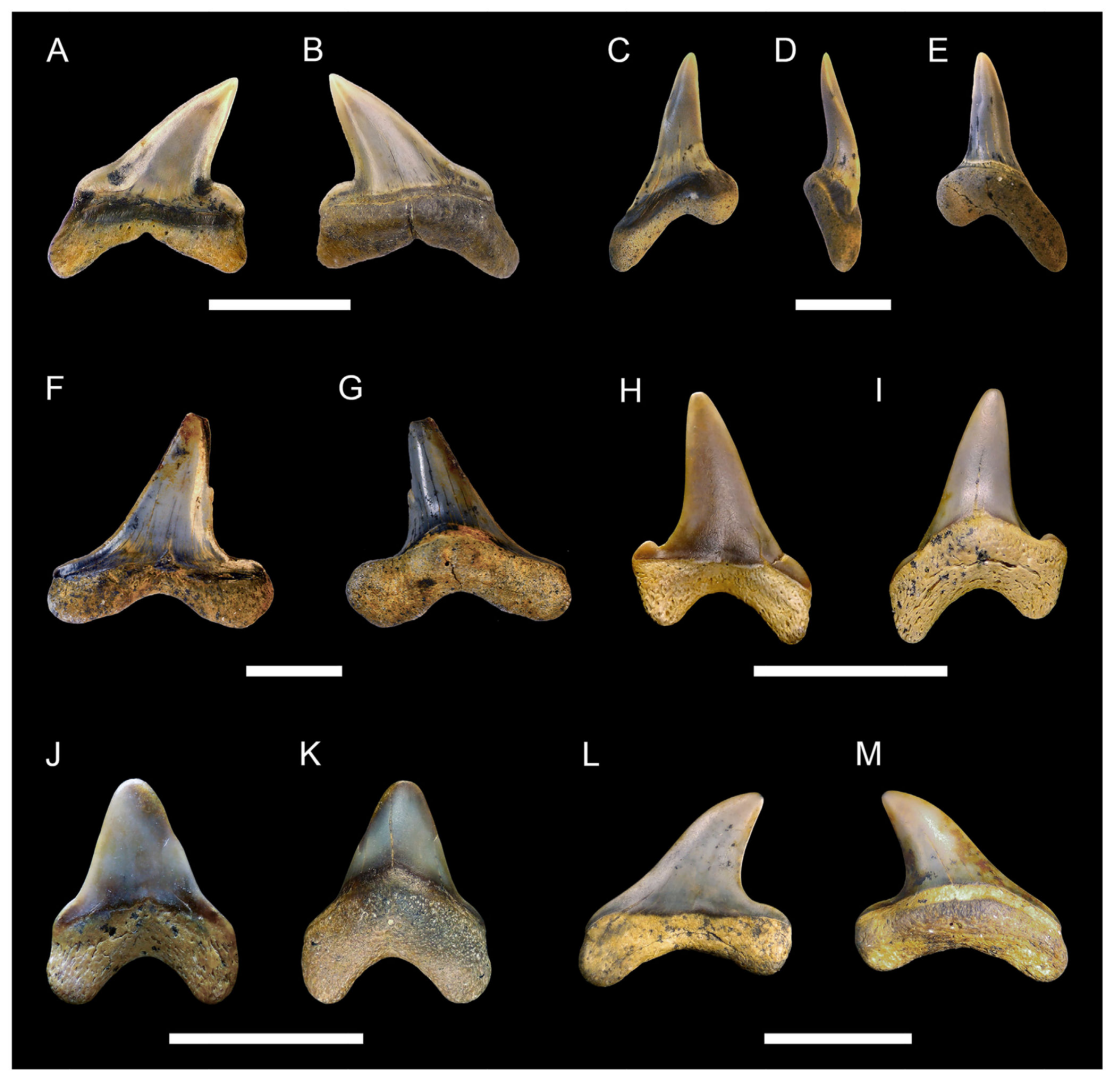
Lamniformes teeth from the Akkermanovka locality in the Orenburg Oblast, Russia. (A-B) *Pseudocorax laevis*: MMO № 12260/184, lateral tooth, (A) labial view, (B) lingual view. (C-G) *Cretoxyrhina mantelli*: (C-E) MMO № 12260/96, lower anterior (?) tooth, (C) labial view, (D) profile view, (E) lingual view; (F-G) MMO № 12260/95, upper lateral tooth, (F) labial view, (G) lingual view. (H-M) *Acrolamna acuminata*: (H-I) MMO № 12260/1, anterior tooth, (H) labial view, (I) lingual view; (J-K) MMO № 12260/5, antero-lateral tooth, (J) labial view, (K) lingual view; (L-M) MMO № 12260/7, lateral tooth, (L) labial view, (M) lingual view. Scale bars = 10 mm.

**Fig. 13:**
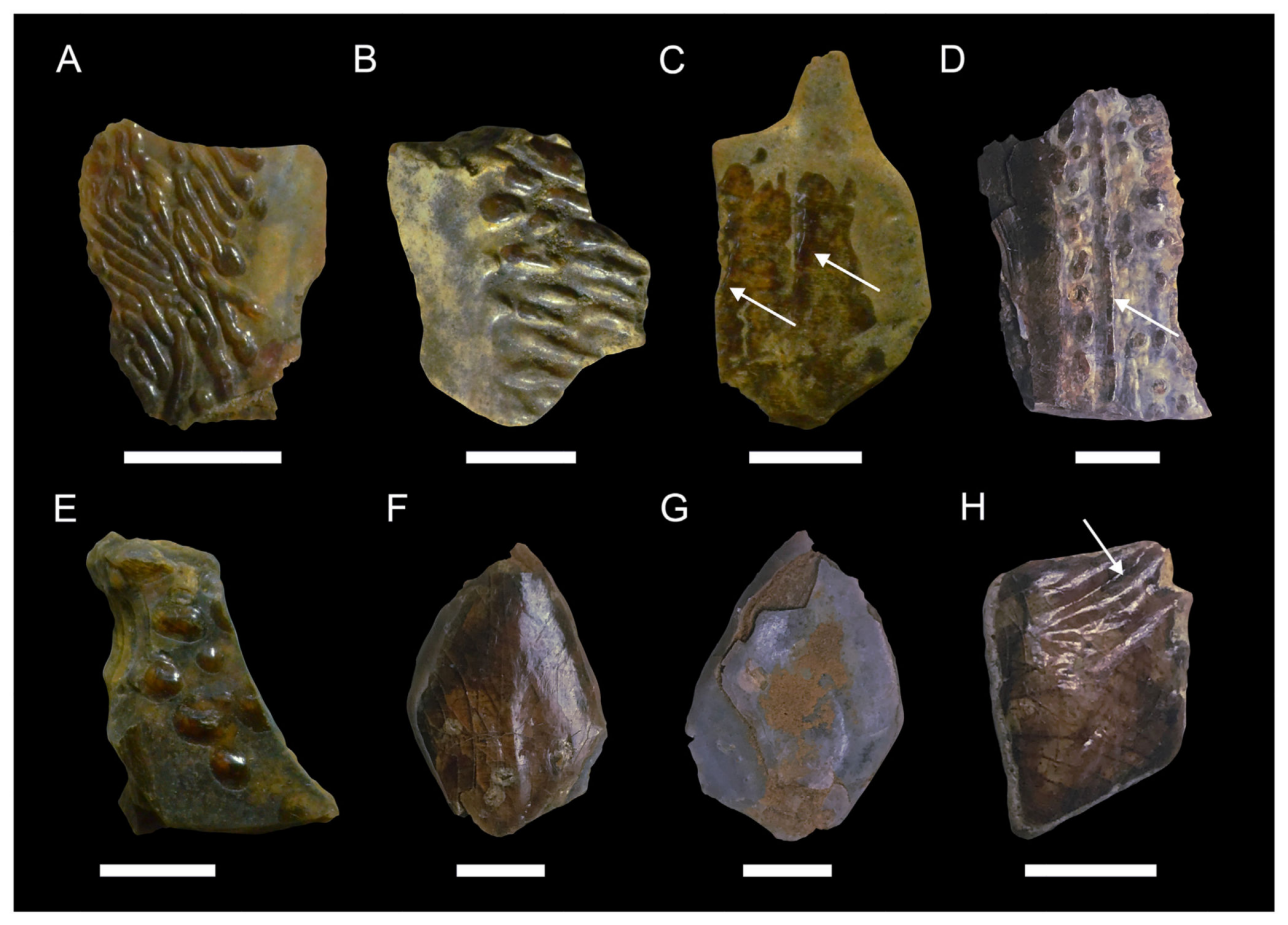
Lepisosteiformes remains from the Akkermanovka locality in the Orenburg Oblast, Russia. (A-H) Lepisosteidae indet.: (A-B) scale morphology 1, (A) MMO № 12260/436; (B) MMO № 12260/445. (C-E) scale morphology 2, (C) MMO № 12260/439, preserving the dorsal peg and two narrow longitudinal ridges on the enamel; (D) MMO № 12260/446, showing numerous bump-like protuberances, intercut by a narrow continuous ridge (arrows); (E) MMO № 12260/443, showing unusually large bump-like protuberances on the external ganoine surface. (F-H) scale morphology 3, (F-G) MMO № 12260/468, (F) external view, (G) reverse view; (H) MMO № 12260/469, note the gentle ganoine folds at the posterior margin (arrow). Scale bars = 5 mm.

*Remarks*. These specimens are assigned conservatively to the genus *Enchodus* on the basis of their comparative morphologies, notably the presence of a narrow pulp cavity and a thick dentine wall, thereby excluding them from Alepisauroidei. The premaxillae of several *Enchodus* species bare a similar dentition to specimen MMO № 12260/435, notably *E. petrosus* (Goody, 1976, n.b. premaxilla absent in author’s reconstruction), *E. lewesiensis* (Dixon, 1850; Woodward, 1901), *E. gladiolus* (Fielitz, 2002), and *E. lybicus* Arambourg, 1952. The anterior dentary of the lizardfish (Ichthyotringoidei) *Apateodus striatus* (Woodward, 1901, 1912; fig. 9; Newbrey and Konishi, 2015) also displays a somewhat similar dental morphology, although that on the premaxilla are not compatible with specimen MMO № 12260/435. The dermopalatine tooth of MMO № 12260/408 is too incomplete to assign it to any species of *Enchodus*, although the D-shaped cross section in MMO № 12260/408 is indicative of *E. petrosus*, although the cross-sectional shape alone is not enough to confidently identify this species.

Class **Reptilia** Linnaeus, 1758

Order **Squamata** Oppel, 1811

Family **Mosasauridae** Gervais, 1853

Subfamily **Tylosaurinae** Williston, 1897

**Tylosaurinae** indet.

Figs. 16A-D

*Material*. One tooth crown (MMO № 12260/453).

*Description*. The crown of the tooth expands towards the base and has a height and maximum width of 28 mm and 17 mm, respectively. The tooth has convex buccal and U-shaped lingual sides. It is moderately labio-lingually compressed and is sub-oval in cross section. The crown bears carinae (cutting edges), one of which is weakly expressed only at the apical part. The carinae are not jagged along the entire length. The surface of the crown is slightly corrugated, covered with shallow but numerous longitudinal ridglets. The ridglets are more pronounced on the lingual and posterior part of the crown. Granulae (i.e., small swellings) are absent at the base of the crown. The top of the tooth is tilted back and inwards. The crown is only moderately recurved posteriorly.

*Remarks*. Specimen MMO № 12260/453 can be attributed to the subfamily Tylosaurinae based on the sub-oval (labio-lingually compressed) cross section, the absence of granulae, the presence of longitudinal ridglets on the crown surface, which are most pronounced on the lingual and posterior parts of the crown, the corrugation of its surface, and the significant expansion of the tooth towards the base. These features are considered highly diagnostic and typical of the subfamily Tylosaurinae (Romano et al., 2019).

Tylosaurinae (and Mosasauridae in general) had a global distribution and are also known from very high latitudes, indicating an endothermic metabolism (Grigoriev and Grabovskiy, 2020). Representatives of this subfamily are known from Turonian–Maastrichtian deposits of North America (Everhart, 2005b), South America (Fernández and Gasparini, 2012; Otero, 2021), Europe (Hornung and Reich, 2015; Romano et al., 2019), Africa (Antunes, 1964; Rempert et al., 2022), Japan (Caldwell et al., 2008), New Zealand (Caldwell et al., 2005), and Antarctica (Fernandez and Martin, 2009; Otero et al., 2017). In Russia, tylosaurine remains were found in Campanian–Maastrichtian deposits of European Russia and the Russian Far East region (Grigoriev, 2017; Grigoriev and Grabovskiy, 2020).

One tylosaurine species, *Tylosaurus rhiphalus* (Bogolyubov, 1910), has been mentioned from the Orenburg Oblast, but its validity has since been questioned and it is possibly a *nomen dubium* (Storrs et al., 2000). The mosasaurid tooth from the Akkermanovka assemblage provides further evidence for the occurrence of Tylosaurinae in the Upper Cretaceous of Orenburg.

Subclass **Sauropterygia** Owen, 1860

Order **Plesiosauria** de Blainville, 1835

Superfamily **Plesiosauroidea** Welles, 1943

Family **Polycotylidae** Williston, 1908

**Polycotylidae** indet

Figs. 16E-P

*Material*. Four isolated teeth (MMO № 12260/454-457).

*Description*. The teeth reach a maximum height of up to 30 mm. At their base, they are round in cross-section and have a diameter of up to 10 mm. The tooth crowns are short, conical, recurved, and medially inclined. The robust teeth exhibit subtle carinae. The tooth crowns are ornamented with pronounced longitudinal ridges. Striations are most prominent at the base of the crown and extend towards the apex, where they become fainter and almost disappear. *Remarks*. Traditionally, Polycotylidae were regarded to belong to the short-necked Pliosauroidea due to their short-neck and ‘pliosauromorph’ body proportions (Persson, 1963). However, cladistic analyses suggest that they are derived Plesiosauroidea (i.e., long necked plesiosaurians) and that the ‘pliosauromorph’ body shape evolved independently in this group (O'Keefe, 2001; Benson and Druckenmiller, 2014; Fischer et al., 2018).

The teeth described here can be attributed to the family Polycotylidae based on their roundness in cross-section and the tooth crown ornamentation of very pronounced longitudinal ridges on the crown surface. In elasmosaurids, the crowns of the teeth are oval or D-shaped in cross-section, and the longitudinal ridges on their surface are thinner and less pronounced. The remains of members of the family have been found in the Aptian–Maastrichtian of North America (O'Keefe, 2004; Schumacher and Martin, 2016), South America (O’Gorman and Gasparini, 2013), Europe (Persson, 1959), North Africa (Bardet et al., 2003; Allemand et al., 2018), Japan (Sato and Storrs, 2000), New Zealand (O’Gorman and Otero, 2023), Australia (Kear, 2005), and Antarctica (Novas et al., 2015). In Russia, polycotyloid remains have been found in Albian–Campanian deposits in European Russia and Eastern Siberia (Pervushov et al., 1999; Storrs et al., 2000; Arkhangelsky et al., 2007; Zverkov et al., 2023a).

Polycotylid remains are well known from the Orenburg Oblast and were assigned to the genus *Polycotylus* (Efimov et al., 2016). Based on fragmentary skeletal material, Bogolyubov (1912b) described two polycotylid taxa from the Orenburg Oblast, *P. orientalis* Bogolyubov, 1911 and *P*. cf. *balticus* Bogolyubov, 1911, but both species are now regarded as *nomina dubia* (Adams, 1997; Storrs et al., 2000). Another polycotylid, *P. sopozkoi* Efimov, Meleshin and Nikiforov, 2016, was described from an lower Campanian deposit near the village of Izhberda in the Gaiskii District (Orenburg Oblast), but was recently discussed to be a junior synonym of *P. latipinnis* (Zverkov et al., 2023b). Teeth are not regarded to be diagnostic beyond family level in this group (Storrs et al., 2000) and in the absence of more complete skeletal material, we conservatively leave this taxon in open nomenclature as Polycotylidae indet.

**Plesiosauria** indet.

Figs. 16Q-R

*Material*. Four fragmentary tooth crowns (MMO № 12260/458-461).

*Description*. Based on the fragments available, the tooth crowns of the Akkermanovka plesiosaur teeth are ornamented with longitudinal ridglets and ridges. Due to the extreme fragmentation of the material, it is difficult to determine the relative length of the crowns and their cross-sections.

*Remarks*. Due to significant incompleteness, the available tooth fragments are assigned to Plesiosauria indet. However, pronounced longitudinal ridglets and ridges on their surface may indicate that they belong to a member of the family Polycotylidae.

## 5 Discussion

Several Upper Cretaceous outcrops were previously reported from the Orenburg Oblast, yet these studies usually focused on marine reptiles, whereas the fish fauna was left unreported (Bogolyubov, 1910, 1912b; Efimov et al., 2016). Our study is the first to provide a detailed systematic description of the fossil fish fauna from the Orenburg Oblast (Russia, Southern Urals) and describes a diverse vertebrate assemblage consisting of cartilaginous fishes (Chondrichthyes), bony fishes (Actinopterygii), and marine reptiles (Mosasauridae and Plesiosauria).

### 5.1 Taxonomic diversity and abundance of fossil marine vertebrates from the Akkermanovka locality (Orenburg Oblast)

**Fig. 17:**
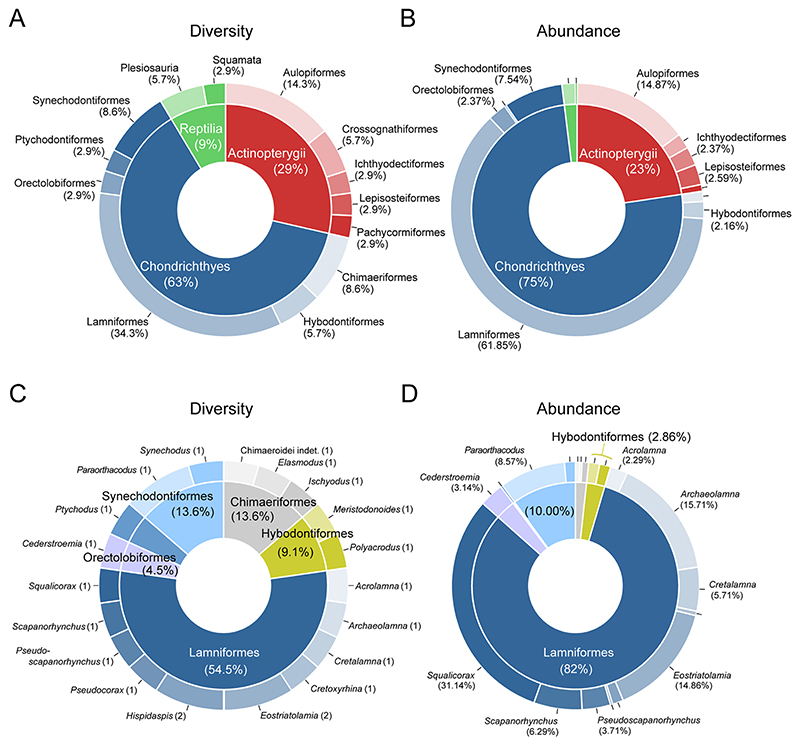
Pie charts depicting the (A) marine vertebrate diversity, (B) relative abundance of marine vertebrate remains, (C) chondrichthyan diversity, and (D) relative abundance of chondrichthyan remains in the Late Cretaceous Akkermanovka assemblage (Orenburg Oblast, Russia).

In total, 35 taxa are identified in the present study. Chondrichthyans account for the majority of the taxic diversity (22 taxa, 62.8%), followed by actinopterygians (10 taxa, 28.6%) and marine reptiles (three taxa, 8.6%; Fig. 17A; Table 1). Chondrichthyan remains are also by far the most abundant fossils recovered from the studied horizon, making up three-quarters (75.4%) of the assemblage; whereas actinopterygians and marine reptiles only make up 22.6% and 2%, respectively (Fig. 17B). Whilst these values are based on a systematic approach to the identified materials, we cannot exclude the possibility that a taphonomic sorting and/or collection bias has affected our sample.

Chondrichthyans are represented by six orders: Chimaeriformes, Hybodontiformes, Synechodontiformes, Orectolobiformes, Lamniformes, and Ptychodontiformes. Lamniform sharks are by far the best represented group (Figs. 17A-B) in terms of both taxic diversity (12 taxa; 34.3%) and relative fossil abundance (61.85%). Lamniformes make up more than half of all chondrichthyan species (Fig. 17C) and are by far the most dominant chondrichthyan group, making up more than 80% of all chondrichthyan remains retrieved from Akkermanovka (Fig. 17D). The order Lamniformes is thought to have emerged in the Jurassic Period (Jambura et al., 2019), but it was not before the Valanginian–Barremian (Early Cretaceous), when basal lamniforms became more diverse and widespread (Rees, 2005; Kriwet et al., 2008; Carrillo-Briceño et al., 2019). Subsequently, this group underwent a dramatic diversification during the Cenomanian, resulting in lamniform sharks becoming a dominant and diverse group during the Late Cretaceous and Palaeogene (Guinot and Cavin, 2016; Condamine et al., 2019), which is also highlighted by their dominance in the faunal composition of Akkermanovka.

Although no index fossils were found within the layers of the Akkermanovka locality (see Geological setting), a Late Cretaceous age (possibly Santonian–Campanian) as reported from other localities in the Orenburg Oblast (Efimov et al., 2016; Averianov et al., 2021a,b, 2022; Skutschas et al., 2022) is very likely, especially when considering the diverse fish assemblage described in this study (see “Biostratigraphic implications” below). Among the lamniform sharks in this assemblage, teeth of *Squalicorax kaupi* were the most abundant fossil remains (31.14% of all chondrichthyan taxa), followed by *Archaeolamna* ex gr. *kopingensis* (15.71%), and *Eostriatolamia* spp. (14.86%). A similar faunal composition was described from the Campanian Rybushka Formation in the Saratov Region (Ebersole et al., 2022), but large lamniform sharks appear to be less diverse and less abundant compared to the Akkermanovka assemblage. *Archaeolamna* ex gr. *kopingensis, Eostriatolamia* spp., and *Cretalamna* spp. were reported to be major components in the Saratov Region, whereas *Squalicorax kaupi* was rather rare (three teeth) and other large lamniform taxa such as *Cretoxyrhina* and *Acrolamna* were not recorded.

The lamniform shark *Pseudoscapanorhynchus compressidens* is represented by a dozen well preserved teeth and is the first record of this species in Russia, extending its geographic range to the eastern-most part of Europe. The second most abundant chondrichthyan group are sharks of the order Synechodontiformes (10%), whereas the remaining four chondrichthyan orders are much less abundant (together less than 10%). Teeth of the genus *Paraorthacodus* are the most abundant among synechodontiform sharks and make up 8.57% of all chondrichthyan remains. The perceived dominance of large toothed taxa and the absence of batomorphs (rays and skates) and small sized sharks like cat sharks (Scyliorhinidae, Carcharhiniformes) in Akkermanovka (but also other Russian localities) might be the result of a collection bias, because the used mesh size of 4 mm would not allow to collect small sized taxa, or taxa with small teeth.

Five orders of Actinopterygii are identified from the Akkermanovka locality: Lepisosteiformes, Pachycormiformes, Ichthyodectiformes, Crossognathiformes, and Aulopiformes. Among bony fish, the order Aulopiformes is the most diverse and abundant order, contributing almost 15% to the total diversity and abundance respectively. It makes up more than half of the bony fish material described here and almost 50% of the bony fish diversity in Akkermanovka. Aulopiformes is represented by the taxa *Enchodus petrosus, E. ferox, E*. cf. *gladiolus, E*. spp. indet., and Alepisauroidei indet. To the best of our knowledge, *E. petrosus* and *E*. cf. *gladiolus* have only been reported from the Campanian Rybushka Formation in the Saratov Oblast (Ebersole et al., 2022) but as yet from nowhere else in Russia. *Enchodus ferox* has not previously been reported from Russia. Other members of the genus *Enchodus, E. faujasi* Agassiz, 1843 and *E. halocyon* Agassiz, 1835 were reported from the Saratov Oblast (Sinzow, 1899), but the material is lost and therefore the occurrence of these species in this region could not be confirmed (Ebersole et al., 2022).

Marine reptiles are not very abundant in Akkermanovka and are only represented by two families, Mosasauridae (Squamata) and Polycotylidae (Plesiosauria). Remains of marine reptiles have been commonly reported from the Mesozoic epicontinental marine deposits of western and eastern Russia but are usually poorly preserved and taxa were left in open nomenclature (Storrs et al., 2000; Arkhangelsky et al., 2007; Grigoriev and Grabovskiy, 2020; Zverkov et al., 2023a). In the Akkermanovka assemblage, marine reptiles are only represented by teeth and do not allow identification beyond family or subfamily levels. Whereas polycotylids are well known from Orenburg (Bogolyubov, 1912b; Efimov et al., 2016) and Russia in general (Pervushov et al., 1999; Storrs et al., 2000; Arkhangelsky et al., 2007; Zverkov et al., 2023a), tylosaurines (Mosasauridae) have rarely been reported to date (Bogolyubov, 1910; Grigoriev and Grabovskiy, 2020). The marine reptiles of the Akkermanovka assemblage therefore further contributes to our knowledge of the spatio-temporal distribution of these taxa in Russia.

**Fig. 18:**
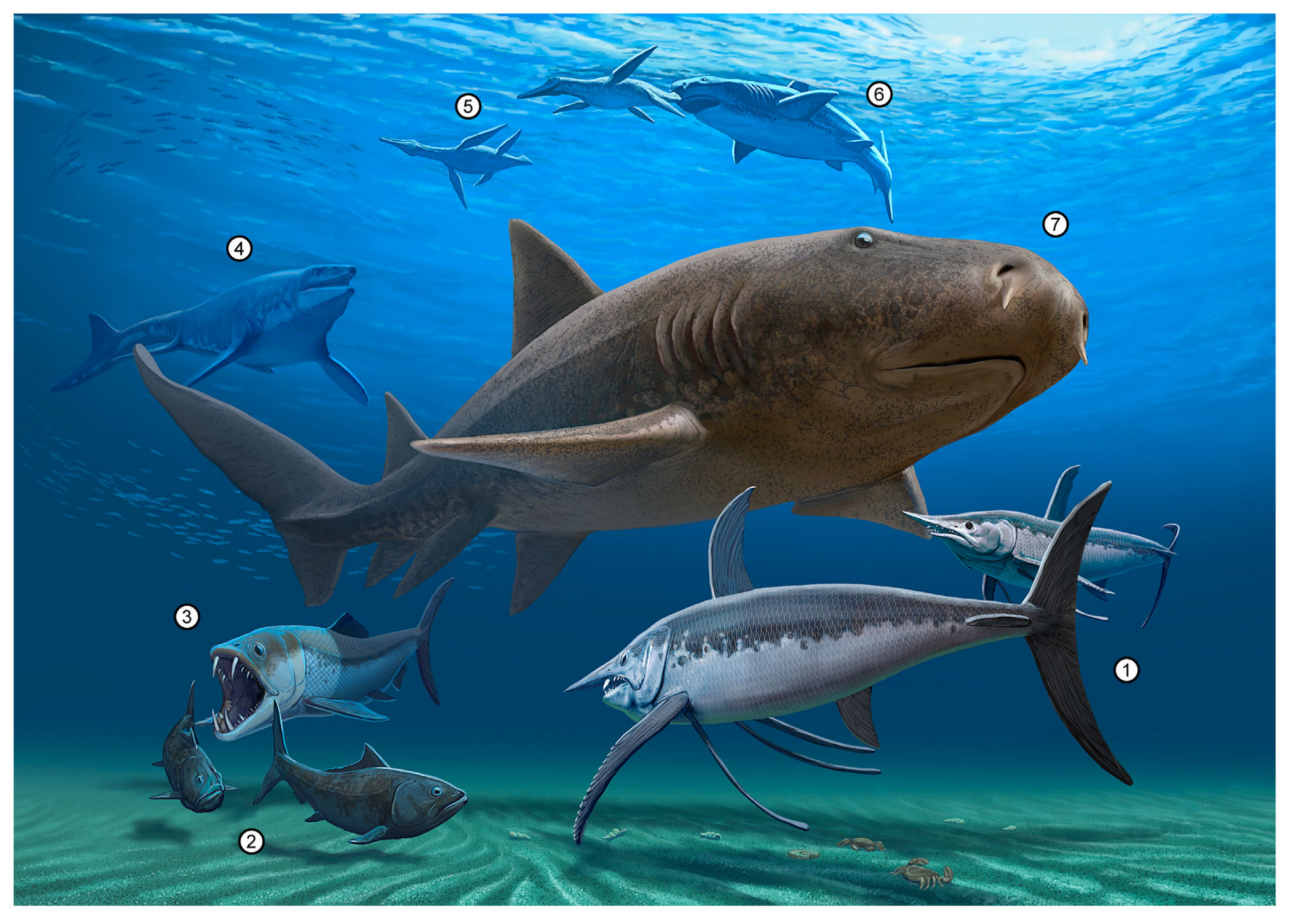
Palaeoenvironmental reconstruction of the Akkermanovka locality during the Late Cretaceous: (1) *Protosphyraena*, (2) *Pachyrhizodus*, (3) *Enchodus*, (4) Tylosaurinae, (5) Polycotylidae, (6) *Squalicorax kaupi*, (7) *Ptychodus rugosus*. Illustration by Sergey Krasovsky.

Upper Cretaceous deposits in the Southern Urals are dominated by shallow marine facies, coastal and shore deposits (Amon et al., 1997), which is also the case in Akkermanovka. The high faunal diversity and the abundance of large-sized predators like lamniform sharks, *Ptychodus rugosus*, marine reptiles, and *Protosphyraena* are indicative for a highly productive, shallow marine environment in Akkermanovka during the Late Cretaceous (Fig. 18). The apparent absence of batomorphs and bottom-dwelling sharks (with the exception of the orectolobiform shark *Cederstroemia nilsi*) in Akkermanovka and their low diversity in other Southern Ural deposits (Glickman and Zhelezko, 1979, 1990) might be the result of the size of mesh used (4 mm) and thus could be explained by a collection bias. Therefore, future studies should focus more on vertebrate micro-remains to complement our knowledge of the biodiversity and palaeoecology of the Orenburg Region during the Late Cretaceous.

### 5.2 Biostratigraphic implications

The Akkermanovka assemblage includes typical Late Cretaceous groups and species that are known to occupy a wide stratigraphic and palaeobiogeographic range. The Southern Urals have well known Upper Cretaceous assemblages that serve as important reference sections for foraminifera and radiolaria in Cenomanian, Coniacian, Santonian, Campanian, and Maastrichtian strata (Amon et al., 1997). Several chondrichthyan taxa that are present in the Akkermanovka assemblage were found associated with foraminifera, radiolaria, and molluscs in Santonian strata in this region, i.e., *Acrolamna acuminata, Cretoxyrhina mantelli, Eostriatolamia segedini, E. venusta, Hispidaspis* cf. *gigas, Pseudocorax laevis, Scapanorhynchus rhaphiodon*, and *Squalicorax kaupi*.

*Squalicorax lindstromi* has been regarded as a typical early Campanian faunal element in the Southern Urals (Glickman and Zhelezko, 1979; Zhelezko, 1987, 1990; Amon et al., 1997), but is regarded here as a synonym of *S. kaupi* (see remarks to *S. kaupi*). Therefore, the presence of *S. kaupi* in our assemblage could indicate both a Santonian or an early Campanian age for the Akkermanovka assemblage. However, none of the other taxa that are characteristic for lower Campanian strata (“*Squalicorax lindstromi* Zone”) in the Southern Urals are present in the Akkermanovka assemblage (see Amon et al., 1997). Nonetheless, certain taxa from Akkermanovka are also reported from Campanian deposits in Russia which are outside the Southern Urals; i.e., *Eostriatolamia segedini, E. venusta, Pseudocorax laevis*, and *Squalicorax kaupi* (Averianov and Popov, 1995; Ebersole et al., 2022). *Acrolamna acuminata* is regarded as a typical Santonian taxon in the Southern Urals and Russia in general (Zhelezko, 1987, 1990; Amon et al., 1997), however, similar morphotypes were also described from Campanian outcrops in the Southern Urals region: i.e., *A*. cf. *acuminata, A. acuminata dilatata* (Zhelezko, 1990; Amon et al., 1997). *Cretoxyrhina mantelli* and *Ptychodus rugosus*, on the other hand, are only known from lower and upper Santonian strata of the Southern Urals (Glickman and Zhelezko, 1979, 1990; Amon et al., 1997). However, scattered reports of post-Santonian occurrences of *C. mantelli* exist outside Russia in Europe (Siverson, 1992b; Sørense et al., 2013) and North America (Siverson and Lindgren, 2005; Cook et al., 2017).

Based on the taxa discussed above, a Santonian–?early Campanian age is suggested for the vertebrate assemblage in this study. This is also in agreement with the known stratigraphic range of the remaining vertebrate taxa. Although the stratigraphy of Upper Cretaceous deposits in Orenburg is still poorly understood, recent advances revealed the presence of Turonian–Santonian, Campanian, and lower Maastrichtian strata (Lyadsky et al., 2013; Nekhaev et al., 2021). The lithological composition of the fossil bearing layer in Akkermanovka best compares to Turonian–Santonian and Campanian strata reported from the Orenburg region and is thus in agreement with our determination of a Santonian–?early Campanian age of the Akkermanovka assemblage.

### 5.3 Palaeobiogeographic implications

The geographical border between Europe and Asia is marked in Russia by the Ural Mountains. The Orenburg Oblast is situated in the western part of the Southern Urals and thus represents the eastern-most part of Europe (Fig. 1). During the Late Cretaceous, the Southern Ural area of the Orenburg Oblast also was located in the eastern-most region of the Russian Platform (“Eastern European Platform”). Since the Cenomanian marine transgression, a relatively shallow epicontinental sea covered a vast area of the Eastern European Platform (Russian Platform) with large islands only in the southern-most area (Amadori et al., 2022). Marine sediments of this region were deposited in the eastern peri-Tethyan basin, which was connected to the West Siberian Sea and to the marine basins of Central Europe (Baraboshkin et al., 2003; Amadori et al., 2022). The Russian platform was characterized by frequent sea level fluctuations and sea connections were temporarily cut off. The largest sea level transgressions occurred in the Santonian and early Campanian (Vinogradov, 1975; Baraboshkin et al., 2003; Kuzmin et al., 2015).

Of the 35 reported taxa in this study, 26 could be identified at least on genus level (21 Chondrichthyes, five Actinopterygii) and contribute to our understanding of the palaeogeographic distribution of these taxa. Due to the fragmentary nature of the material, the marine reptiles could only be identified on family level (Polycotylidae) and/or subfamily level (Tylosaurinae; Mosasauridae), both of which are known to have occurred globally (Table 1). Four of the identified chondrichthyan taxa are endemic to Europe (*Cederstroemia nilsi, Acrolamna acuminata, Cretalamna sarcoportheta, Eostriatolamia segedini*). Teeth of *Cretalamna* are known from Albian to Ypresian deposits worldwide and were traditionally assigned to the type species, *C. appendiculata*. Siversson et al. (2015) identified *C. appendiculata* as a species complex that included at least eight species. *Cretalamna sarcoportheta* was erected based on isolated teeth from the Campanian of Sweden but is also known from Campanian deposits of Belgium and France (Siversson et al., 2015). *Cretalamna* cf. *sarcoportheta* was described from Campanian deposits of Russia (Ebersole et al., 2022) and Texas (Schubert et al., 2017), indicating that this species had a wider geographic distribution than is currently acknowledged.

The remaining 22 fish taxa from the Akkermanovka locality were also reported from at least one other continent (Table 1): Africa (3 taxa in common), Antarctica (1 taxon in common), Asia (17 taxa in common), Australia (1 taxon in common), North America (15 taxa in common), South America (2 taxa in common), and Oceania (1 taxon in common). Our data shows a significant overlap of the Santonian–Campanian fauna from Akkermanovka (Orenburg Oblast, Southern Ural) with those from North America, Europe, and Asia from the same time interval, whereas only little overlap exists between the fauna from Akkermanovka and those known from the Southern Hemisphere (Table 1). The similarities between the assemblages within the Northern Hemisphere indicate a wide east–west distribution of the Akkermanovka marine fauna during the Santonian–Campanian interval. A similar trend was observed for chondrichthyan and actinopterygian taxa from the upper Campanian Rybushka Formation near Beloe Ozero in the Saratov Oblast (Russia), which also exhibited high similarities with North American and other European assemblages (Ebersole et al., 2022). The main difference between the assemblages described from the Rybushka Formation and from Akkermanovka is the lack of overlap with Asian assemblages in the Rybushka Formation assemblage. The higher similarity between the taxa from Akkermanovka and Asia can best be attributed to the fact that the Orenburg Oblast is bordering Asia and many of the taxa described here are known from other Santonian and lower Campanian deposits of the Southern Urals of Kazakhstan (Zhelezko, 1987; Amon et al., 1997), which belongs to central Asia.

The epicontinental seas of the western peri-Tethys (nowadays Europe) and the Russian platform were well connected during the Late Cretaceous, which is reflected by the similarities between the assemblage from Akkermanovka (Orenburg Oblast) and other Santonian–Campanian faunas in Europe. Shallow-water connections also occurred between the North American areas and the western-most peri-Tethys before the opening of the northern-most part of the Atlantic Ocean around 60–50 mya (Palaeogene; Ladant et al., 2020; Upton, 2021; Wilmsen et al., 2021). The east–west distribution documented here for major elements of the Akkermanovka fauna in the Santonian–Campanian could thus be the result of one or multiple dispersal events across northern epicontinental seas (40°–60°N), such as the “East Greenland Seaways”, “proto-Labrador Sea”, and “Hudson Seaway” during the Late Cretaceous (Ladant et al., 2020; Radmacher et al., 2020; Neubauer and Harzhauser, 2022). A cooling of the surface waters was hypothesized in the area of the modern Northern Atlantic, especially at a palaeolatitude ranging between 30° and 60° N throughout the Late Cretaceous (Ladant et al., 2020). Although water temperature fluctuations can strongly influence the spatial distribution of marine faunas (Maduna et al., 2020; Amadori et al., 2022), a lower water temperature at higher latitudes across Santonian–Campanian sea-ways might not have been insuperable for taxa that are interpreted to be (regional) endothermic, like pachycormiform fishes (see Johanson et al., 2022), some lamniform sharks (see Ferrón, 2017), as well as plesiosaurs and mosasaurs (see Leuzinger et al., 2023). Moreover, lower water temperatures at higher latitudes for Santonian–Campanian seaways between North America and Europe could have been partially mitigated by the warm “proto-Gulf stream”, which was running along the surface waters from the Gulf of Mexico towards northwestern Europe during the Late Cretaceous (Linnert et al., 2011; Wilmsen et al., 2021).

Most of the taxa documented here from Akkermanovka are currently unknown from the Southern Hemisphere. The faunal distribution highlighted in Table 1 indicates a possible lack of north-south dispersal (or vicariance) events for the taxa that inhabited the epicontinental sea surrounding the Akkermanovka region during the Late Cretaceous. Similar biogeographic trends were reported for bony fishes before (Cavin, 2008). The reason for the lack of south-west dispersal might be based on the fact that the separation between Laurasia and Gondwana already took place in the Late Jurassic and therefore exchange of coastal species between both hemispheres was limited (Cavin, 2008). However, a global sampling bias cannot be ruled out, as Late Cretaceous marine faunas are better known from the Northern Hemisphere than they are from the Southern Hemisphere. More taxonomic studies from the Southern Hemisphere will be crucial to better understand the north–south distribution of marine faunas and their significance in a more global frame.

## 6 Conclusions

Despite the high abundance of fossil fish-bearing Upper Cretaceous deposits in Russia, many of these important horizons have remained unstudied or poorly described until only recently. This study provides the first comprehensive taxonomic description of marine fishes from the Upper Cretaceous of the Orenburg Oblast, Russia. In total, 35 vertebrate taxa were identified: 22 chondrichthyan taxa, ten actinopterygians, and three types of marine reptiles. Their occurrence in the Akkermanovka assemblage expands the geographic and stratigraphic range for several taxa, i.e., *Cederstroemia nilsi, Cretalamna sarcoportheta, Meristodonoides* sp., and *Pseudoscapanorhynchus compressidens* (Chondrichthyes) and *Enchodus petrosus, E. ferox, E*. cf. *gladiolus*, and cf. *Pachyrhizodus* sp. (Actinopterygii). Remains of a lepisosteid gar within the formation is noteworthy as gars are typically confined to fresh and brackish waters, with the fully-marine environment of the Akkermanovka quarry representing an unusual environment for gar occupation.

Lamniform sharks made up the majority of the assemblage in both diversity and abundance, reflecting the astoundingly high taxic diversity and dominance of this group after their diversification in the Cenomanian. The absence of any small bodied fishes or bottom feeding species is likely the result of a collecting bias, although large-bodied benthic feeding actinopterygians with durable dentitions (e.g., Pycnodontiformes) are surprisingly absent from the assemblage. Nonetheless, the high faunal diversity and the presence and abundance of large sized predators, e.g., *Squalicorax kaupi, Acrolamna acuminata, Cretoxyrhina mantelli, Ptychodus rugosus*, and *Protosphyraena* sp. indicate a highly productive shallow marine environment in Akkermanovka during the Late Cretaceous.

Based on the described faunal assemblage, a Santonian–?early Campanian age of the Akkermanovka locality is proposed. We were able to identify significant overlaps of the faunal assemblage of Akkermanovka with other Upper Cretaceous localities from Europe and North America. This study further adds to our knowledge about east–west dispersals and suggests that this pattern was a common phenomenon during the Late Cretaceous.

## Figures and Tables

**Fig. 4:**
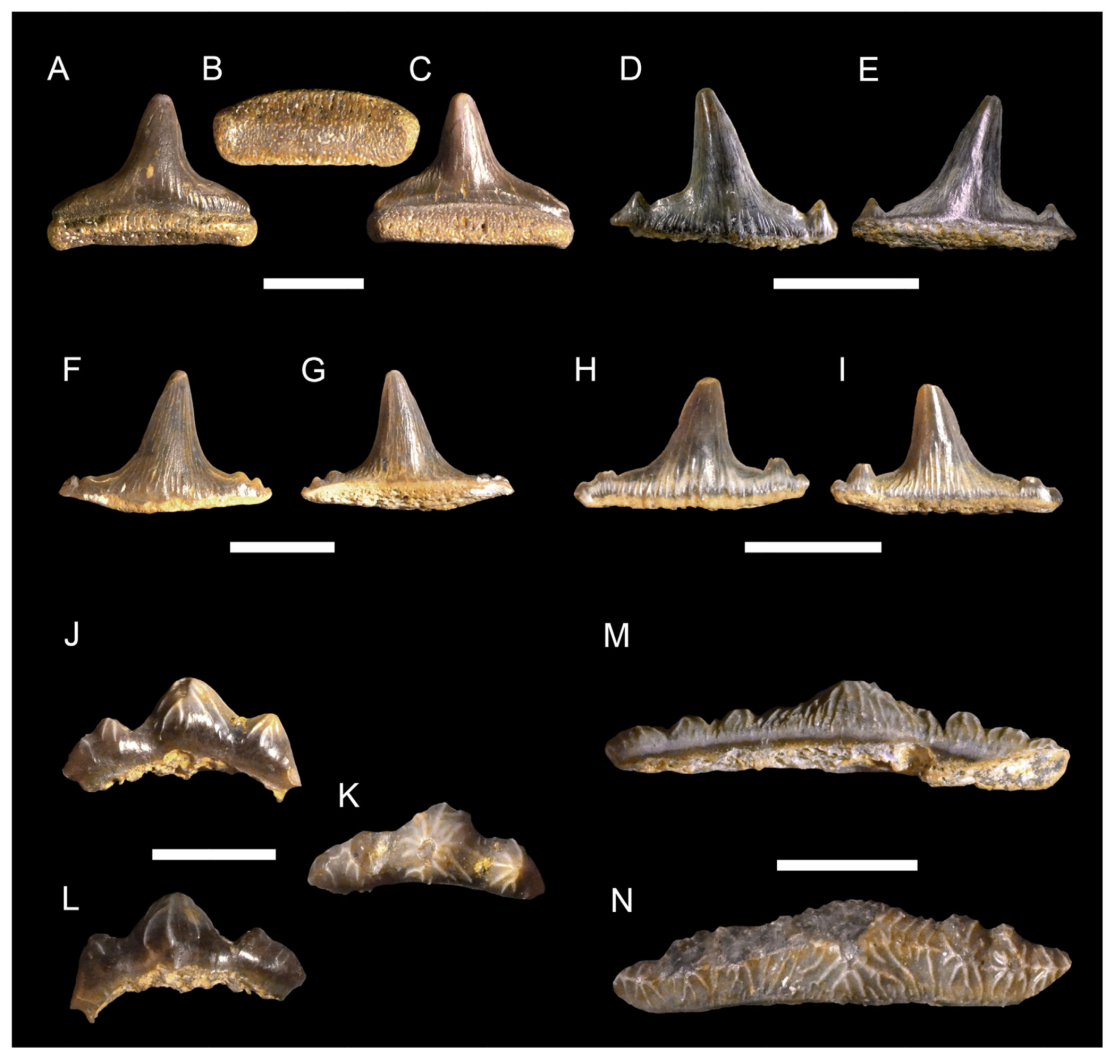
Hybodontiformes teeth from the Akkermanovka locality in the Orenburg Oblast, Russia. (A-I) *Meristodonoides* sp.: (A-C) MMO № 12260/333, (A) labial view, (B) basal view, (C) lingual view; (D-E) MMO № 12260/342, (D) labial view, (E) lingual view; (F-G) MMO № 12260/339, (F) labial view, (G) lingual view; (H-I) MMO № 12260/340, (H) labial view, (I) lingual view. (J-N) cf. *Polyacrodus* sp.: (J-L) MMO № 12260/335, anterior tooth, (J) labial view, (K) occlusal view, (L) lingual view; (M-N) MMO № 12260/334, lateral tooth, (M) labial view, (N) occlusal view. Scale bars = 5 mm.

**Fig. 5:**
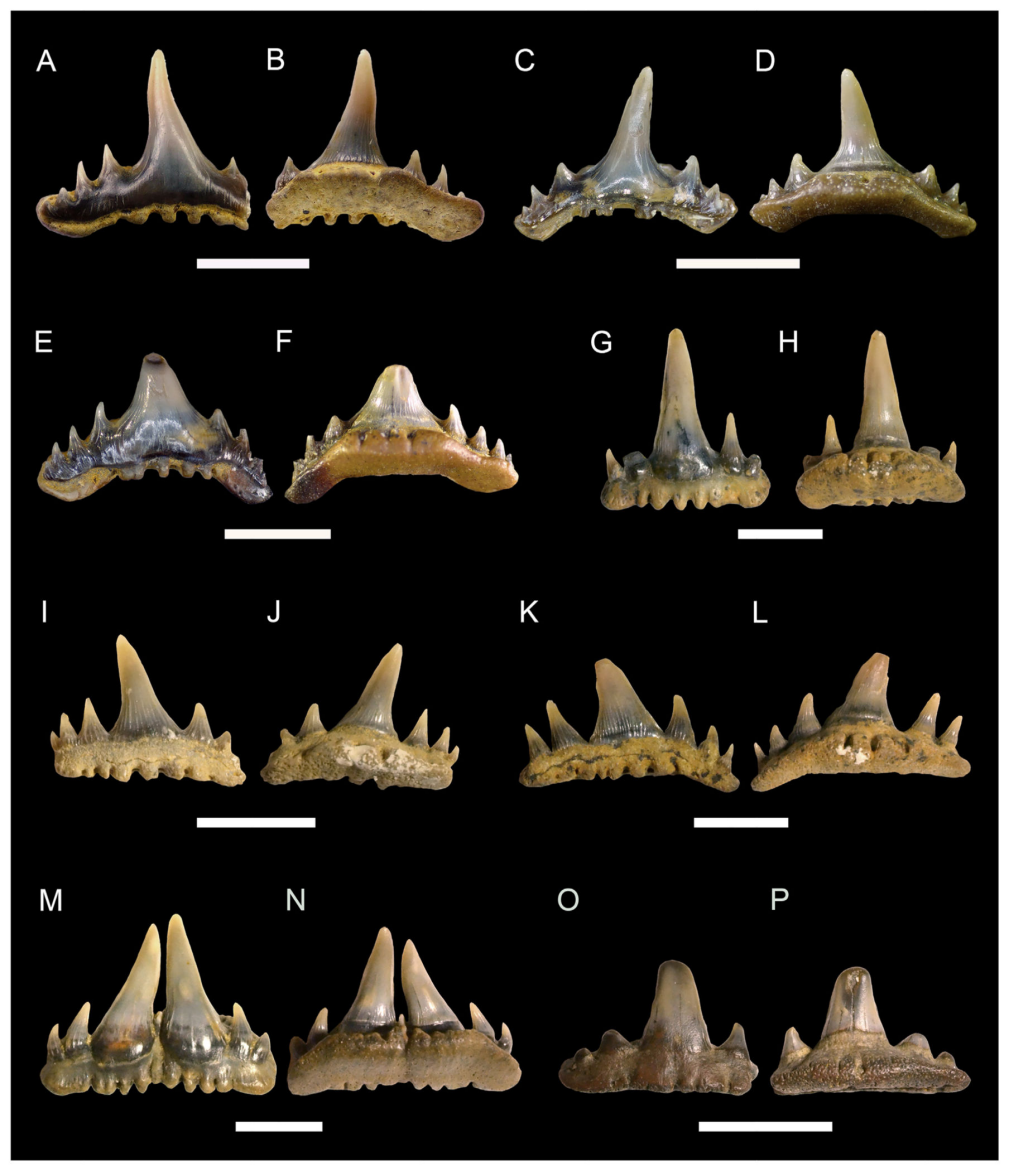
Synechodontiformes teeth from the Akkermanovka locality in the Orenburg Oblast, Russia. (A-F) *Synechodus* sp.: (A-B) MMO № 12260/330, (A) labial view, (B) lingual view; (C-D) MMO № 12260/329, (C) labial view, (D) lingual view; (E-F) MMO № 12260/331, (E) labial view, (F) lingual view. (G-N) *Paraorthacodus* cf. *andersoni*; (G-H) MMO № 12260/171, anterior tooth, (G) labial view, (H) lingual view; (I-J) MMO № 12260/172, lateral tooth, (I) labial view, (J) lingual view; (K-L) MMO № 12260/157, lateral tooth, (K) labial view, (L) lingual view; (M-N) MMO № 12260/177, pathological tooth, (M) labial view, (N) lingual view. (O-P) *Paraorthacodus* sp.: MMO № 12260/182, (O) labial view, (P) lingual view. Scale bars = 5 mm.

**Fig. 6:**
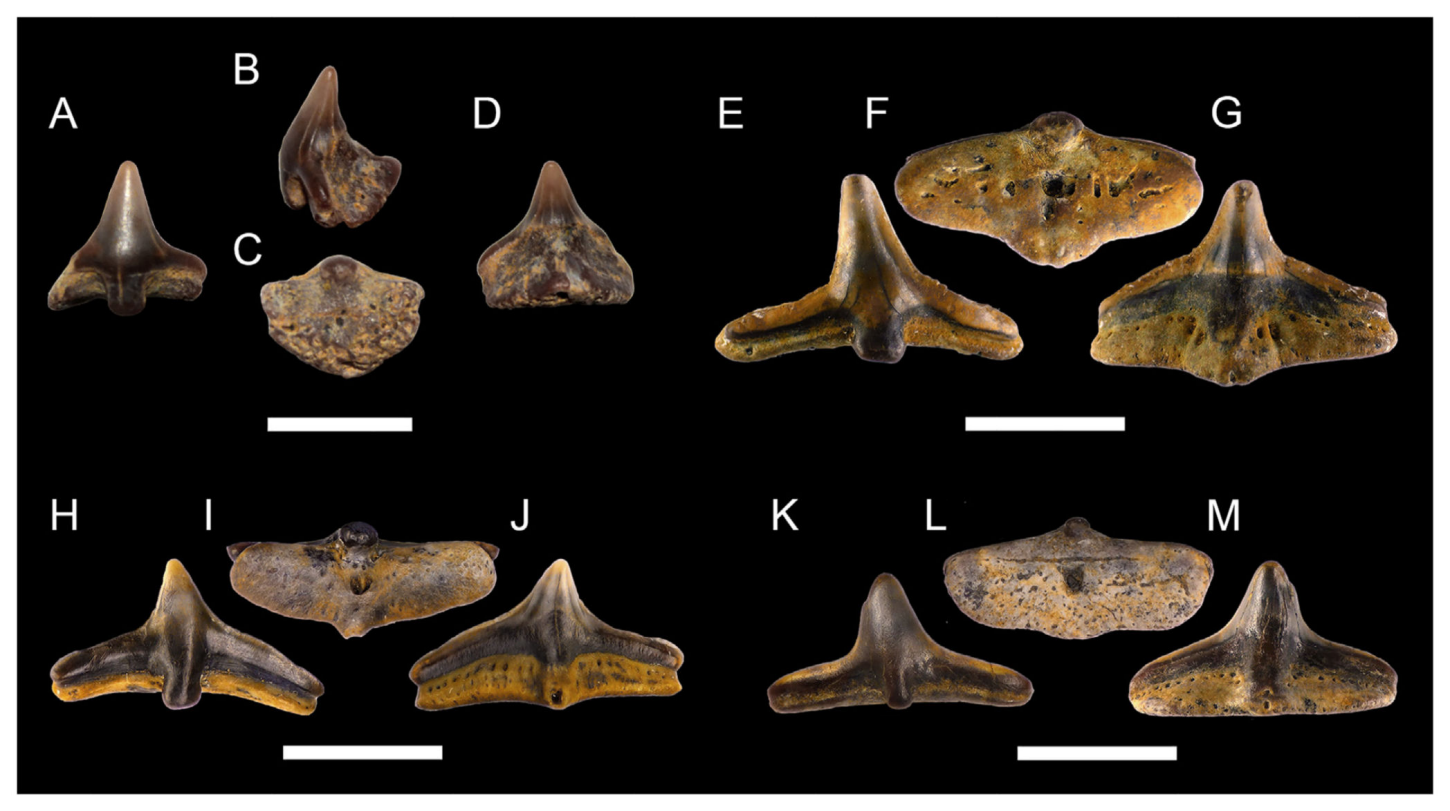
*Cederstroemia nilsi* teeth from the Akkermanovka locality in the Orenburg Oblast, Russia. (A-D) MMO № 12260/72, anterior tooth, (A) labial view, (B) profile view, (C) basal view, (D) lingual view; (E-G) MMO № 12260/71, lateral tooth, (E) labial view, (F) basal view, (G) lingual view; (H-J) MMO № 12260/66, lateral tooth, (H) labial view, (I) basal view, (J) lingual view; (K-M) MMO № 12260/65, lateral tooth, (K) labial view, (L) basal view, (M) lingual view. Scale bars = 5 mm.

**Fig. 7:**
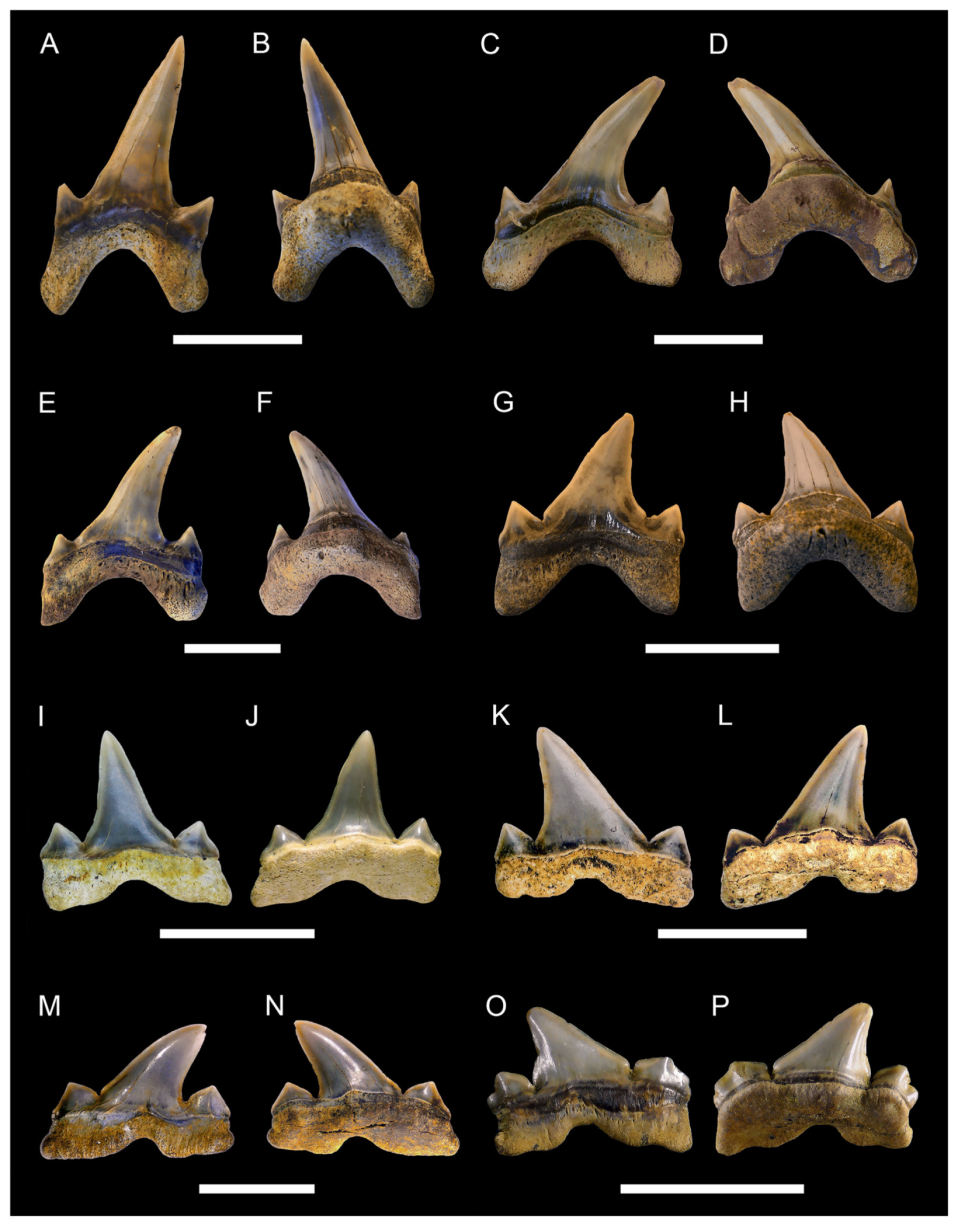
Lamniformes teeth from the Akkermanovka locality in the Orenburg Oblast, Russia. (A-H) *Archaeolamna* ex gr. *kopingensis*: (A-B) MMO № 12260/23, antero-lateral tooth, (A) labial view, (B) lingual view; (C-D) MMO № 12260/11, lateral tooth, (C) labial view, (D) lingual view; (E-F) MMO № 12260/9, lateral tooth, (E) labial view, (F) lingual view; (G-H) MMO N_ 12260/17, lateral tooth, (G) labial view, (H) lingual view. (I-P) *Cretalamna sarcoportheta*: (I-J) MMO № 12260/87, antero-lateral tooth, (I) labial view, (J) lingual view; (K-L) MMO № 12260/86, lateral tooth, (K) labial view, (L) lingual view; (M-N) MMO № 12260/75, lateral tooth, (M) labial view, (N) lingual view; (O-P) MMO № 12260/92, lateral tooth, (O) labial view, (P) lingual view. Scale bars = 10 mm.

**Fig. 8:**
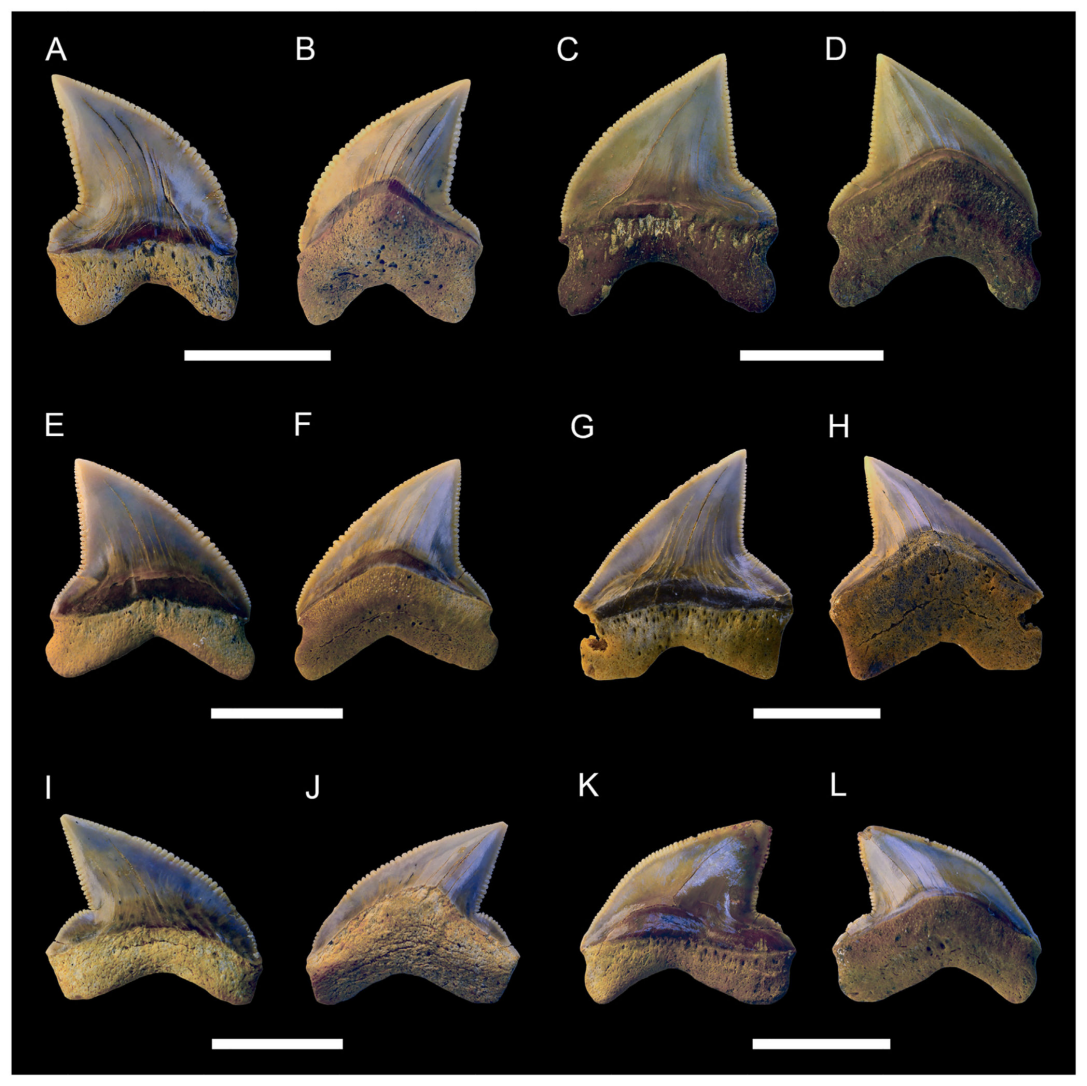
*Squalicorax kaupi* teeth from the Akkermanovka locality in the Orenburg Oblast, Russia. (A-B) MMO № 12260/251, (A) labial view, (B) lingual view; (C-D) MMO № 12260/256, (C) labial view, (D) lingual view; (E-F) MMO № 12260/228, (E) labial view, (F) lingual view; (G-H) MMO № 12260/227, (G) labial view, (H) lingual view; (I-J) MMO № 12260/229, (I) labial view, (J) lingual view; (K-L) MMO № 12260/261, (K) labial view, (L) lingual view. Scale bars = 10 mm.

**Fig. 10:**
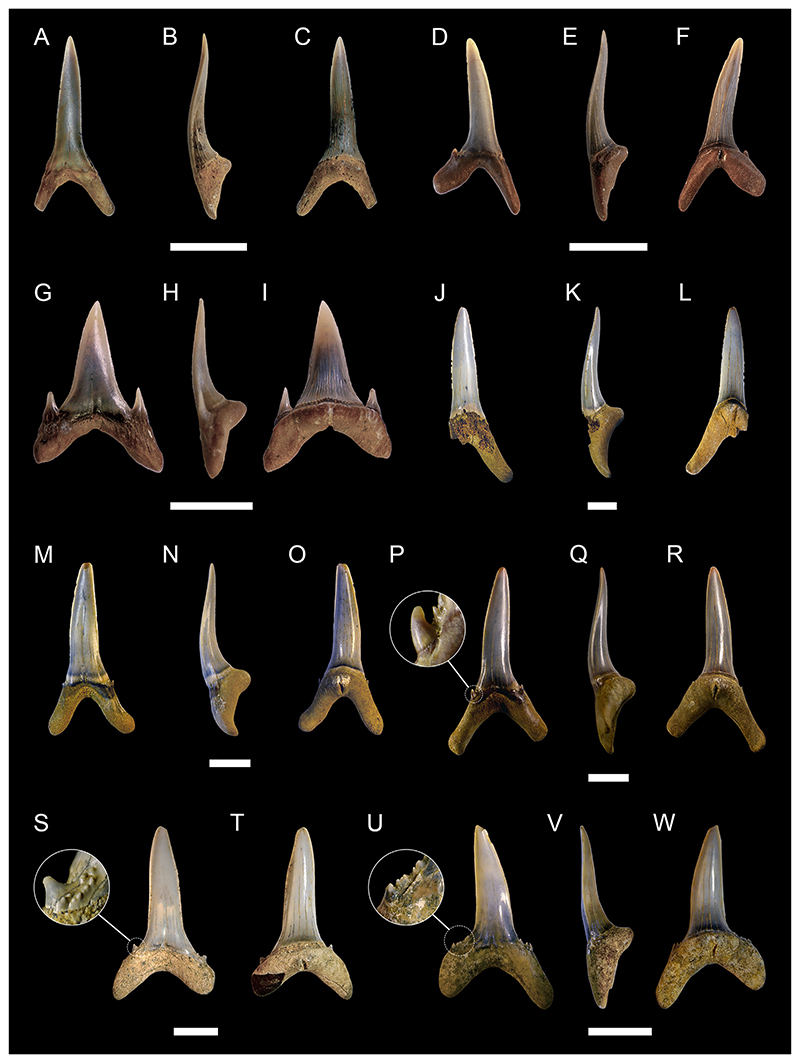
Lamniformes teeth from the Akkermanovka locality in the Orenburg Oblast, Russia. (A-I) *Scapanorhynchus rhaphiodon*: (A-C) MMO № 12260/199, anterior tooth, (A) labial view, (B) profile view, (C) lingual view; (D-F) MMO № 12260/198, antero-lateral tooth, (D) labial view, (E) profile view, (F) lingual view; (G-I) MMO № 12260/205, lateral tooth, (G) labial view, (H) profile view, (I) lingual view. (J-R) *Hispidaspis horridus*: (J-L) MMO № 12260/150, anterior tooth, (J) labial view, (K) profile view, (L) lingual view; (M-O) MMO № 12260/153, anterior tooth, (M) labial view, (N) profile view, (O) lingual view; (P-R) MMO № 12260/151, anterior tooth, (P) labial view, (Q) profile view, (R) lingual view. (S-W) *Hispidaspis* cf. *gigas*: (S-T) MMO № 12260/149, anterior tooth, (S) labial view, (T) lingual view; (U-W) MMO № 12260/152, antero-lateral tooth, (U) labial view, (V) profile view, (W) lingual view. Scale bars = 10 mm.

**Fig. 11:**
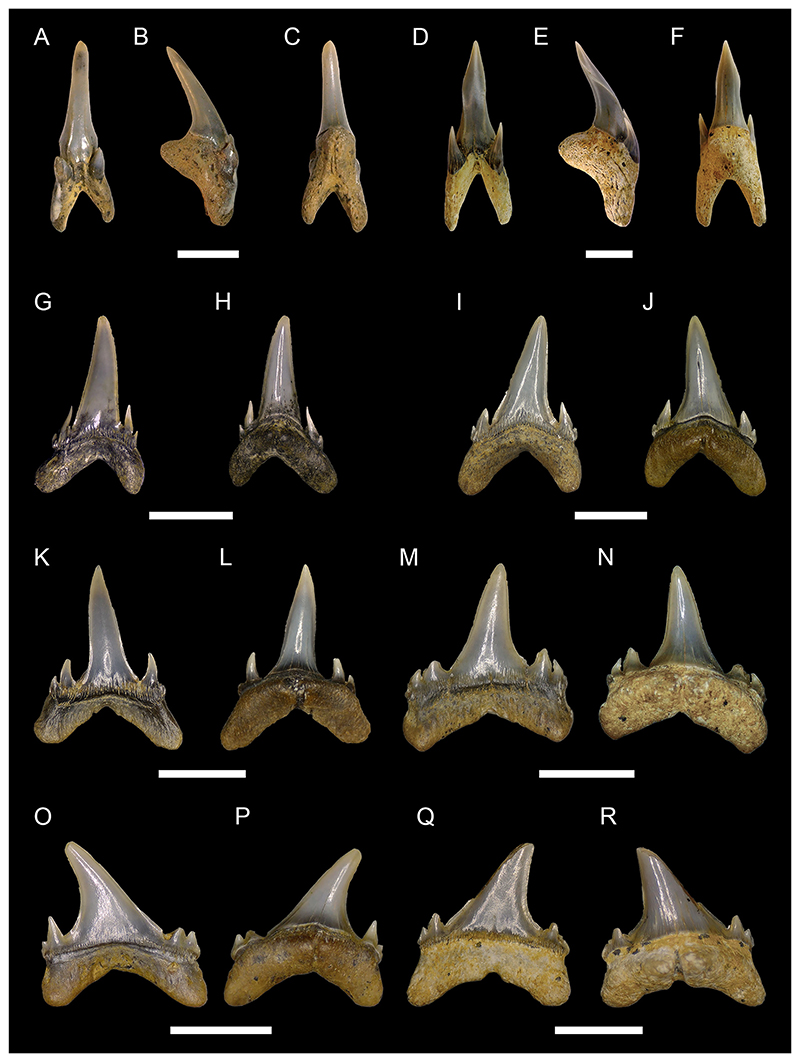
Lamniformes teeth from the Akkermanovka locality in the Orenburg Oblast, Russia. (A-F) *Pseudoscapanorhynchus compressidens*: (A-C) MMO № 12260/197, (A) labial view, (B) profile view; (C) lingual view; (D-F) MMO № 12260/185, (D) labial view, (E) profile view, (F) lingual view. (G-R) *Eostriatolamia segedini*: (G-H) MMO № 12260/97, anterior tooth, (G) labial view, (H) lingual view; (I-J) MMO № 12260/100, anterior tooth, (I) labial view, (J) lingual view; (K-L) MMO № 12260/98, anterior tooth, (K) labial view, (L) lingual view; (M-N) MMO № 12260/113, lateral tooth, (M) labial view, (N) lingual view; (O-P) MMO № 12260/114, lateral tooth, (O) labial view, (P) lingual view; (Q-R) MMO № 12260/115, lateral tooth, (Q) labial view, (R) lingual view. Scale bars = 5 mm.

**Fig. 12:**
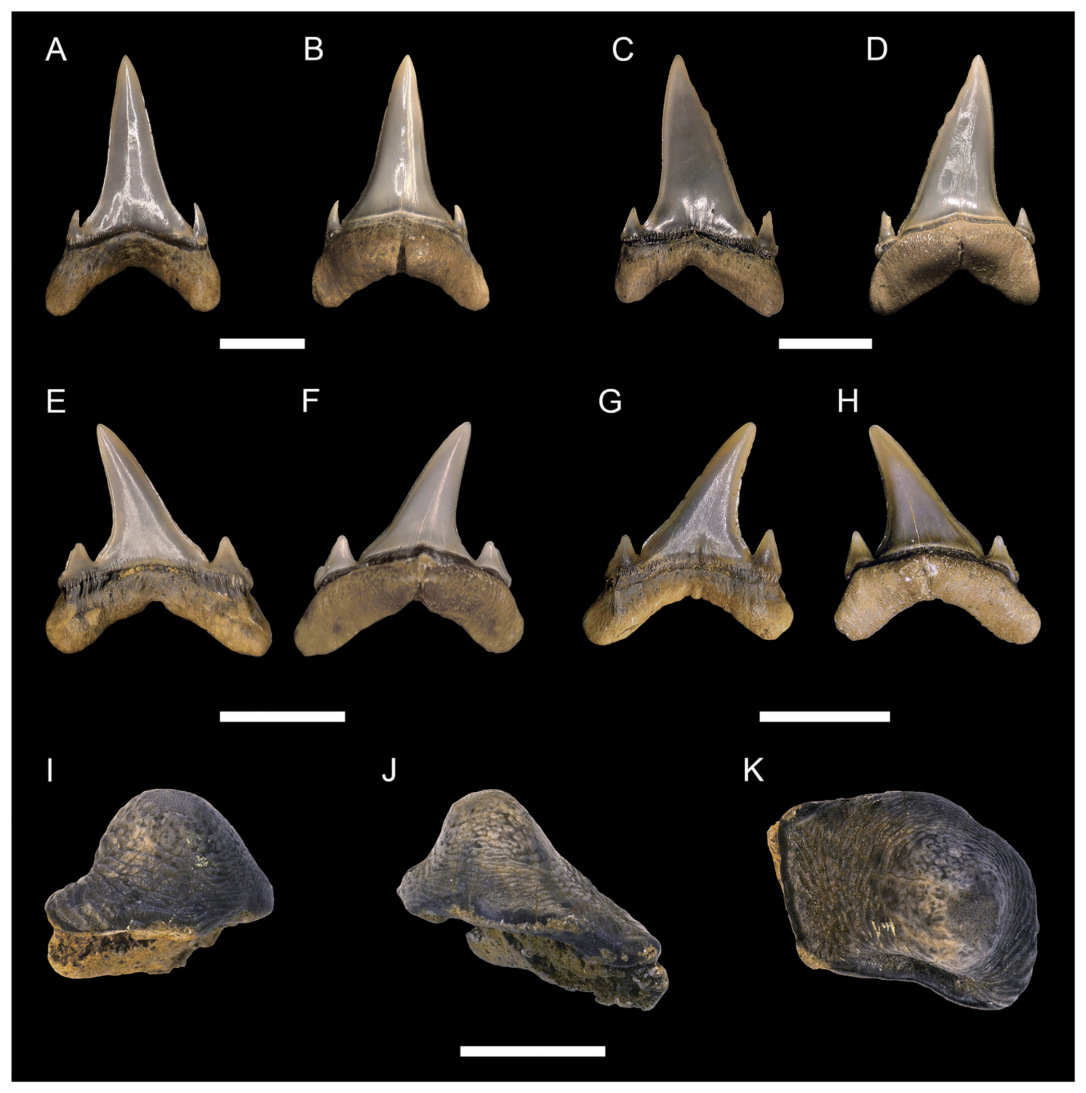
Lamniformes and Ptychodontiformes teeth from the Akkermanovka locality in the Orenburg Oblast, Russia. (A-H) *Eostriatolamia venusta*: (A-B) MMO № 12260/124, anterior tooth, (A) labial view, (B) lingual view; (C-D) MMO № 12260/131, antero-lateral tooth, (C) labial view, (D) lingual view; (E-F) MMO № 12260/144, lateral tooth, (E) labial view, (F) lingual view; (G-H) MMO № 12260/138, lateral tooth, (G) labial view, (H) lingual view. (I-K) *Ptychodus rugosus*: MMO № 12260/332, (I) mesial view, (J) anterior view, (K) occlusal view. Scale bars = 5 mm (A-H) and 10 mm (I-K).

**Fig. 14:**
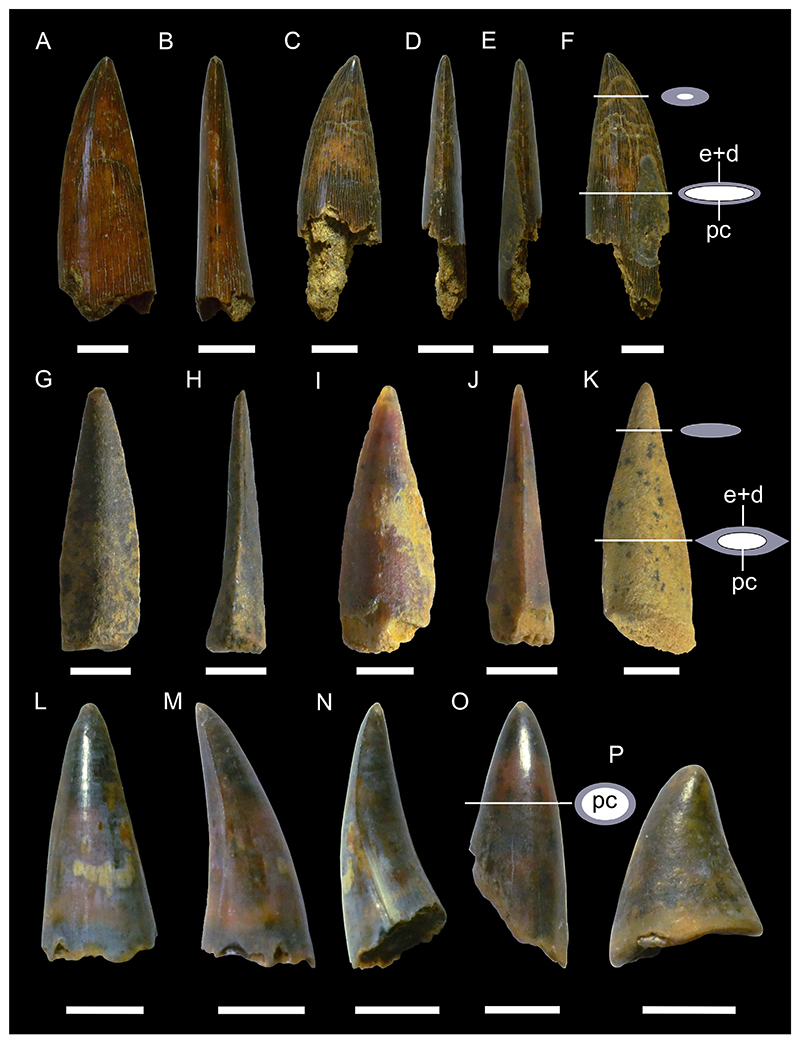
Pachycormiformes, Ichthyodectiformes, and Crossognathiformes remains from the Akkermanovka locality in the Orenburg Oblast, Russia. (A-F) *Protosphyraena* sp.: (A–B) MMO № 12260/430, tooth crown, (A) profile (labial) view, (B) posterior view; (C-F) MMO № 12260/431, incomplete tooth crown with mesial-apical wear facet, (C) profile (lingual) view, (D) posterior view, (E) anterior view, (F) profile (labial) view. (G-K) Saurodontidae indet.: (G-H) MMO № 12260/401, tooth crown, (G) profile (labial) view, (H) anterior view; (I-J) MMO № 12260/402, tooth crown, (I) profile view, (J) anterior view; (K) MMO № 12260/407, possibly reworked tooth crown with dendrite growth in profile view. (L-O) cf. *Pachyrhizodus* sp.: (L–N) MMO № 12260/415, tooth crown, (L) anterior view, (M) profile (labial) view, (N) profile (lingual) view; (O) MMO № 12260/416, large incomplete tooth crown in anterior view. (P) Pachyrhizodontidae indet. MMO № 12260/412, crown with unusual height to width ratio in profile view. *Abbreviations*: e = enamel; d = dentine layer; pc = pulp cavity. Cross sections show cross section shape and relative thicknesses between the enamel and dentine compared to the pulp cavity. Scale bars = 5 mm (A–F) and 3 mm (G–P).

**Fig. 15:**
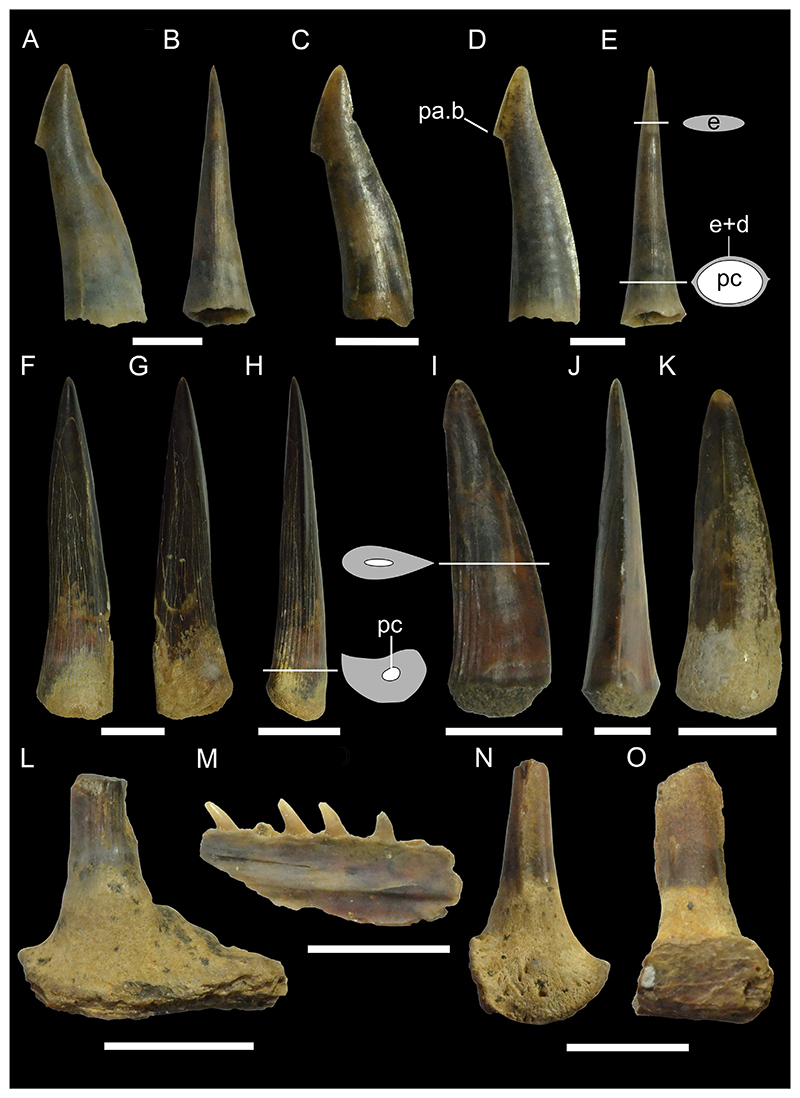
Aulopiformes remains from the Akkermanovka locality in the Orenburg Oblast, Russia. (A–E) Alepisauroidei indet.: (A-B) MMO № 12260/421, anterior fang, (A) profile view, (B) anterior view; (C) MMO № 12260/423, anterior fang in profile view; (D-E) MMO № 12260/420, complete tooth crown, (D) profile view, (E) anterior view. (F-H), *Enchodus petrosus*, MMO № 12260/432, isolated dermopalatine fang, (F) profile (labial) view, (G) profile (lingual view), (H) anterior view. (I–K) *Enchodus* cf. *gladiolus*: (I–J) MMO № 12260/384, isolated dermopalatine fang, (I) profile (labial) view, (J) anterior view; (K) MMO № 12260/357, worn dermopalatine tooth in profile view. (L) *Enchodus ferox*, MMO № 12260/409, dermopalatine bone fragment with associated, albeit broken tooth crown. (M-O) *Enchodus* spp. indet.: (M) MMO № 12260/435 jaw fragment (premaxilla?) preserving 4 original tooth crowns in labial view; (N) MMO № 12260/410, broken dermopalatine tooth base in anterior view; (O) MMO № 12260/470, broken indeterminate jaw fragment with base of broken tooth base in profile view. *Abbreviations*: e = enamel layer; d = dentine layer; pa.b = postapical barb; pc = pulp cavity. Cross section drawings show the shape and relative thickness of the enamel and dentine compared to the pulp cavity. Scale bars = 5 mm (A–K) and 3 mm (L–O).

**Fig. 16:**
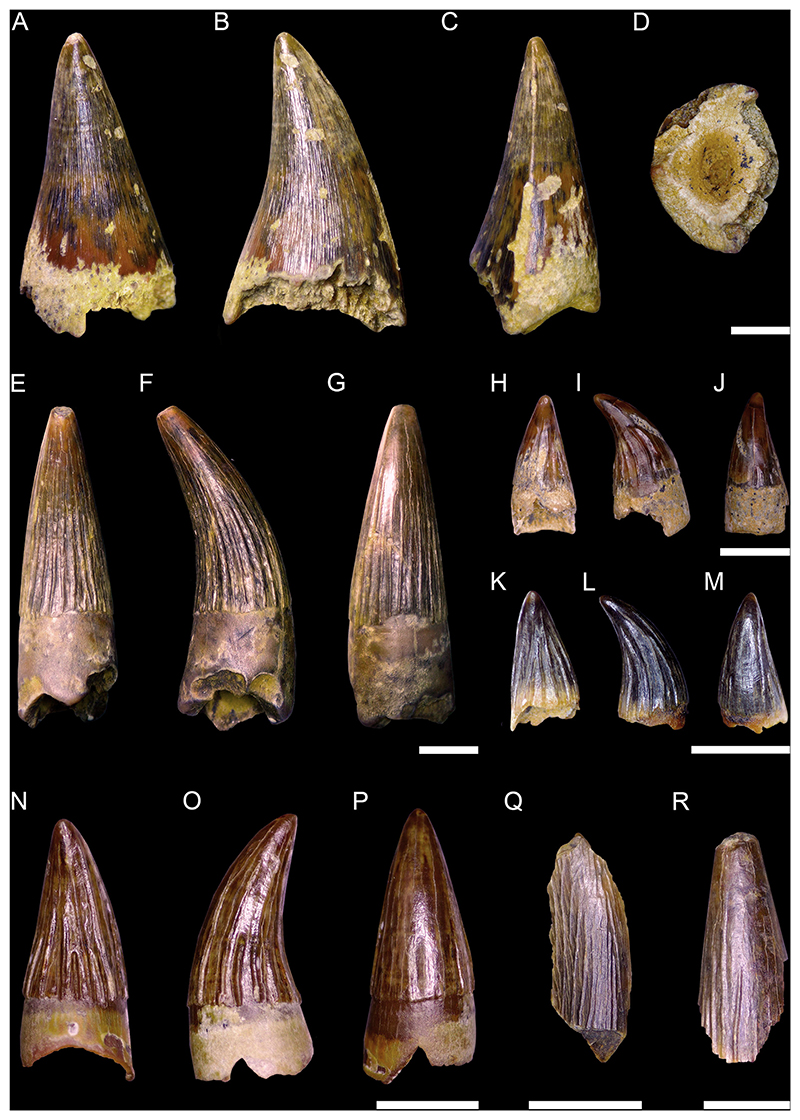
Marine reptile teeth from the Akkermanovka locality in the Orenburg Oblast, Russia. (A-D) Tylosaurinae indet.: MMO № 12260/453, (A) distal view, (B) profile (labial) view, (C) mesial view, (D) basal view. (E-P) Polycotylidae indet.: (E-G) MMO № 12260/454, (E) distal view, (F) profile view, (G) mesial view; (H-J) MMO № 12260/457, (H) distal view, (I) profile view, (J) mesial view; (K-M) MMO № 12260/455, (K) distal view, (L) profile view, (M) mesial view; (N-P) MMO № 12260/456, (N) distal view, (O) profile view, (P) mesial view. (Q-R) Plesiosauria indet.: (R) MMO № 12260/459, tooth fragment; (S) MMO № 12260/458, tooth fragment. Scale bars = 5 mm.

**Table 1:** Geographic distribution of the taxa found in the marine vertebrate assemblage from Akkermanovka (Orenburg Oblast, Southern Ural) during the Santonian and Campanian stages (Late Cretaceous). Only taxa which have been identified on species or genus level are considered here. Occurrence data was compiled from the following references: 1, Averianov (2001); 2, Averianov (1991); 3, Nessov et al. (1994); 4, Case (1978b); 5, Siverson (1992a); 6, Kriwet et al. (2006); 7, Cappetta (2012); 8, Zhelezko (1987); 9, Kitamura (2019); 10, Case (1987); 11, Siverson (1995); 12, Zhelezko (1990); 13, Siversson et al. (2015); 14, Priem (1907); 15, Sokolov (1978); 16, Case et al. (2001); 17, Antunes and Cappetta (2002); 18, da Silva (2007); 19, Yabe and Obata (1930); 20, Hamm (2020); 21, Kear et al. (2009); 22, McNamara et al. (1993); 23, Applegate (1970); 24, Everhart (2005a); 25, Wiffen (1983); 26, Schein and Lewis (2007); 27, Shimada and Fielitz (2006).

	Africa	Antarctica	Asia	Australia	Europe	North America	South America	Oceania	References
*Elasmodus* sp.			X		X				1
*Ischyodus yanschini*			X		X				2
*Meristodonoides* sp.			X		X	X			3, 4
cf. *Polyacrodus* sp.			X		X				3
*Paraorthacodus* cf. *andersoni*					X	X			5
*Paraorthacodus* sp.		X	X		X	X			5, 6, 7
*Synechodus* sp.			X		X	X			8, 9, 10
*Cederstroemia nilsi*					X				11
*Acrolamna acuminata*					X				12
*Archaeolamna* ex gr. *kopingensis*			X		X	X			4, 7
*Cretalamna sarcoportheta*					X	?			13
*Cretoxyrhina mantelli*	X		X		X	X	X		4, 7, 8, 14
*Eostriatolamia segedini*			X		X				8
*Eostriatolamia venusta*			X		X				4, 8
*Hispidaspis horridus*			X		X				15
*Hispidaspis* cf. *H. gigas*			X		X				8
*Pseudocorax laevis*			X		X	X			7, 8
*Pseudoscapanorhynchus compressidens*					X				
*Scapanorhynchus rhaphiodon*	X		X		X	X			8, 14, 16
*Squalicorax kaupi*	X		X		X	X	X		4, 8, 17, 18
*Ptychodus rugosus*			X		X	X			19, 20
*Protosphyraena* sp.			X	X	X	X			21, 22, 23
cf. *Pachyrhizodus* sp.					X	X		X	24, 25
*Enchodus ferox*					X	X			26
*Enchodus petrosus*					X	X			27
*Enchodus* cf. *gladiolus*					X	X			27

## Data Availability

All data generated and analysed during this study are given in the manuscript.
